# A Highly Charged Positive Cage Causes Simultaneous Enhancement of Type‐II and O_2_‐Independent‐Type‐I Photodynamic Therapy via One‐/Two‐Photon Stimulation and Tumor Immunotherapy via PANoptosis and Ferroptosis

**DOI:** 10.1002/smsc.202400220

**Published:** 2024-07-04

**Authors:** Xiao‐Dong Zhang, Hui‐Juan Yu, Shao‐Qi Guan, Yu‐Lin Lu, Yu Zhang, Yin‐Hui Huang, Ya‐Ping Wang, Chen‐Hui Liu, Zhong‐Min Cao, Yu‐Han Qin, Mei Pan, Jun Shen, Cheng‐Yong Su

**Affiliations:** ^1^ Department MOE Laboratory of Bioinorganic and Synthetic Chemistry, GBRCE for Functional Molecular Engineering, LIFM, IGCME School of Chemistry Sun Yat‐Sen University Guangzhou 510275 China; ^2^ Guangdong Key Laboratory of Animal Conservation and Resource Utilization, Guangdong Public Laboratory of Wild Animal Conservation and Utilization, Institute of Zoology Guangdong Academy of Sciences Guangzhou 510275 China; ^3^ Department of Radiology, Guangdong Provincial Key Laboratory of Malignant Tumor Epigenetics and Gene Regulation, Medical Research Center, Sun Yat‐Sen Memorial Hospital Sun Yat‐Sen University Guangzhou 510030 China

**Keywords:** cationic metal‐organic cages, ferroptoses, one‐/two‐photon Type‐I/II photodynamic therapies, PANoptoses, tumor immunotherapies

## Abstract

To solve the oxygen dependence problem of photodynamic therapy (PDT), it is critical to explore photosensitizers that do not rely on O_2_ molecule to generate reactive oxygen species (ROS). Herein, a stable cationic metal‐organic cage [Pd_6_(RuL^oz^_3_)_8_](BF_4_)_28_ (MOC‐88) that possesses high +28 charges is designed. The cage‐confined positive microenvironment enables efficient generation of hydroxyl radicals and improved yield of the singlet oxygen under one‐/two‐photon excitation, showing excellent performance to concurrently enhance Type‐II and O_2_‐independent‐Type‐I PDT. Moreover, the effective ROS production and robust lipid peroxidation trigger a series of signaling pathways (inflammasome, cyclic guanosine monophosphate–adenosine monophosphate synthase stimulator of interferon genes, and NF‐κB) to evoke PANoptosis and ferroptosis in tumor cells, enabling MOC‐88 to simultaneously cause the loss of cell membrane integrity, release a series of inflammatory cytokines and damage‐associated molecular patterns, stimulate the maturation and antigen presentation ability of dendritic cells, and ultimately activate T‐cell‐dependent adaptive immunity in vivo to inhibit tumor growth.

## Graphical abstract

As an exquisite example of metal‐organic cage (MOC), MOC‐88 induced simultaneous Type‐II and O_2_‐independent‐Type‐I photodynamic therapy via one‐/two‐photon stimulation and tumor immunotherapy via PANoptosis and ferroptosis.

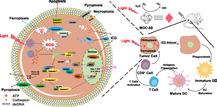

## 1 Introduction

As a promising cancer treatment method, photodynamic therapy (PDT) works by exposing a photosensitizer (PS) to light source to interact with molecular oxygen (O_2_) and generate cytotoxic reactive oxygen species (ROS).^[^ ^1^ ^]^ PDT can be divided into Type‐I PDT and Type‐II PDT,^[^ ^2^ ^]^ and its effectiveness is mainly influenced by factors such as the oxygen concentration of the tumor,^[^ ^3^ ^]^ light,^[^ ^4^ ^]^ and the characteristics of PSs.^[^ ^5^ ^]^ For solid tumors, the hypoxic microenvironment (*p*O_2_ < 5 mm Hg) renders resistance to O_2_‐based Type‐II PDT process that mainly produces singlet oxygen (^1^O_2_).^[^ ^6^ ^]^ In contrast, Type‐I PDT shows less O_2_ dependence to produce ROS of superoxide (O_2_^−•^), hydrogen peroxide (H_2_O_2_), and hydroxyl radicals (•OH).^[^ ^7^ ^]^ Designing O_2_‐independent PSs to generate ROS under hypoxic conditions holds a great promise to fundamentally address the O_2_‐dependence issue in PDT. Since the Type‐I and Type‐II processes of PSs usually compete with each other, promotion of both Type I and Type II is hard to achieve in PDT.^[^ ^8^ ^]^ By virtue of supramolecular design, the distance between the electron–donor and the PS that acts as electron acceptor can be shortened through co‐assembly/self‐assembly^[^ ^9^ ^]^ and/or host–guest interactions,^[^ ^10^ ^]^ which were able to inhibit Type‐II process, promote electron transfer, and achieve the transition from Type‐II to Type‐I PDT. However, there remains a challenge to boost the Type‐I process without impacting the Type‐II process, namely concurrently enhancing the Type‐I and Type‐II PDT. Due to water being the most abundant compound in tissues, it is used to replace O_2_ as an oxygen source for the generation of •OH and is a potential pathway to achieve this goal.

**Scheme 1:**
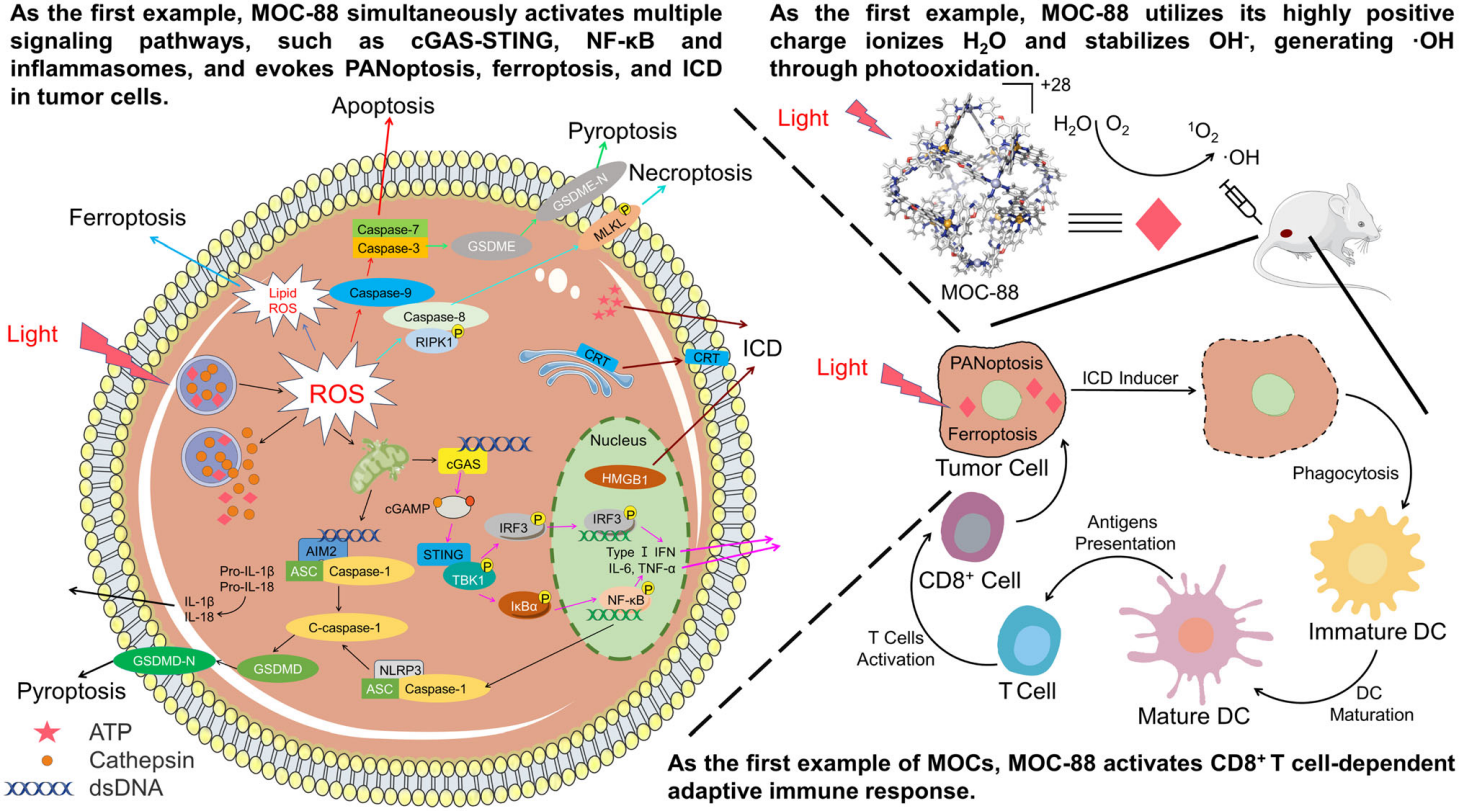
The schematic diagram showing generation of ROS of singlet ^1^O_2_ and hydroxyl radicals under light stimulation by MOC‐88, and induction of tumor cell PANoptosis and ferroptosis, leading to the occurrence of immunogenic cell death (ICD) in cells to achieve immunotherapy. Some cartoon components were from www.figdraw.com for model drawing. Caspase, cysteinyl aspartate specific proteinase; GSDMD, Gasdermin D; GSDMD‐N, N‐term cleaved Gasdermin‐D; GSDME, Gasdermin E; GSDME‐N, N‐term cleaved Gasdermin‐E; RIPK1, receptor‐interacting protein kinase 1; MLKL, mixed lineage kinase domain‐like pseudokinase; AIM2, absent in melanoma 2; ASC, apoptosis‐associated speck‐like protein containing a CARD; NLRP3, NOD‐like receptor thermal protein domain associated protein 3; cGAS, cyclic guanosine monophosphate (GMP)–adenosine monophosphate (AMP) synthase; cGAMP, cyclic GMP‐AMP; STING: stimulator of interferon genes; TBK1, TANK‐binding kinase 1; IRF3, interferon regulatory factor 3; IκB*α*, NF‐kappa‐B inhibitor alpha; NF‐κB, nuclear factor κB; IL, interleukin; TNF‐*α*, Tumor necrosis factor *α*; IFN, interferon; CRT, Calreticulin; HMGB1, High mobility group box‐1 protein; P, phosphorylated; DC, Dendritic cell.

Programmed cell death (PCD) is a type of cell death that is precisely regulated by specific signaling pathways.^[^ ^11^ ^]^ In recent years, significant progress has been made in the field of PCD research, and scientists have successively revealed various new types of PCD based on their unique molecular mechanisms, including apoptosis,^[^ ^12^ ^]^ pyroptosis,^[^ ^13^ ^]^ necroptosis,^[^ ^14^ ^]^ autophagy,^[^ ^15^ ^]^ ferroptosis,^[^ ^16^ ^]^ and cuproptosis.^[^ ^17^ ^]^ The cell death was previously considered to independently operate in isolation, until the introduction of the PANoptosis concept in 2016.^[^ ^18^ ^]^ PANoptosis is an inflammatory PCD that is regulated by the PANoptosome complex with the key characteristics of pyroptosis, apoptosis, and necroptosis (**Scheme** **1**).^[^ ^19^ ^]^ The components that make up the PANoptosome were found to vary depending on the triggering factors.^[^ ^20^ ^]^ Therefore, PANoptosome has become an entry point of research to regulate PANoptosis, motivating intensive investigations on its triggering factors, regulatory mechanisms, and assembly strategies, especially in tumor prevention and treatment.

In addition, there may be a certain connection between ferroptosis and PANoptosis.^[^ ^21^ ^]^ Ferroptosis is a novel type of iron‐dependent regulated cell death, which is mainly caused by glutathione (GSH) depletion and reduced GSH peroxidase (GPX4) activity, which ultimately leads to lipid peroxidation (LPO) increase.^[^ ^22^ ^]^ Ferroptosis is involved in the pathophysiology of a group of diseases, including neurodegeneration, cardiovascular disease, metabolic dysfunction, and tumor biology.^[21b]^

The cyclic guanosine monophosphate–adenosine monophosphate (GMP–AMP) synthase stimulator of interferon genes (cGAS–STING) signaling pathway originally functioned as host defense, but in recent years its research on antitumor immune responses have attracted increasing attention.^[^ ^23^ ^]^ As a cytoplasmic DNA sensor, cGAS generates the second messenger 2′,3′‐cyclic GMP–AMP (cGAMP) when detecting intracellular DNA, thereby activating STING in the endoplasmic reticulum. Subsequently, activated STING is transferred from the endoplasmic reticulum to the golgi apparatus. During this process, STING undergoes ubiquitination and recruits TANK‐binding kinase 1 (TBK1) protein. TBK1 protein further phosphorylates and activates interferon regulatory factors (IRFs) and nuclear factor‐κB (NF‐κB). Activation of these transcription factors in turn promotes the expression of Type‐I interferons (IFNs) or pro‐inflammatory cytokines.^[^ ^24^ ^]^ As a promising anticancer strategy, the cGAS–STING signaling pathway has developed rapidly in recent years. However, there are still relatively few reports on small molecule activators and photo‐activators of the STING pathway.^[^ ^25^ ^]^

**Figure 1:**
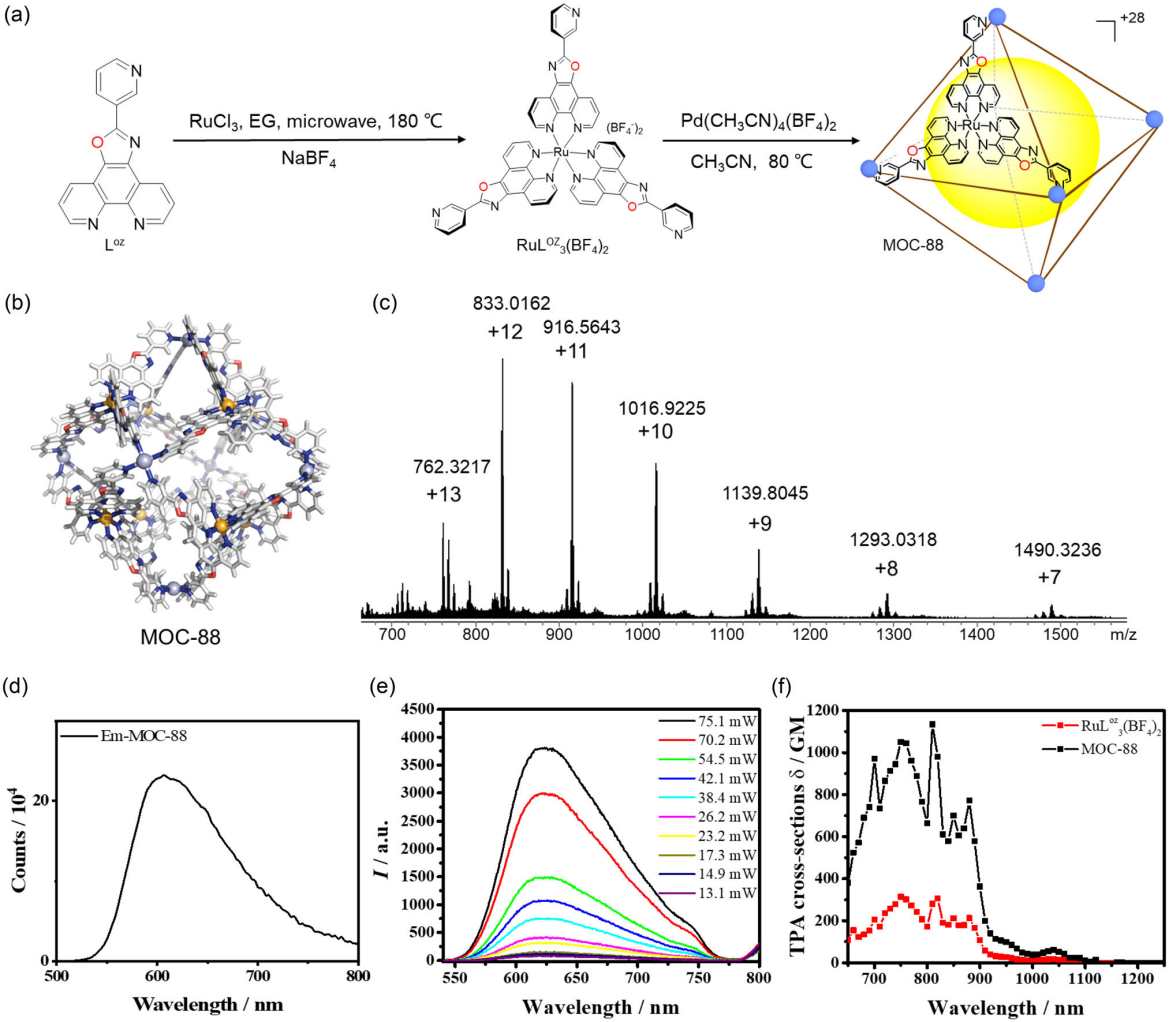
a) Synthetic route of MOC‐88. EG refers to ethylene glycol, and only one of eight (RuL^oz^_3_)^2+^ metalloligands is shown on the octahedral cage faces. b) Single‐crystal structure of MOC‐88, omitting BF_4_^−^ counter anions. c) HR‐ESI‐TOF‐MS spectra of MOC‐88 in CH_3_CN (0.2 mM). d) Photoluminescent emission of MOC‐88 in PBS solution (4 μM, *λ*_ex_ = 458 nm). e) The power‐dependent emission spectra of MOC‐88 in DMSO solution (0.4 mM, *λ*_ex_ = 810 nm). f) TPA cross sections of RuL^oz^_3_(BF_4_)_2_ (3.2 mM) and MOC‐88 (0.4 mM) in DMSO solution.

Metal‐organic cages (MOCs)^[^ ^26^ ^]^ represent a new type of porous supramolecular carriers that show fruitful prospects in catalysis,^[^ ^27^ ^]^ molecular recognition,^[^ ^28^ ^]^ drug delivery,^[^ ^29^ ^]^ and biomedical applications.^[^ ^30^ ^]^ We previously found that the highly charged cationic MOC‐16^[^ ^31^ ^]^ could achieve pH‐dependent one‐ and two‐photon excitation imaging of cell membranes by the aid of its electrostatic and hydrophobic–lipophilic interactions.^[^ ^32^ ^]^ Herein, we designed a new highly positive cage, MOC‐88 through stepwise assembly of RuL^oz^_3_(BF_4_)_2_ metalloligands with Pd^2+^ ions (**Figure** **1**). This cage can maintain neat +28 valent state due to the replacement of the imidazole (imidazolium p*K*_a_ = 7.0)^[^ ^33^ ^]^ groups on MOC‐16 with oxazole (oxazonium p*K*_a_ = 0.8)^[^ ^34^ ^]^ motifs to prevent deprotonation. Consequently, MOC‐88 features much improved solubility in water, showing log*P*_o/w_ value (lipophilicity) of −1.52 compared to 1.66 of MOC‐16.^[^ ^32^ ^]^ Since the proteins on cell membrane and surface usually exhibit negative charges, the highly positive charges and good water solubility of MOC‐88 benefit its interactions with the cell membrane and surface proteins, and ultimate internalization into the cell.^[^ ^35^ ^]^ Notably, assembly of eight (RuL^oz^_3_)^2+^ metalloligands, which are primary Type‐II PSs, into the highly charged MOC‐88 could enhance Type‐II and Type‐I processes in aerobic environment, and strikingly create O_2_‐independent Type‐I PDT ability under anaerobic conditions. With both one‐ and two‐photon excitation, MOC‐88 can generate abundant ROS to evoke PANoptosis and ferroptosis in cells and, meanwhile, trigger cell immunogenic death in vivo (Scheme 1). To the best of our knowledge, this work presents the first achievement of O_2_‐independent Type‐I process using MOC and its application in PANoptosis for efficient tumor immunotherapy.^[^ ^36^ ^]^

## 2 Results and Discussion

### 2.1 Synthesis, Characterization, and PDT Mechanism

The synthetic details and characterization of the new [Pd_6_(RuL^oz^_3_)_8_](BF_4_)_28_ cage (MOC‐88) are described in Supporting Information (Figure 1 and S1–S11, Supporting Information). The cationic cage structure in solution was confirmed by the high‐resolution electrospray ionization time‐of‐flight mass spectrometry (HR–ESI–TOF–MS), which displays a series of peaks in positive charge states ([MOC‐88+*x*BF_4_^−^]^(28−*x*)+^, *x* = 15–21, Figure 1c). The single crystals qualified for X‐ray diffraction determination were obtained, revealing a truncated octahedral cage structure composed of eight triangular (RuL^oz^_3_)^2+^ metalloligands, six 4‐coordinated Pd^2+^ ions, and 28 BF_4_^−^ counter anions (Figure 1b and Table S1, Supporting Information).

The ultraviolet–visible (UV–vis) absorption spectra of RuL^oz^_3_(BF_4_)_2_ metalloligand and MOC‐88 cage are comparable in a pH 7.4 phosphate buffered saline (PBS) solution, showing two main absorption bands with the strong spin‐allowed intra‐ligand transition around 250–360 nm, and the weak metal‐ligand charge‐transfer transition (MLCT) around 360–550 nm (Figure S12, Supporting Information). Upon one‐photon excitation in 450–460 nm range, RuL^oz^_3_(BF_4_)_2_ and MOC‐88 display similar red emission centered at 610 nm (Figure 1d and S13, Supporting Information) with quantum yields of 8.7% and 8.8% in Dimethyl sulfoxide (DMSO) solution, respectively (Table S2, Supporting Information). Due to the coordination of Pd^2+^, the phosphorescence lifetime of MOC‐88 decreases to nearly half of RuL^oz^_3_(BF_4_)_2_ (Figure S14, Supporting Information). The two‐photon absorption (TPA) cross sections of RuL^oz^_3_(BF_4_)_2_ and MOC‐88 were measured from 650 to 1200 nm with 10 nm intervals using femtosecond laser pulses. As shown in Figure 1e,f and S15, Supporting Information, MOC‐88 displays a large TPA cross section of 1134 GM, much larger than the 306 GM for RuL^oz^_3_(BF_4_)_2_ under the same conditions, suggesting that the assembly of multiple metalloligands into a single cage can give rise to a higher two‐photon cross section.^[^ ^37^ ^]^ The stability of RuL^oz^_3_(BF_4_)_2_ and MOC‐88 in solution was evaluated in PBS and DMSO*‐d*_6_/D_2_O solutions in different pH values. The UV–vis absorption, phosphorescence, and ^1^H‐nuclear magnetic resonance (NMR) measurements disclosed that RuL^oz^_3_(BF_4_)_2_ remains intact within the pH 1–12 range, while MOC‐88 keeps stable within the pH 1–9 range, owing to the hydrolysis of Pd^2+^ ions in a strong basic medium (Figure S16 and S17, Supporting Information). In addition, Figure S18, Supporting Information, shows that MOC‐88 keeps stable in PBS, dulbecco's modified eagle medium (DMEM), and complete DMEM solutions for at least 48 h.

**Figure 2:**
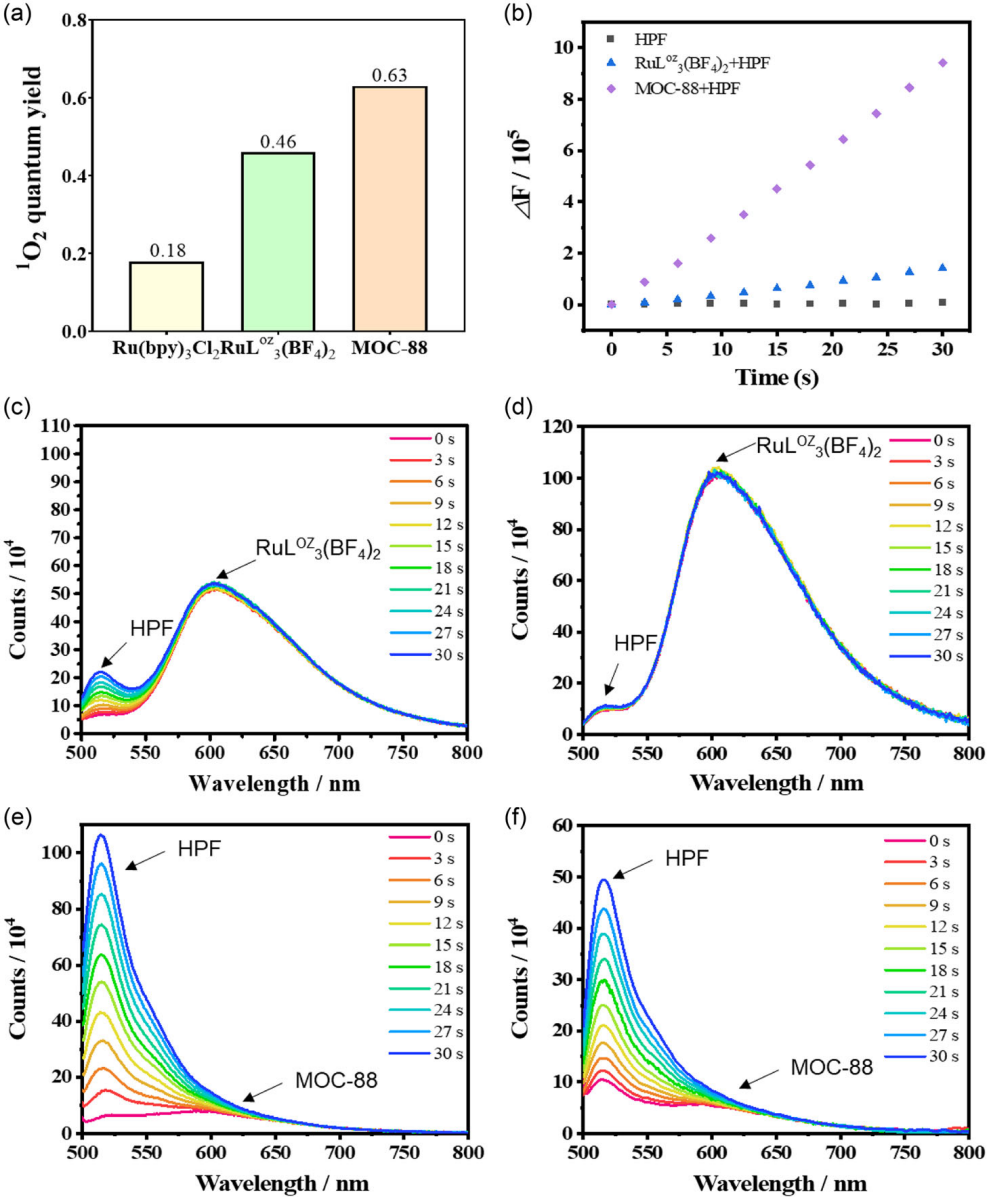
a) Comparison of singlet ^1^O_2_ quantum yields of RuL^oz^_3_(BF_4_)_2_ and MOC‐88 in PBS, using Ru(bpy)_3_Cl_2_ as the standard. b) The rate at which RuL^oz^_3_(BF_4_)_2_ (8 μM) and MOC‐88 (1 μM) generates •OH radical in PBS under aerobic condition. Phosphorescence spectra of RuL^oz^_3_(BF_4_)_2_ (8 μM) under aerobic c) and nitrogen d) environments. Phosphorescence spectra of MOC‐88 (1 μM) under aerobic e) and nitrogen f) environments. Using HPF (10 μM) as fluorescence probe and 450–460 nm laser (4 mW cm^−2^) for irradiation.

The generation of singlet ^1^O_2_ upon light irradiation of RuL^oz^_3_(BF_4_)_2_ and MOC‐88 was proved by electron paramagnetic resonance (EPR) tests (Figure S19, Supporting Information). The characteristic peaks with 1:1:1 intensity were observed, ascribed to the resonance of the adduct of ^1^O_2_ with 2,2,6,6‐tetramethyl‐4‐piperidone hydrochloride. The singlet ^1^O_2_ quantum yields of RuL^oz^_3_(BF_4_)_2_ and MOC‐88 were evaluated using the capture agent 9,10‐anthracenediyl‐bis(methylene) dimalonic acid (ABDA).^[^ ^38^ ^]^ Notably, upon irradiation with 450–460 nm laser, the reduction of ABDA absorbance in the presence of MOC‐88 is more significant in comparison with that of RuL^oz^_3_(BF_4_)_2_, indicating that MOC‐88 is more efficient than RuL^oz^_3_(BF_4_)_2_ in producing ^1^O_2_ under the aerobic conditions (Figure S20, Supporting Information). The ^1^O_2_ quantum yields of MOC‐88 and RuL^oz^_3_(BF_4_)_2_ were calculated to be 0.63 and 0.46 (**Figure** **2**a), respectively. This confirms that the integration of eight RuL^oz^_3_(BF_4_)_2_ into a single‐MOC‐88 cage, coupled by the heavy atom effect introduced by six Pd atoms, can effectively enhance the energy transfer efficiency from the ^3^MLCT states of the RuL^oz^_3_‐based PSs to the oxygen molecules to generate more singlet ^1^O_2_.

Meanwhile, MOC‐88 exhibits a superior ability to generate •OH compared to RuL^oz^_3_(BF_4_)_2_ (Figure 2b–f and Figure S21–S23, Supporting Information), showing a rate increased by 6.59 times under aerobic condition, as detected by hydroxyphenyl fluorescein (HPF) as an •OH probe, which can emit strong green fluorescence at 515 nm after reacting with •OH. Furthermore, EPR spectroscopy using 5‐tert‐butoxycarbonyl‐5‐methyl‐1‐pyrroline‐N‐oxide (BMPO) as a spin trap showed that pure BMPO solution was silent under irradiation of 450–460 nm (Figure S21, Supporting Information). While in the presence of RuL^oz^_3_(BF_4_)_2_ and MOC‐88, a series of characteristic EPR signals of •OH radical with peak intensity of 1:2:2:1 was observed. The EPR signal intensity of MOC‐88 is significantly higher than that of RuL^oz^_3_(BF_4_)_2_, which suggests that the cage has better performance to promote •OH generation. Strikingly, MOC‐88 is able to generate •OH even under nitrogen atmosphere, while RuL^oz^_3_(BF_4_)_2_ cannot under such anaerobic condition (Figure 2d,f). As shown in Figure S24, Supporting Information, we found that hydroxyl radicals can still be generated under the condition of pH 6, so acidic TME has no effect on the generation of OH^−^. This finding indicates that MOC‐88 can produce •OH without the involvement of O_2_ molecule, exhibiting potential photosensitizing property for O_2_‐independent Type‐I PDT.

**Figure 3:**
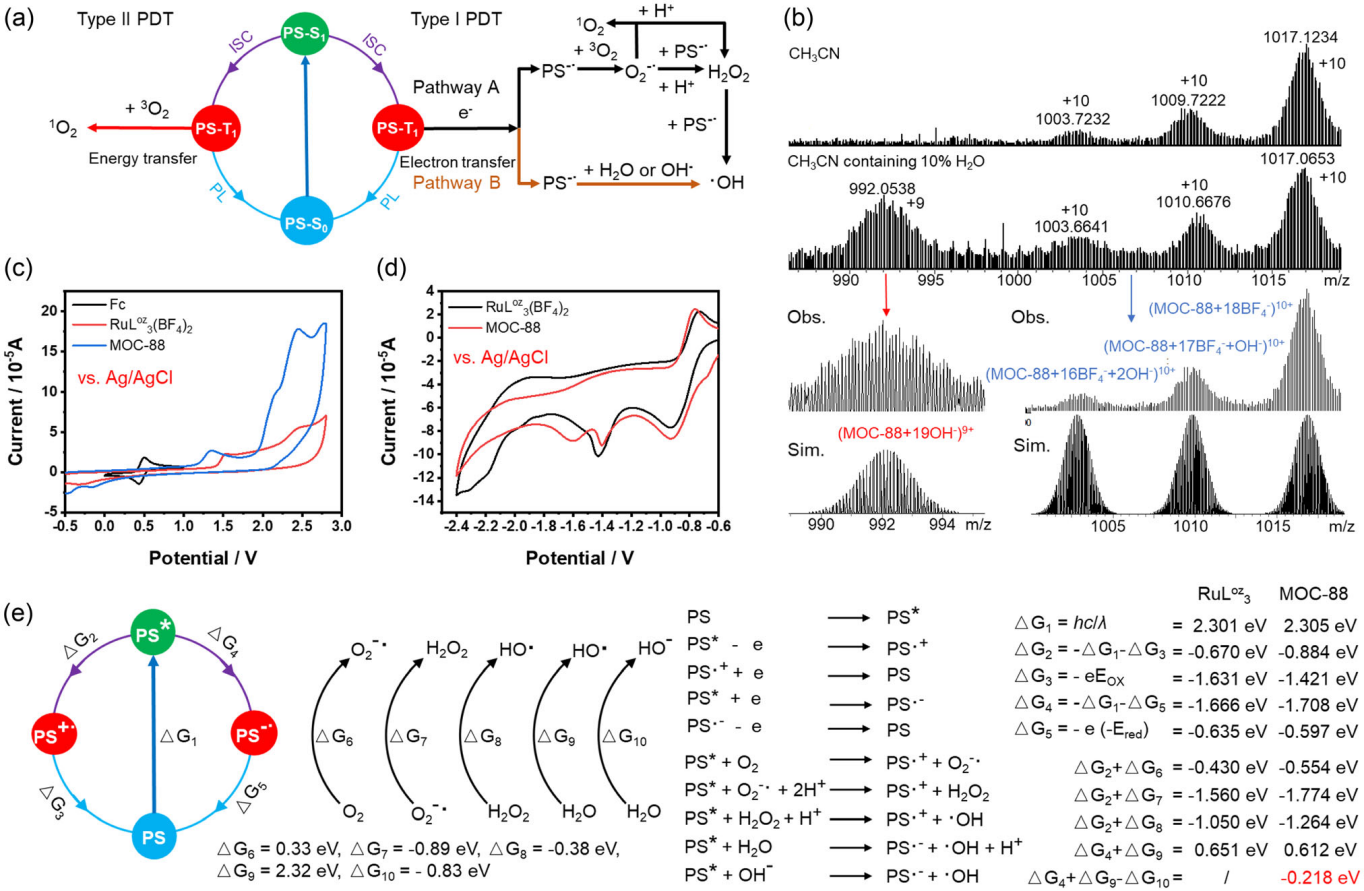
a) Mechanism diagram of various ROS generation by photoexcitation of RuL^oz^_3_(BF_4_)_2_ and MOC‐88 PS. b) HR‐ESI‐TOF‐MS spectra of MOC‐88 in pure CH_3_CN and a CH_3_CN solution containing 10% H_2_O. c,d) Cyclic voltammograms of RuL^oz^_3_(BF_4_)_2_ and MOC‐88 with Fc as calibration in CH_3_CN using 0.01 m TBAPF_6_ as the electrolyte at room temperature. Data were collected using the standard three electrode setup with a glassy carbon working electrode, platinum wire counter electrode and Ag/AgCl reference electrode. e) The proposed mechanisms and Gibbs free energy of possible processes in Type‐I PDT photoinduced by RuL^oz^_3_(BF_4_)_2_ or MOC‐88 PS.

Then, we explored the O_2_‐dependence mechanism of Type‐I photochemical process based on our experimental and literature results (**Figure** **3**a).^[^ ^39^ ^]^ From the intersection of normalized absorption and emission spectra of RuL^oz^_3_(BF_4_)_2_ or MOC‐88, their photoexcitation energy of PS→PS* (ΔG_1_) was estimated to be 2.301 or 2.305 eV, respectively. Their first oxidation potential of PS^•+^/PS (ΔG_3_) (Figure 3c) and reduction potential of PS^•−^/PS (ΔG_5_) (Figure 3d) were determined by cyclic voltammetry (CV), giving oxidation/reduction couples of RuL^oz^_3_(BF_4_)_2_ and MOC‐88 at +1.001/−1.265 V and +0.791/−1.227 V (versus Fc^0^/Fc^+^, Fc = ferrocene), respectively, corresponding to +1.631/−0.635 V and +1.421/−0.597 V versus NHE. Therefore, ΔG_2_ (PS*/PS^•+^) and ΔG_4_ (PS*/PS^•−^) of RuL^oz^_3_(BF_4_)_2_ and MOC‐88 could be obtained as shown in Figure 3e. The energy values for electron reduction of O_2_ to yield O_2_^−•^ (ΔG_6_), H_2_O_2_ (ΔG_7_), and •OH (ΔG_8_), and oxidation of water to yield •OH (ΔG_9_) are available from literature.^[^ ^39^, ^40^ ^]^

When O_2_ was participated in Type‐I PDT, the pathway A would take place to produce H_2_O_2_ through electron transfer, which was further reduced to •OH. The calculation results suggest that the Gibbs free energies of •OH production from O_2_ are all negative for RuL^oz^_3_(BF_4_)_2_ and MOC‐88 (Figure 3e), indicating that both RuL^oz^_3_(BF_4_)_2_ and MOC‐88 can photoinduce hydroxyl radical through pathway A under aerobic conditions. However, when O_2_ did not participate in Type‐I PDT process, a pathway B may occur to generate •OH radical from water or OH^−^ photooxidation under the anaerobic condition. The calculated results show that the Gibbs free energies of direct water oxidation to •OH (ΔG_2_ + ΔG_9_) are 0.651 and 0.612 eV for RuL^oz^_3_(BF_4_)_2_ and MOC‐88 (Figure 3e), respectively, proving that this process is thermodynamically implausible from the energy consideration. Instead, a process from photooxidation of OH^−^ anion may be more feasible, which needs an essential prerequisite of OH^−^ generation first. To understand why only MOC‐88 but not RuL^oz^_3_(BF_4_)_2_ can photoinduce •OH radical under N_2_ environment without O_2_ involvement, we carefully examined the solution species revealed by mass spectra of MOC‐88 (Figure 3b). It is evident that, besides the main peak of +10 charge state of (MOC‐88 + 18BF_4_^−^)^10+^, there are two small peaks attributable to (MOC‐88 + 16BF_4_^−^ + 2OH^−^)^10+^ and (MOC‐88 + 17BF_4_^−^ + OH^−^)^10+^ species. Additionally, when adding 10% water into the CH_3_CN solution, a mass peak of +9 charge state assignable to a (MOC‐88 + 19OH^−^)^9+^ species appeared. Furthermore, as shown in Figure S24, Supporting Information, with the increase of temperature, MOC‐88 can accelerate the deuterization rate of phenylacetylene, while RuL^oz^_3_(BF_4_)_2_ and the control cannot achieve this. This reveals the weak acidity enhancement of phenylacetylene after MOC‐88 encapsulation, which is attributed to the electrostatic interaction between the alkaline MOC‐88 cavity and the acetylene transition state. These observations disclose that the highly charged +28 positive MOC‐88 cage has a strong ionization ability to drive water dissociation, generating and stabilizing OH^−^ anions in its positively cage‐confined microenvironment.^[^ ^41^ ^]^ Therefore, photooxidization of OH^−^ to •OH by MOC‐88 becomes thermodynamically plausible with a Gibbs free energy calculated to be −0.218 eV, which ultimately leads to an accelerated transformation of water into •OH radicals. This validates a pathway B for the Type‐I PDT process as verified by the HPF experiment under N_2_ environment, confirming that MOC‐88 can not only improve the singlet ^1^O_2_ quantum yield for Type‐II PDT and •OH production under aerobic condition for Type‐I PDT, but also can generate •OH radicals in the absence of O_2_ molecules by ionizing and photooxidizing water for an O_2_‐independent Type‐I PDT process under anaerobic condition.

All in all, in a nitrogen environment, MOC‐88 can react through the pathway B in Figure 3a, which first ionizes water molecules to release hydroxide ions under the influence of the high positive charge of the cage structure. Subsequently, under the excitation of light, hydroxide ions are oxidized to hydroxyl radicals by the excited state of MOC‐88. In contrast, RuL^oz^_3_(BF_4_)_2_, lacking a high positive charge structure, cannot ionize hydroxide ions and thus unable to carry out this pathway. The occurrence of this Type‐I PDT process does not depend on the presence of O_2_; it only occurs in the presence of a certain amount of water. In a normoxic environment, both MOC‐88 and RuL^oz^_3_(BF_4_)_2_ can undergo Type‐II PDT and the traditional low O_2_‐dependent Type‐I PDT (pathway A).

### 2.2 Cellular Uptake and In vitro PDT Effect of MOC‐88

**Figure 4:**
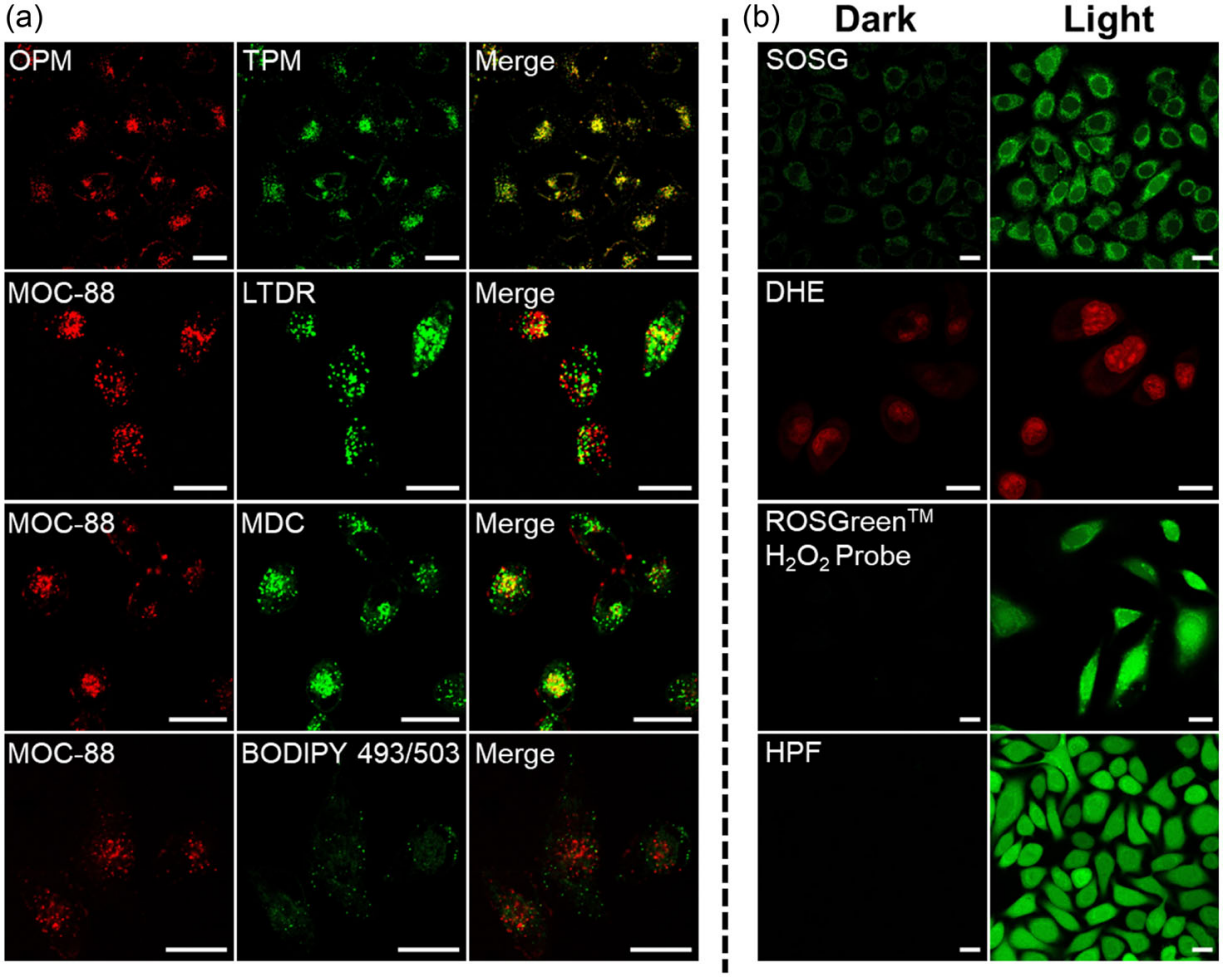
a) One‐ and two‐photon imaging of Hela cells treated with MOC‐88, and co‐localization of MOC‐88 with LTDR (targeted lysosome), MDC (targeted autolysosome) and BODIPY 493/503 (targeted lipid droplet) in Hela cells. MOC‐88: *λ*_ex_ = 488 nm (OPM) or 810 nm (TPM), *λ*_em_ = 625 ± 25 nm; LTDR: *λ*_ex_ = 633 nm, *λ*_em_ = 678 ± 20 nm; MDC: *λ*_ex_ = 405 nm, *λ*_em_ = 523 ± 20 nm; BODIPY 493/503: *λ*_ex_ = 488 nm, *λ*_em_ = 513 ± 20 nm. b) CLSM detection of intracellular SOSG (^1^O_2_), DHE (O_2_^−^), ROSGreen^TM^ H_2_O_2_ Probe (H_2_O_2_), HPF (•OH) production in Hela cells after MOC‐88 (1.0 μM) treatment with and without light (450–460 nm, 20 mW cm^−2^). SOSG, HPF and ROSGreen H_2_O_2_ Probe: *λ*_ex_ = 488 nm, *λ*_em_ = 520 ± 20 nm. DHE: *λ*_ex_ = 514 nm, *λ*_em_ = 610 ± 20 nm. Scale bar: 20 μm.

The Hela cellular uptake have been investigated by measuring Ru‐element content with inductively coupled plasma MS (ICP–MS, Figure S25, Supporting Information) and Ru‐based emission with confocal laser scanning microscopy (CLSM, Figure S26–29, Supporting Information), revealing a time‐ and concentration‐dependent uptake behavior of MOC‐88 as the intracellular red phosphorescence gradually enhanced along the increase of incubation time and concentration. The phosphorescence intensity showed little decrease in 24 h after treatment with MOC‐88, suggesting that MOC‐88 can be quickly internalized into cancer cells and stay inside the cells without being excreted (Figure S27, Supporting Information). When incubating the cells at a low temperature (4 °C), the intracellular phosphorescence was marginal, but significantly enhanced when incubated at 37 °C, proving that the internalization of MOC‐88 was through an energy‐dependent endocytosis mechanism (Figure S28, Supporting Information). Owing to the unique property of MOC‐88 that can be excited by both one and two photons, the convenient one‐ and two‐photon microscope (OPM and TPM) could be applied for co‐localization imaging, covering a wide range of light wavelengths (**Figure** **4**a and Figure S29, Supporting Information), which indicated that the cage mainly entered the lysosome and autolysosome, exhibiting a point distribution fashion within the cells.

The efficient cellular uptake and lysosome localization of MOC‐88 encouraged us to evaluate its PDT effect. We assessed the (photo)cytotoxicity of RuL^oz^_3_(BF_4_)_2_ and MOC‐88 on Hela and U14 cell lines by 3‐(4,5‐dimethylthiazol‐2‐yl)2,5‐diphenyl tetrazolium bromide (MTT) assay (**Table** **1** and Table S3, Supporting Information). The cell viabilities were tested and compared under normoxia and hypoxia (1% O_2_) by treatment of RuL^oz^_3_(BF_4_)_2_ and MOC‐88 and irradiation with 450–460 nm light‐emitting diode (LED) (12 J cm^−2^) or not. In dark condition, the IC_50_ values of RuL^oz^_3_(BF_4_)_2_ were much lower than those of MOC‐88 when equivalent Ru‐photosensitive centers are counted, suggesting a significant decrease of the dark toxicity of MOC‐88 compared to RuL^oz^_3_(BF_4_)_2_. On the contrary, the cell viability dramatically decreased when cells were incubated with the complexes under light irradiation, reflecting by obvious decrease of the IC_50_ values in light conditions. Noticeably, the IC_50_ values of MOC‐88 were reduced much more than those of RuL^oz^_3_(BF_4_)_2_. As the cell viability in dark and light correlates to a phototoxicity index (PI), we can see, MOC‐88 showed much improved PIs than RuL^oz^_3_(BF_4_)_2_ not only under normoxia but also under hypoxia, demonstrating higher phototoxicity than RuL^oz^_3_(BF_4_)_2_ to lead to better treatment outcomes. It is also noticeable that, under hypoxia (1% O_2_), the PIs of RuL^oz^_3_(BF_4_)_2_ were almost unchanged, while those of MOC‐88 were approximately twice enhanced compared with RuL^oz^_3_(BF_4_)_2_, which confirmed an effective induction effect of MOC‐88 for O_2_‐independent Type‐I PDT. These results suggest that MOC‐88 is competent for photocytotoxicity in vitro both under normoxia and hypoxia, and applicable for different cancer cell lines. Moreover, we tested the toxicity of MOC‐88 on normal cell lines HLF‐a and 3T3‐L1. Due to the fact that the compound we synthesized does not have tumor cell targeting properties, it also has certain toxicity to normal cell lines. However, as shown in Table 1 and Table S3, Supporting Information, the sensitivity of different cell lines to it also varies, and its toxicity is lower than tumor cell lines Hela and U14.

**Table 1:** Cytotoxicity (IC_50_, μM) of tested complexes against cancerous cell lines for 24 h in the absence and presence of 450–460 nm irradiation (20 mW cm^−2^, 10 min) upon treatment with complexes for 3 h (mean ± SD, *n* = 3).

Compound	Dark [μM]	Light [μM]	PIa)	Dark [μM]	Light [μM]	PIa)
Hela (Normoxia)				Hela (Hypoxia)		
RuL^oz^_3_(BF_4_)_2_	45.24 ± 1.44	20.93 ± 0.36	2.16	49.44 ± 2.37	48.15 ± 3.88	1.03
MOC‐88b)	117.04 ± 5.12	7.36 ± 0.80	15.90	102.88 ± 4.80	35.23 ± 1.57	2.92
U14 (Normoxia)				U14 (Hypoxia)		
RuL^oz^_3_(BF_4_)_2_	52.11 ± 0.79	17.97 ± 0.46	2.90	56.93 ± 2.06	47.10 ± 3.36	1.21
MOC‐88b)	76.32 ± 4.72	13.53 ± 0.24	5.64	74.76 ± 0.72	39.12 ± 0.32	1.94

a) Phototoxicity index (PI) is defined as the ratio of ^dark^IC_50_/^light^IC_50_.

b) Equivalent concentration of Ru‐photocenter.

To verify the capability of MOC‐88 for both Type‐I and Type‐II PDT in living cells, CLSM was performed using a series of commercial indicators (Figure 4b and Figure S30–S33, Supporting Information), including the total ROS indicator 2,7‐dichlorofluorescein diacetate, ^1^O_2_ indicator singlet oxygen sensor green (SOSG), O_2_^−•^ indicator dihydroethidium (DHE), H_2_O_2_ indicator ROSGreen (H_2_O_2_ probe), and •OH indicator HPF. After staining Hela cells treated by MOC‐88, the indicators’ fluorescence was negligible in the dark, but substantially increased upon irradiation (Figure 4b and Figure S33, Supporting Information). The photoinduced concentration‐dependent intracellular ROS production and the intracellular lipid ROS production in Hela cells were also testified by flow cytometry and CLSM (Figure S30–S34, Supporting Information). Moreover, the ROS increase along TPA stimulation of MOC‐88 was validated by low‐energy light excitation at 810 nm (Figure S32, Supporting Information). The previous results clearly demonstrate that MOC‐88 could simultaneously induce the processes of Type‐I and Type‐II PDT in vitro, exhibiting both one and two‐photon characteristics as an efficient PDT PS.

### 2.3 MOC‐88 Photoinduced Ferroptosis and PANoptosis In vitro

**Figure 5:**
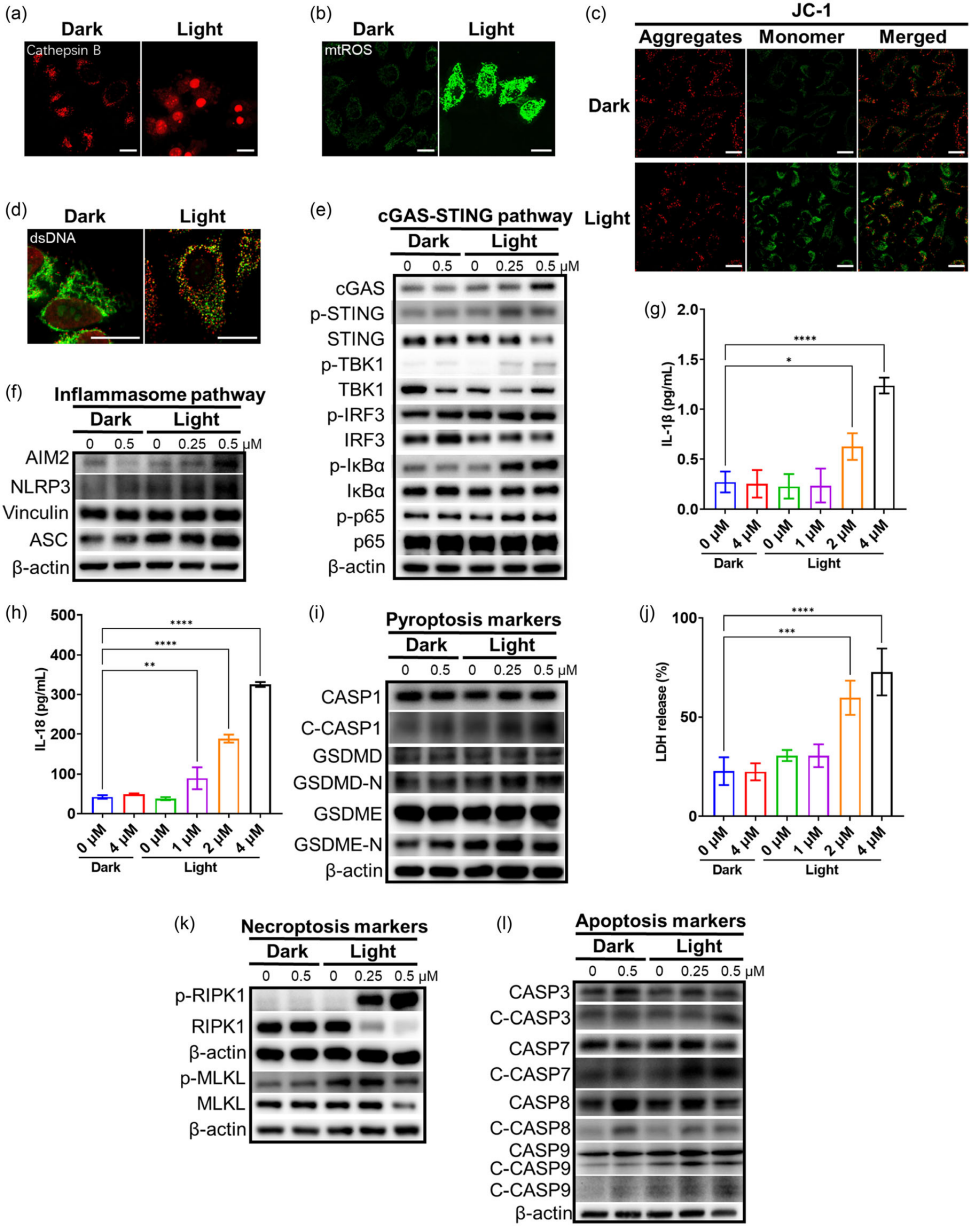
CLSM detection of a) Cathepsin B release, b) intracellular mtROS production, c) MMP effect measured by JC‐1 staining and d) dsDNA leakage of Hela cells after MOC‐88 treatment with and without light (450–460 nm, 20 mW cm^−2^). Scale bar: 20 μm. Western blot analysis of protein expression in the e) cGAS‐STING pathway and f) inflammasome pathway in Hela cells after MOC‐88 treatment with and without light (450–460 nm, 20 mW cm^−2^). g) IL‐1*β* secretion (mean ± SD, *n* = 3) and h) IL‐18 secretion (mean ± SD, *n* = 3) into the supernatant measured by ELISA (enzyme linked immunosorbent assay) in Hela cells after MOC‐88 treatment with and without light (450–460 nm, 20 mW cm^−2^). i) Western blot analysis of protein expression in the pyroptosis in Hela cells after MOC‐88 treatment with and without light (450–460 nm, 20 mW cm^−2^). j) Lactate dehydrogenase (mean ± SD, *n* = 3) release from Hela cells after MOC‐88 treatment with and without light (450–460 nm, 20 mW cm^−2^). Western blot analysis of protein expression in the k) necroptosis and l) apoptosis in Hela cells after MOC‐88 treatment with and without light (450–460 nm, 20 mW cm^−2^). Statistical *P*‐value: *P < 0.05, **P < 0.01, ***P < 0.001, ****P < 0.0001, by one‐way ANOVA.

The real‐time CLSM has been carried out to monitor subcellular organelle change during the photodynamic process (Figure S35, Supporting Information). Since the cage entered the cell and located in the lysosome, the ROS generated by light excitation should destroy the lysosome first. As monitored by Lyso‐Tracker Deep Red (LTDR), the number of bright green spots in LTDR gradually decreased, indicative of lysosome damage. Meanwhile, as seen from the changes of MOC‐88 phosphorescence and Magic Red Substrate (MR‐(RR)_2_) as cathepsin B indicator, the cage and cathepsin B were released from the lysosome and diffused to the whole cytoplasm, some entering the nucleus while some expelled from the cell (**Figure** **5**a and S36, Supporting Information). The photodynamic process also made the mitochondrial ROS increase and caused mitochondrial damage (Figure 5b and S37a, Supporting Information). The real‐time confocal microscopic imaging unveiled that the morphology of mitochondria was changing from a normal reticular structure to a dot‐like distribution. The mitochondrial membrane potential (MMP) was measured by 5,5′,6,6′‐tetrachloro‐1,1′,3,3′‐tetraethyl‐imidacarbocyanine (JC‐1) assay, which showed that MOC‐88 caused a concentration‐dependent increase in proportions of cells with decreased MMP (Figure 5c and Figure S38–S39, Supporting Information). Therefore, the mitochondrial oxidative damage promoted cytoplasmic leakage of its own mtDNA (Figure 5d and Figure S37b, Supporting Information), thus activating the cGAS–STING pathway (Figure 5e, Figure S40a and Table S4, Supporting Information), leading to phosphorylation cascades of STINGs, TBK1, and IRF3, thereby inducing downstream NF‐κB pathway (Figure S40b, Supporting Information) and NOD‐like receptor thermal‐protein‐domain‐associated protein 3 (NLRP3) inflammasomes (Figure 5f and Table S4, Supporting Information). It is noteworthy that the released cathepsins could also promote the formation of NLRP3 inflammasomes.^[^ ^42^ ^]^

As a cytosDNA, absent in melanoma 2 (AIM2) could form AIM2 inflammasomes (Figure 5f and Table S4, Supporting Information) with apoptosis‐associated speck‐like protein containing a CARD (ASC). These inflammasomes further resulted in activation of interleukin (IL)‐1*β* (Figure 5g) and IL‐18 (Figure 5h) mediated by caspase‐1, as well as cleavage of gasdermin D (GSDMD), leading to pyroptosis (Figure 5i and Table S4, Supporting Information). By monitoring the changes of cell‐membrane‐targeted probes 1,1′‐dioctadecyl‐3,3,3′,3′‐tetramethylindocarbocyanine perchlorate (Dil) in real time (Figure S35, Supporting Information) and detecting the release of intracellular lactate dehydrogenase (Figure 5j), we can see that the cell membrane has been damaged. Since PANoptosis regulated by PANoptosomes has key characteristics of cell pyroptosis, apoptosis, and necroptosis, we detected the expression of constituent proteins of PANoptosomes using western blot (WB). As depicted in Figure 5e,f,i,k–l and Table S4, Supporting Information, the expressions of the constituent NLRP3, AIM2, ASC, Caspase 1, Caspase 8, and receptor‐interacting protein kinase 1 (RIPK1) proteins of PANoptosomes, and the downstream indicators of apoptosis, pyroptosis, and necroptosis, were all significantly increased after treatment with MOC‐88 in different concentrations when compared with the control. The key molecular events of necroptosis are the phosphorylation of mixed lineage kinase domain‐like pseudokinase (MLKL) and the activation of GSDMD‐N and GSDME‐N, which translocate to the cell membrane, causing membrane damage and ultimately cell death. Through WB experiments in Figure 5k, we found that the content of p‐MLKL gradually increases during this process, indicating the presence of necroptosis. These pieces of evidence manifest that MOC‐88 could trigger the inflammatory PCD of PANoptosis by its efficient PDT effect (Scheme 1).

**Figure 6:**
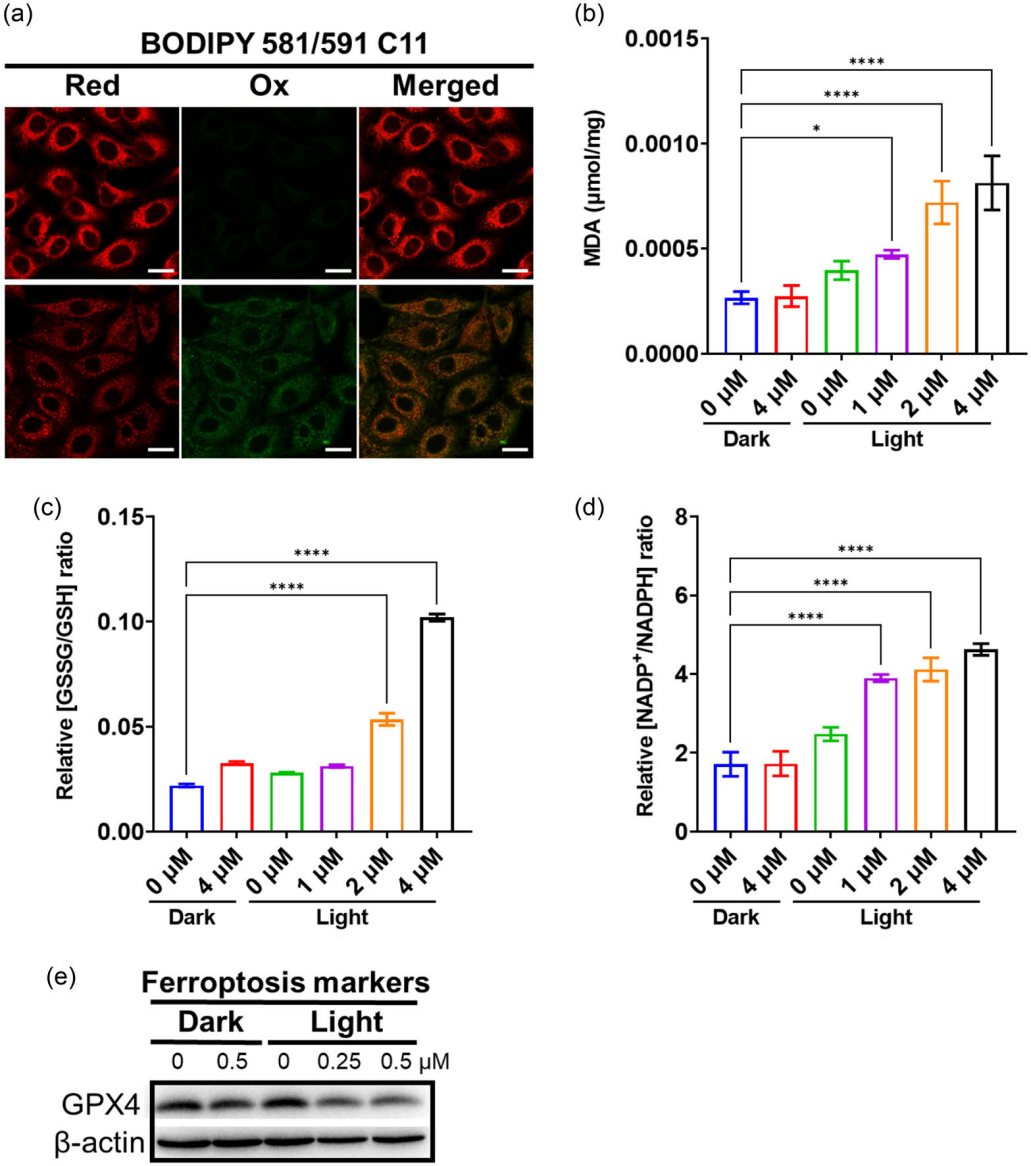
a) CLSM detection of intracellular Lipid ROS production after MOC‐88 treatment with and without light (450–460 nm, 20 mW cm^−2^). Scale bar: 20 μm. b) Intracellular lipid peroxidation level (mean ± SD, *n* = 3) measured after MOC‐88 treatment with and without light (450–460 nm, 20 mW cm^−2^). Impact of MOC‐88 on the ratios of cellular c) GSSG/GSH (mean ± SD, *n* = 4) and d) NADP^+^/NADPH (mean ± SD, *n* = 4) after MOC‐88 treatment with and without light (450–460 nm, 20 mW cm^−2^). GSH: glutathione; GSSG: oxidized glutathione; NADPH: reduced nicotinamide adenine dinucleotide phosphate; NADP^+^: oxidized nicotinamide adenine dinucleotide phosphate. e) Western blot analysis of GPX4 expression in Hela cells after MOC‐88 treatment with and without light (450–460 nm, 20 mW cm^−2^). Statistical *P*‐value: *P < 0.05, **P < 0.01, ***P < 0.001, ****P < 0.0001, by one‐way ANOVA.

Recently, it has been reported that there is a certain relationship between ferroptosis and PANoptosis.^[21a,21b]^ The alternations in cell morphology upon MOC‐88 treatment were detected by transmission electron microscope (Figure S41, Supporting Information). Compared with the control, the treated cells showed typical ultrastructural characteristics of ferroptosis, e.g., shrinkage of mitochondria with increased density of bilayer membrane, while the morphology of nucleus was unaffected. Since GSH not only protects against cellular oxidative damage, but also acts as the reducing substrate of GPX4, so depletion of GSH can result in ferroptosis. The reduced nicotinamide adenine dinucleotide phosphate (NADPH) is important for the nourishing of the GSH‐ and thioredoxin‐dependent systems, as well as inhibiting the LPO; therefore, reduction of NADPH is also a key feature and inducement of ferroptosis. The intracellular LPO was verified by evaluating LPO level via lipid oxidation (BODIPY 581/591 C11), and by measuring the intracellular concentration of malondialdehyde (MDA). After MOC‐88 and irradiation treatment, a significant concentration‐dependent increase was observed for the green/red fluorescence intensity ratio of BODIPY 581/591 C11 (**Figure** **6**a and Figure S34, Supporting Information), the MDA level (Figure 6b), and the ratio of oxidized GSH (GSSG)/GSH (Figure 6c) and oxidized nicotinamide adenine dinucleotide phosphate (NADP^+^)/NADPH (Figure 6d). Compared with the control, the expression of GPX4 in cells treated with different concentrations of MOC‐88 decreased significantly (Figure 6e and Table S4, Supporting Information). These results suggest that MOC‐88 could also induce ferroptosis of Hela cells. In sum, MOC‐88 is proven to be an effective PDT PS to promote ROS generation, and subsequently activate inflammasome, cGAS–STING pathway, NF‐κB pathway, as well as ferroptosis and PANoptosis to evoke tumor cell death.

In summary, excessive ROS production, denoted as “oxidative distress”, can induce cytotoxicity and trigger multiple forms of cell death. On the one hand, under the stimulation of ROS, a series of caspases are activated, leading to apoptosis of tumor cells. The activation of caspase‐8 triggers the activation of p‐MLKL, leading to necroptosis of tumor cells. In addition, ROS also disrupts the integrity of lysosomes, leading to the release of tissue proteases, thereby activating the downstream NLRP3 inflammasome pathway. Meanwhile, ROS also disrupts the integrity of mitochondria, releasing dsDNA, further activating the cGAS–STING pathway and AIM2 inflammasome pathway. It is worth noting that the activation of caspase‐3 leads to the GSDME‐dependent pyroptosis pathway. The synergistic effect of these pathways jointly promotes pyroptosis in tumor cells. Therefore, it can also be summarized as PANoptosis. On the other hand, under the stimulation of abundant hydroxyl radicals, they will react with unsaturated fatty acids in the plasma membrane to generate lipid peroxides, ultimately to evoke ferroptosis.

### 2.4 MOC‐88 Photoinduced Immunogenic Cell Death In vitro and Cancer Immune Response In vivo

**Figure 7:**
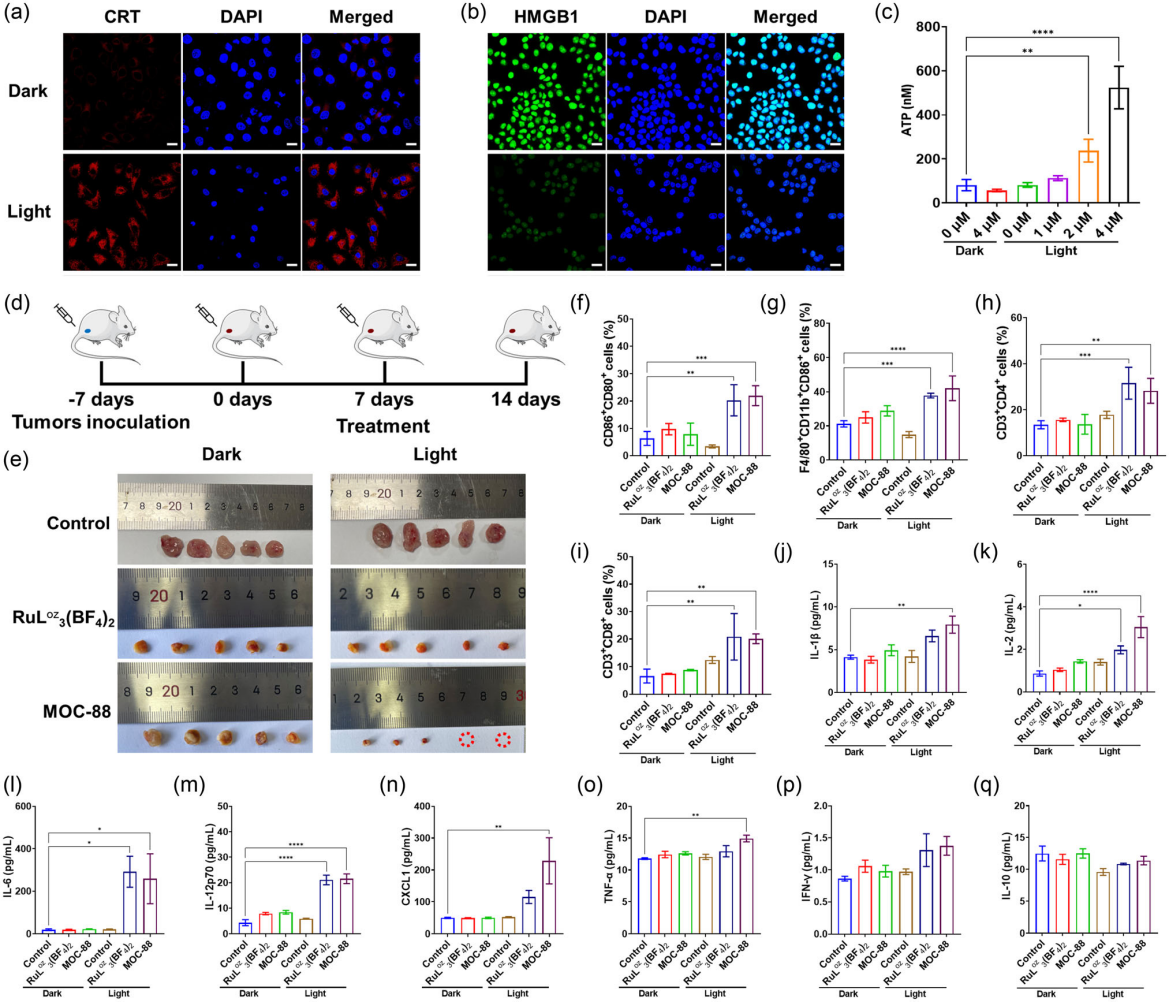
CLSM images of a) ecto‐CRT and b) HMGB1 after MOC‐88 treatment with and without light (450–460 nm, 20 mW cm^−2^). Scale bar: 20 μm. c) ATP levels (mean ± SD, *n* = 3) into the supernatant in Hela cells after treatment with MOC‐88 and light (450–460 nm, 20 mW cm^−2^). d) Schematic illustration of in vivo therapeutic protocol. e) The photographs of the tumor collected at the end of therapeutic period. f) Expression of CD80 and CD86 quantitatively after treatment detected by flow cytometry in vivo (mean ± SD, *n* = 3). g) The polarization of macrophage (mean ± SD, *n* = 3), populations of h) CD4^+^ (mean ± SD, *n* = 3) and i) CD8^+^ T cells (mean ± SD, *n* = 3) after treatment in vivo by flow cytometry analysis. Secretion of j) interleukin‐1*β* (IL‐1*β*), k) interleukin‐2 (IL‐2), l) interleukin‐6 (IL‐6), m) interleukin‐12p70 (IL‐12p70), n) chemokine (CXCL1), o) tumor necrosis factor *α* (TNF‐*α*), p) Interferon *γ* (IFN‐*γ*), and (q) interleukin‐10 (IL‐10) in sera measured after treatment in vivo (mean ± SD, *n* = 4). Statistical *P*‐value: *P < 0.05, **P < 0.01, ***P < 0.001, ****P < 0.0001, by one‐way ANOVA.

Cancer cells undergoing immunogenic cell death (ICD) present damage‐associated molecular pattern, including calreticulin (CRT), high‐mobility group box 1 (HMGB1), and adenosine triphosphate (ATP).^[^ ^43^ ^]^ As shown in immunofluorescence assay (**Figure** **7**a), the red emission from ecto‐CRT in MOC‐88 treated cells was negligible in the dark, but significantly enhanced upon irradiation, indicating the high expression of ecto‐CRT photoinduced by MOC‐88. Meanwhile, green fluorescence from HMGB1 in MOC‐88‐treated cells decreased from the nucleus upon irradiation (Figure 7b). Similar changes of CRT and HMGB1 were also obtained by flow cytometry (Figure S42, Supporting Information). In addition, MOC‐88 induced the extracellular secretion of ATP in a concentration‐dependent manner, as indicated by the substantially increased ATP concentration in the supernatant upon irradiation (Figure 7c). All these results revealed that the cells photoinduced by MOC‐88 had high immunogenicity, showing a potential to activate antitumor immunity.

Using female BALB/c mice carrying U14 tumors, we evaluated the efficacy of the two‐photon PDT mediated by MOC‐88 in vivo (Figure 7d). The mice were randomly divided into six groups, namely, control+dark, RuL^oz^_3_(BF_4_)_2_+dark, MOC‐88+dark, control+light, RuL^oz^_3_(BF_4_)_2_+light, and MOC‐88+light. When the tumor volume reached about 70 mm^3^, the drugs were injected into the tumor and irradiated with 810 nm light. Compared with the control group, the tumor growth was significantly inhibited in other mice groups (Figure 7e and Figure S43–S45, Supporting Information). Specifically, in the dark groups, the tumors decreased by ≈85.13% in response to RuL^oz^_3_(BF_4_)_2_ and 73.01% in response to MOC‐88. Similarly, in the light groups, the tumors also showed a reduction of about 85.13% for RuL^oz^_3_(BF_4_)_2_ and 73.01% for MOC‐88. The tumor growth inhibition in dark was attributed to the intrinsic chemotherapy of RuL^oz^_3_(BF_4_)_2_ and MOC‐88, with RuL^oz^_3_(BF_4_)_2_ displaying better antitumor effect than MOC‐88, in consistent with the lower dark toxicity of MOC‐88 than RuL^oz^_3_(BF_4_)_2_ as discussed earlier. However, after irradiation, MOC‐88 exhibited much enhanced antitumor effect than RuL^oz^_3_(BF_4_)_2_, indicating superior performance of MOC‐88 for two‐photon PDT. At the end of treatment, no significant structural or pathological changes were observed in the hematoxylin and eosin staining of the main organs (Figure S46, Supporting Information).

We then analyzed immune cells in tumors, adjacent tissues, and tumor‐draining lymph nodes using flow cytometry. Under light conditions, both RuL^oz^_3_(BF_4_)_2_ and MOC‐88 caused an ≈1.5‐ to 2‐fold increase in the percentage of CD86^+^CD80^+^ cells (Figure 7f), F4/80^+^CD11b^+^CD86^+^ cells (Figure 7g), CD3^+^CD4^+^ T cells termed as helper T cell (Figure 7h), and CD3^+^CD8^+^ T cells termed as cytotoxic T lymphocytes (Figure 7i). But the percentage of CD3^+^CD4^+^FOXP3^+^ T cells termed as immunosuppressive regulatory T cells (Tregs, Figure S47, Supporting Information) decreased by 2‐2.5 times. While under dark conditions, the proportion of these cells did not show prominent difference from the control group.

The secretion of cytokines also plays an important role in activating antitumor immune responses. As seen from Figure 7k–q, compared with control group, significant increase in tumor necrosis factor *α* (TNF‐*α*), IL (IL‐1*β*, IL‐2, IL‐6, and IL‐12p70), IFN‐*γ*, and chemokines (CXCL1) was detected in the serum samples of the treated groups by RuL^oz^_3_(BF_4_)_2_ and MOC‐88 after light stimulation, while a slight decrease in anti‐inflammatory cytokines (IL‐10) was observed. These findings further proved that RuL^oz^_3_(BF_4_)_2_ and MOC‐88 could activate strong immune responses. It is worthy of mentioning that the photoinduced increase in the proportion of CD86^+^CD80^+^ cells can be considered as hallmarks for dendritic cells (DCs)’ maturation,^[22]^ which will promote T cells recruitment into the tumor and activate T cells.^[^ ^44^ ^]^ At the same time, we observed an increase in the proportion of CD3^+^CD4^+^ T cells and CD3^+^CD8^+^ T cells, indicative of tumor immunotherapy mediated by CD3^+^CD8^+^ T cells. And less Tregs can further improve the efficacy of cancer immunotherapy. Interestingly, after phototherapy, macrophages polarized into antitumor M1 type (F4/80^+^CD11b^+^CD86^+^ cells), and the concentration of inflammatory cytokines (TNF‐*α* & IL‐1*β*) also increased due to their conversion to M1.^[^ ^45^ ^]^ These in vivo results collectively indicate that MOC‐88 PDT can enhance tumor immunogenicity, trigger T‐cell‐dependent immune responses and, therefore, not only have excellent antitumor effects, but also showcase adaptive immunotherapy performance.

## 3 Conclusion

We have designed a versatile MOC (MOC‐88) with the characteristics of high‐positive charges, good water solubility, and one‐/two‐photon phosphorescence suitable for efficient PDT treatment. Notably, MOC‐88 can not only efficiently promote singlet ^1^O_2_ yield and ROS production in the presence of oxygen to enhance both Type‐I and Type‐II PDT, but also generate hydroxyl radicals in an oxygen free environment to enable an O_2_‐independent Type‐I PDT process. Moreover, MOC‐88 could evoke PANoptosis, ferroptosis, and ICD, which induce tumor immunogenicity. These processes eventually promote the maturation of DCs and activating T‐cell‐dependent adaptive immune responses in mice, thereby eliminating tumors. This work sheds a light on the design of novel PS to overcome cancer treatment difficulty in hypoxia, even anaerobic and non‐immunogenic tumor environments, holding a promise for advancement in photodynamic immunotherapy.

## 4 Experimental Section

4.1

4.1.1

##### Self‐Assembly and Characterization of MOC‐88

Pd(BF_4_)_2_(CH_3_CN)_4_ (106.62 mg, 240 μmol) was dissolved into 5 mL CH_3_CN and then added to a solution of RuL^oz^_3_(BF_4_)_2_ (374.27 mg, 320 μmol, 1 eq.) in CH_3_CN (15 mL). The mixture was heated and stirred at 80 °C for 1 h. After the reaction has completed, the mixture was cooled down and filtered to remove insoluble impurities. The filtrate was poured into 1000 mL ether under stirring to provide large quantity of precipitates. The precipitates were filtered and washed with ether to give MOC‐88 as a red solid. Single crystals suitable for X‐Ray diffraction analysis were obtained by the diffusion of CH_3_OH into 400 μL MOC‐88 CH_3_CN solution (0.2 mM) for ≈3–4 days. The single‐crystal data were deposited in the Cambridge Crystallographic Data Center (CCDC code: 2 280 671).

##### Cell Culture

The cells (Hela, HLF‐a, 3T3‐L1, and U14) were obtained from Experimental Animal Center of Sun Yat‐Sen University, China. Cells were cultured in DMEM medium with 10% FBS, 100 μg mL^−1^ streptomycin, and 100 U mL^−1^ penicillin as supplement. Cells were cultured at 37 °C in a humidified incubator with an atmosphere of 5% CO_2_% and 95% air (normoxia) or 1% oxygen, 5% carbon dioxide, and 94% nitrogen (hypoxia).

##### Ethical Statement for Animal Experiments

All animal operations were in accord with guidelines of Institute of Zoology, Guangdong Academy of Sciences. The accreditation number is GIZ20230327.

##### Animal Models

Pathogen‐free female BALB/c mice, 4–5 weeks of age, were purchased and bred in the Institute of Zoology, Guangdong Academy of Sciences. The BALB/c female mice were randomly divided into six groups (control+dark, RuL^oz^_3_(BF_4_)_2_+dark, MOC‐88+dark, control+light, RuL^oz^_3_(BF_4_)_2_+light, and MOC‐88+light), and each group contained five mice. U14 cells were subcutaneously inoculated into the BALB/c mice. When the primary tumors reach the size of 70 mm^3^, RuL^oz^_3_(BF_4_)_2_ (4.24 mg kg^−1^) and MOC‐88 (5 mg kg^−1^) were intratumorally injected. After 24 h, the laser groups were exposed to an 810 nm two‐photon laser for 10 min.

##### Statistical Analysis

All quantitative data were presented as the mean ± standard deviation (SD). GraphPad Prism for Windows (GraphPad Software, San Diego, California, USA) was used for all statistical analyses. One‐way ANOVA was used to determine statistically significant differences and Dunnett's multiple comparison test was used to determine the significance of the differences. Multiple measurements of quantitative normally distributed data were used for two‐way repeated‐measures ANOVA, and *P* < 0.05 was considered statistically significant (statistical *P*‐value: *P < 0.05, **P < 0.01, ***P < 0.001, and ****P < 0.0001).

## Conflict of Interest

The authors declare no conflict of interest.

## Supporting information

Supplementary Material

## Data Availability

The data that support the findings of this study are available from the corresponding author upon reasonable request.
