# Supplementary material for: A Highly Charged Positive Cage Causes Simultaneous Enhancement of Type‐II and O2‐Independent‐Type‐I Photodynamic Therapy via One‐/Two‐Photon Stimulation and Tumor Immunotherapy via PANoptosis and Ferroptosis
Source: Small Sci. 2024 Jul 4;4(11):2400220. doi: 10.1002/smsc.202400220 (PMC11935160; doi:10.1002/smsc.202400220)
Supplement: Supplementary file 1 — Supplementary Material [file SMSC-4-2400220-s001.pdf]

Supporting Information

A Highly Charged Positive Cage Causes Simultaneous Enhancement of Type-II and O_2-Independent-Type-I PDT via One-/Two-Photon Stimulation and Tumor Immunotherapy via PANoptosis and Ferroptosis

Xiao-Dong Zhang,[1] Hui-Juan Yu,[2] Shao-Qi, Guan,[1] Yu-Lin Lu,[1] Yu Zhang,[1] Yin-Hui Huang,[1] Ya-Ping Wang,[3] Chen-Hui Liu,[1] Zhong-Min Cao,[1] Yu-Han Qin,[1] Mei Pan,[1] Jun Shen,[3] and Cheng-Yong Su[1]

[1]MOE Laboratory of Bioinorganic and Synthetic Chemistry, GBRCE for Functional Molecular Engineering, LIFM, IGCME, School of Chemistry, Sun Yat-Sen University, Guangzhou 510275, China.

[2]Guangdong Key Laboratory of Animal Conservation and Resource Utilization, Guangdong Public Laboratory of Wild Animal Conservation and Utilization, Institute of Zoology, Guangdong Academy of Sciences, Guangzhou 510275, China.

[3] Department of Radiology, Guangdong Provincial Key Laboratory of Malignant Tumor Epigenetics and Gene Regulation, Medical Research Center, Sun Yat-Sen Memorial Hospital, Sun Yat-Sen University, Guangzhou 510030.

*Correspondence: cesscy@mail.sysu.edu.cn

Contents

1. Materials..3
2. General Instruments and methods..3
3. Synthetic route..4
   3.1 Ligand L^{oz} (2-(pyridin-3-yl)oxazolo[4,5-f][1,10]phenanthroline)..4
   3.2 Metalloligand $RuL^{oz}{}_3(BF_4)_2$..6
   3.3 Self-assembly and characterization of MOCs..8
4. X-ray Single Crystal Structural Analysis..9
5. UV-Vis and Fluorescence Spectra of $RuL^{oz}{}_3(BF_4)_2$ and MOC-88..10
6. Measurement of two-photon absorption (TPA) cross-section..12
7. pH Stability..13
8. MOC-88 stability in *vitro*..14
9. ROS detection..15
   9.1 Singlet oxygen ($^1O_2$) detection..15
   9.2 Hydroxyl radical (·OH) detection..16
10. Cyclic Voltammetry..18
11. Determination of the lipophilicity..18
12. In *vitro* experiment..18
   12.1 Cell Culture..18
   12.2 Cell viability assay and In *vitro* cytotoxicity..19
   12.3 Intracellular uptake by ICP-MS assay..19
   12.4 Intracellular uptake and localization measured by Confocal microscopy..20
   12.5 Determination of Intracellular ROS levels in Hela cells..22
   12.6 Characterization of specific ROS in Hela cells..23
   12.7 Real-time monitoring of subcellular organelle changes by PDT treatment..25
   12.8 Detection of cathepsin B release..26
   12.9 Detection of ROS content in mitochondria and mitochondrial dsDNA leakage..26
   12.10 Intracellular lipid peroxidation detection in Hela cells..27
   12.11 Measurement of $NADP^+$/NADPH and GSSG/GSH Ratios..27
   12.12 Mitochondrial Membrane Potential Assays..27
   12.13 p65 and p-IRF3 enters the nucleus..29
   12.14 Transmission Electron Microscope..29
   12.15 Extracellular ATP, Release of LDH and Cytokine measurement..30
   12.16 Immunogenic Cell Death..30
   12.17 Western Blotting..31
13. In *vivo* Antitumor Assay..32
14. Statistical analysis..36

1. Materials

$RuCl_3$, 1,10-phenathrolinedione, 3-pyridinecarboxaldehyde, ammonium acetate, were purchased from Adamas, China. Tetrakis(acetonitrile)palladium(II) tetrafluoroborate were purchased from STREM, USA. 3-(4,5)-dimethylthiahiazo (-z-y1)-3,5-di-phenytetrazoliumromide (MTT), 2',7'-dichlorodihydrofluorescein diacetate (H_2DCFDA), singlet oxygen sensor green (SOSG), dihydroethidium (DHE), 3’-(4-Hydroxyphenyl) fluorescein (HPF), 4-(diphenylamino)phenylboronic acid, 9,10anthracenediyl-bis(methylene) dimalonic acid (ABDA), n-Octanol and dimethyl sulfoxide (DMSO) were purchased from Sigma Aldrich, USA. Fetal bovine serum (FBS), Trypsin, Dulbecco's modified eagle medium (DMEM), streptomycin, Phosphate buffered saline (PBS), LysoTracker Deep Red (LTDR), MitoTracker Deep Red (MTDR), MitoTracker Green (MTG), MitoTracker Orange CMTMRos (MTO) were obtained from ThermoFisher Scientific, USA. 2-(4-Amidinophenyl)-6-indolecarbamidine dihydrochloride (DAPI), Hoechst 33342, 1,1'-dioctadecyl-3,3,3',3'-tetramethylindocarbocyanine perchlorate (Dil), 5,5′,6,6′-Tetrachloro1,1′,3,3′-tetraethyl-imidacarbocyanine iodide (JC-1), Autophagy Staining Assay Kit with MDC, Lipid Peroxidation MDA Assay Kit, Enhanced ATP Assay Kit, GSH and GSSG Assay Kit, $NADP^+$/NADPH Assay Kit with WST-8 and LDH Cytotoxicity Assay Kit were obtained from Beyotime Biotechnology, China. All the tested compounds were dissolved in DMSO as stock solution, and diluted to the expected experimental concentrations containing 1% (v/v) DMSO. In each experiment, vehicle control (1% (v/v) DMSO) was used as the reference group unless otherwise specified.

Rabbit anti-NLRP3 (15101), Rabbit anti-AIM2 (12948), Rabbit anti-ASC/TMS1 (13833), Rabbit anti-Cleaved Caspase-1 (4199), Rabbit anti-Caspase-1 (3866), Rabbit anti-Cleaved Caspase-7 (8438), Rabbit anti-Caspase-7 (9492), Rabbit anti-Cleaved Caspase-8 (98134), Rabbit anti-Cleaved Caspase-9 (20750), Rabbit anti-Phospho-RIP (65746), Rabbit anti-RIP (3493), Rabbit anti-Phospho-MLKL (91689), Rabbit anti-MLKL (14993), Rabbit anti-cGAS (15102), Rabbit anti-Phospho-STING (50907), Rabbit anti-STING (13647), Rabbit anti-Phospho-IRF3 (29047), Rabbit anti-IRF3 (11904), Rabbit anti-Phospho-TBK1/NAK (5483), Rabbit anti-TBK1/NAK (3504), Rabbit anti-Phospho-NF-κB P65 (3033), Rabbit anti-NF-κB P65 (8242), Rabbit anti-Phospho-IκBα (2859), Mouse anti-IκBα (4814), Rabbit anti-GPX4 (52455), Rabbit anti-β-actin (4970), Rabbit anti-Vinculin (13901), Goat Anti-Mouse IgG (H+L) (91196) and Goat Anti-Rabbit IgG (H+L)-HRP (7074) were purchased from Cell Signaling. Rabbit anti-Cleaved Caspase-3 (ab32042), Rabbit anti-Cleaved N-terminal GSDMD (ab215203), Rabbit anti-Cleaved N-terminal GSDME (ab222408), Alexa Fluor® 488 Rabbit anti-HMGB1 (ab195010), Alexa Fluor® 647 Rabbit anti-CRT (ab196159), Mouse anti-dsDNA (ab27156), Alexa Fluor® 488 Goat Anti-Rabbit IgG (H+L) (ab150077), Alexa Fluor® 647 Goat Anti-Mouse IgG (H+L) (ab150119) were purchased from Abcam.

2. General Instruments and methods

All reagents were obtained from commercial sources and used without additional purification. NMR spectra were recorded at 298K on Bruker AVANCE III 400 (400 MHz) with TMS as the reference. The data were processed by MestReNova and TopSpin 4.0.6 software. HR-ESI-MS spectra were measured on Bruker maXis 4G ESI-Q-TOF with Tunning mix as calibrating agent.

Data analysis was processed on Bruker Data Analysis 5.1 software. And the diffraction data were collected on a XtalAB Synergy R, DW system, HyPix diffractometer, with Cu Kα radiation (λ = 1.54184 Å). All of the structures were solved by direct methods and refined by full-matrix least squares against F^2 using the SHELXL programs. The benzene ring atoms and pyridine ring atoms were geometrically restrained to fit idealized six membered, respectively. DFIX, SIMU, DELU and ISOR restrains were used to obtain reasonable parameters due to the poor quality of crystal data. SQUEEZE and PLATON program were employed to remove the contribution of the electron density associated with these highly disordered solvent molecules and counter anions.

3. Synthetic route

3.1 Ligand L^{oz} (2-(pyridin-3-yl)oxazolo[4,5-f][1,10]phenanthroline)

1,10-phenanthroline-5,6-dione (210 mg, 1 mmol), 3-Pyridinecarboxaldehyde (107 mg, 1 mmol), ammonium acetate (92.5 mg, 1.2 mmol) were suspended in 30 mL of ethanol. The mixture was then heated at 85°C for 12 h under N_2 and then cooled to ambient temperature. The residue formed was dissolved in H_2O and extracted with CH_2Cl_2 (3 × 15 mL). The combined organic layers dried over Na_2SO_4, and concentrated in vacuo. The resulting solid was purified by flash chromatography on silica gel to give Ligand L^{oz}. (156 mg, 0.52 mmol, 52%). ^{1}H NMR (400 MHz, Methanol-d_4) δ 9.37 (s, 1H), 9.03 (m, 2H), 8.78 (d, J = 4.9 Hz, 1H), 8.74 (d, J = 8.1 Hz, 1H), 8.61 (d, J = 8.2 Hz, 1H), 7.79 (td, 2H), 7.69 (dd, J = 8.1, 4.9 Hz, 1H). ^{13}C NMR (101 MHz, Methanol-d_4) δ 151.23, 149.10, 148.80, 147.20, 134.69, 130.24, 128.55, 124.27, 123.61, 123.59, 48.24, 48.02, 47.81, 47.73, 47.60, 47.39, 47.17, 46.96. HR-ESI-MS m/z: $[M+H]^+$ Calcd. for $C_{18}H_{10}N_4O$, 299.0927, Found: 299.0967.

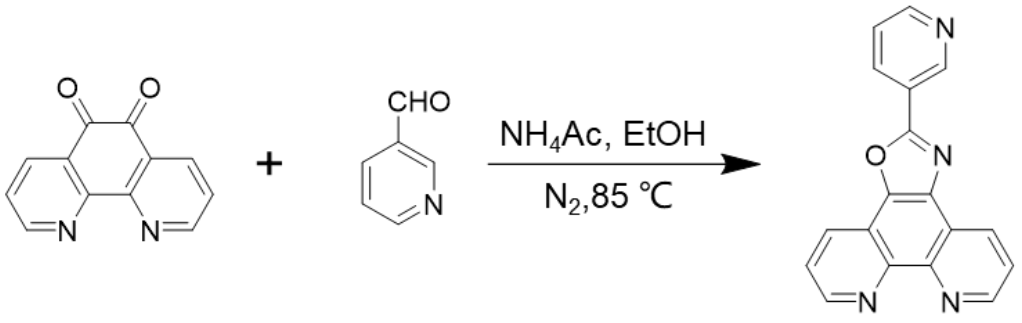

Figure S1. Synthetic route of L^{oz}.

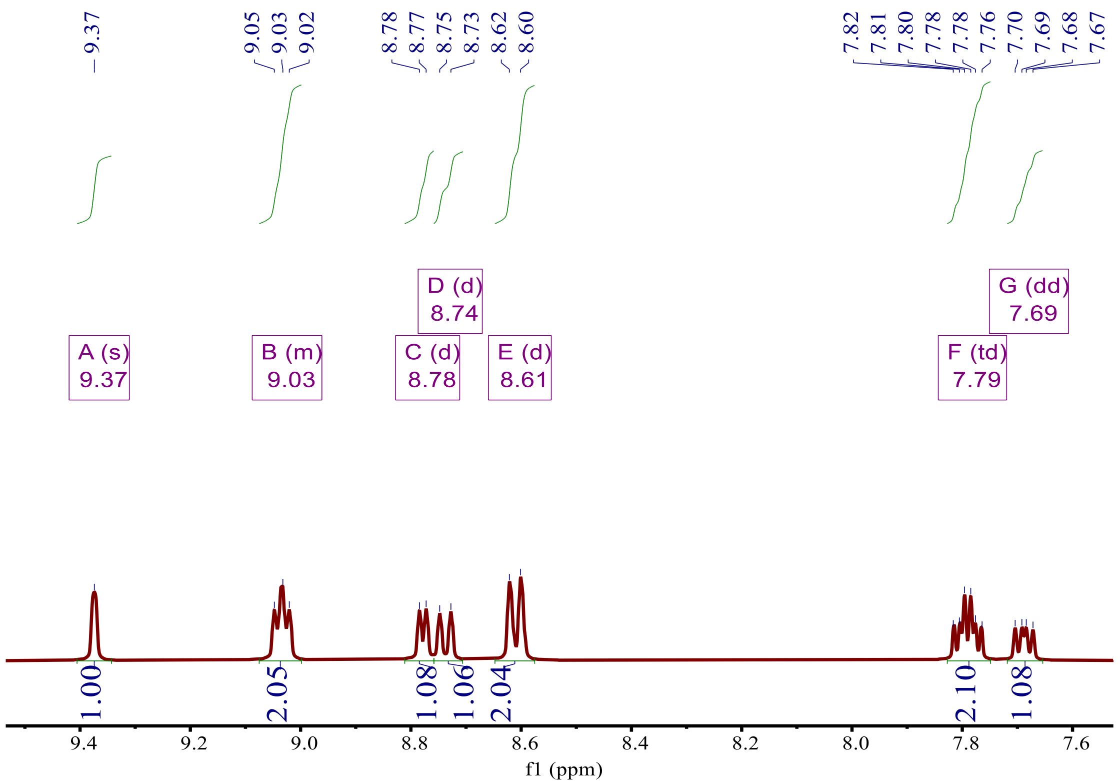

Figure S2. ^{1}H NMR spectrum of L^{oz} (400 MHz, methanol-d_4, 298 K).

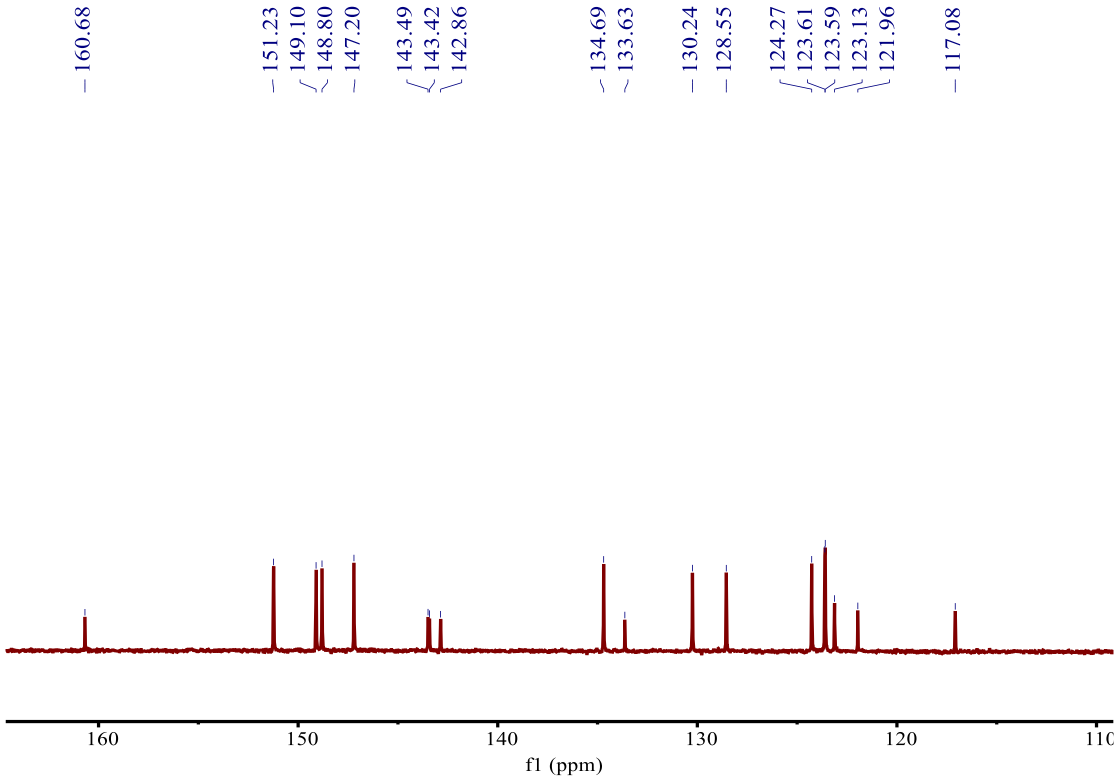

Figure S3. ^{13}C NMR spectrum of L^{oz} (101 MHz, methanol-d_4, 298 K).

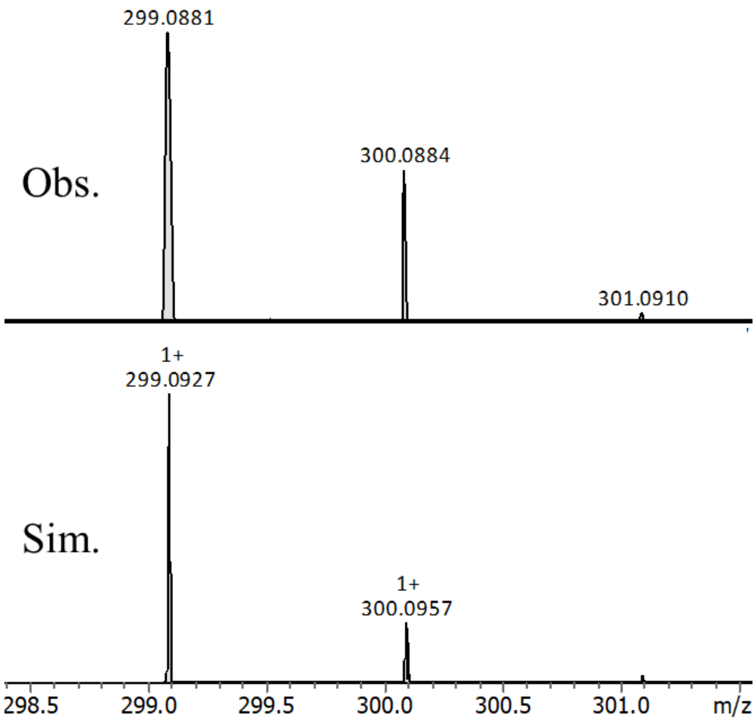

Figure S4. HR-ESI-TOF-MS spectra of L^{oz} in CH_3CN.

3.2 Metalloligand $RuL^{oz}_3(BF_4)_2$

A 50 mL microwave flask was charged with 30 mL ethylene glycol, $RuCl_3 \cdot 3H_2O$ (261 mg, 1 mmol) and L (895 mg, 3 mmol), the reaction mixture was heated under microwave at 190°C for 30 minutes. After cooling to room temperature, 30 mL water and 3 mL saturated $NaBF_4$ aqueous solution were added into the resulting orange red solution. The orange precipitate was filtered off and washed with water twice and dried in vacuo to yield $RuL^{oz}_3(BF_4)_2$ (250 mg, 0.2 mmol, 80%) as red powder. ^1H NMR (400 MHz, $DMSO_{-d6}$) δ 9.61 (s, 1H), 9.14 (d, *J* = 8.3 Hz, 2H), 8.92 (d, *J* = 4.9 Hz, 1H), 8.77 (d, *J* = 8.2 Hz, 1H), 8.24 (s, 2H), 7.93 (m, 2H), 7.79 (dd, *J* = 8.1, 4.9 Hz, 1H). ^{13}C NMR (101 MHz, CD_3CN) δ 163.68, 152.87, 152.76, 148.48, 147.04, 146.68, 144.55, 136.30, 134.80, 131.47, 129.77, 126.62, 125.02, 124.32, 122.80, 120.16, 117.35. HR-ESI-MS m/z: $[M-2BF_4]^{2+}$ Calcd. for $C_{54}H_{30}N_{12}O_3Ru$, 498.0805, Found: 498.0801.

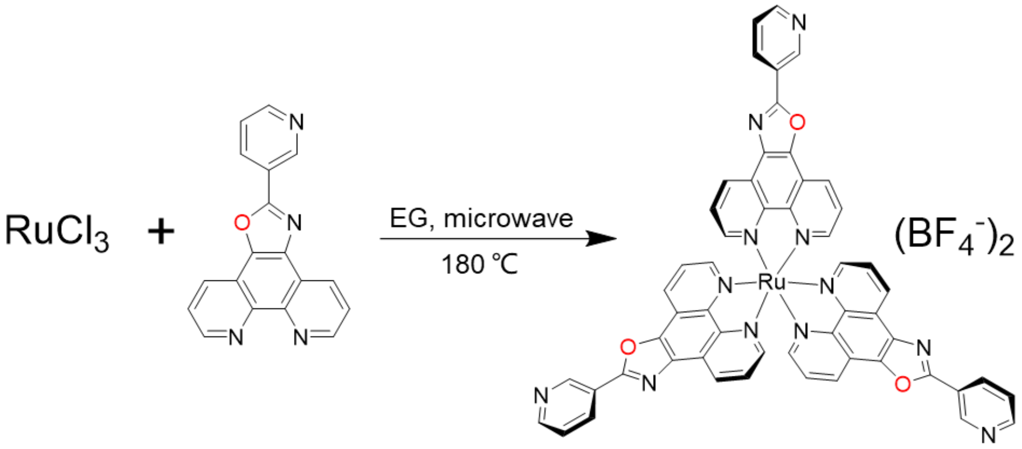

Figure S5. Synthetic route of $RuL^{oz}_3(BF_4)_2$.

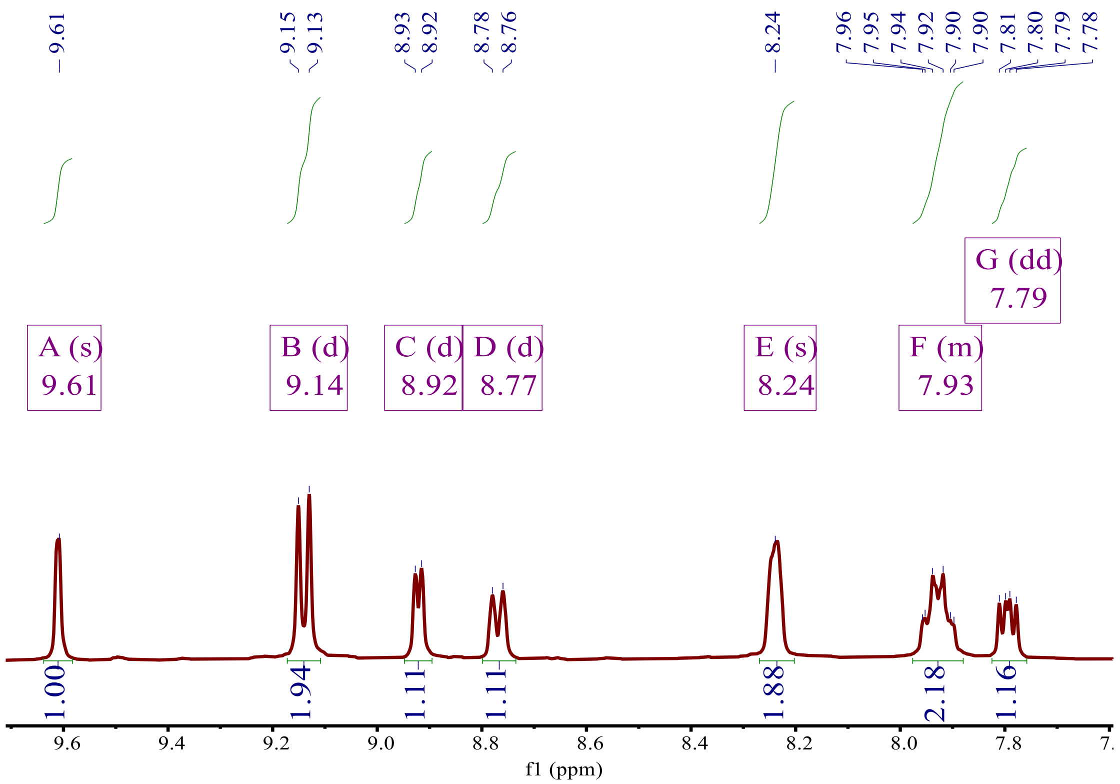

Figure S6. ^{1}H NMR spectrum of $RuL^{oz}_3(BF_4)_2$ (400 MHz, DMSO-d_6, 298 K).

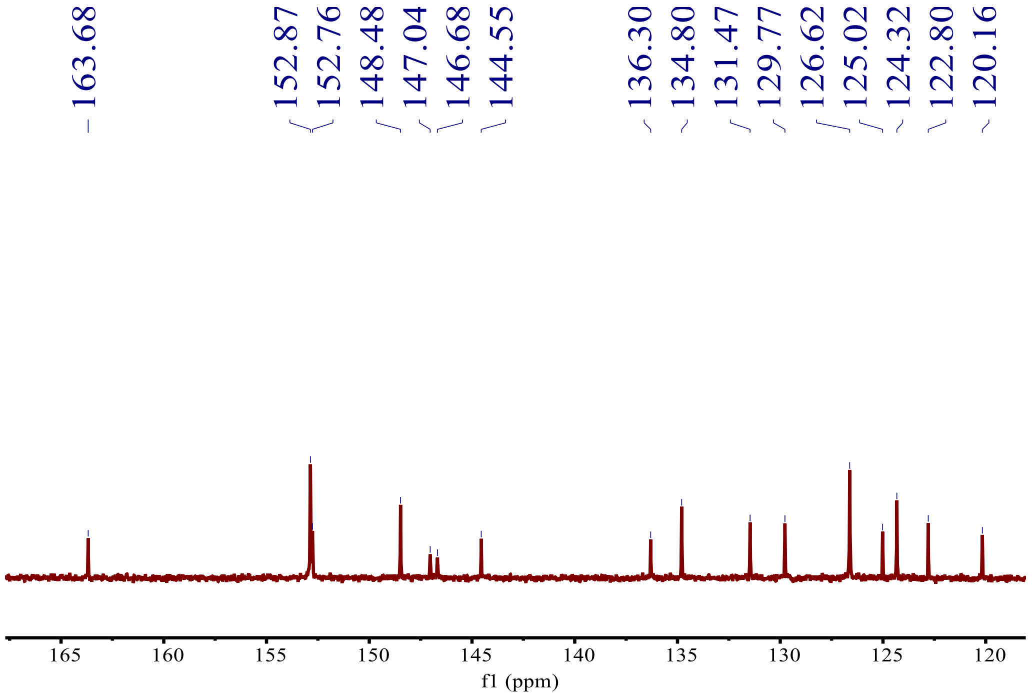

Figure S7. ^{13}C NMR spectrum of $RuL^{oz}_3(BF_4)_2$ (101 MHz, DMSO-d_6, 298 K).

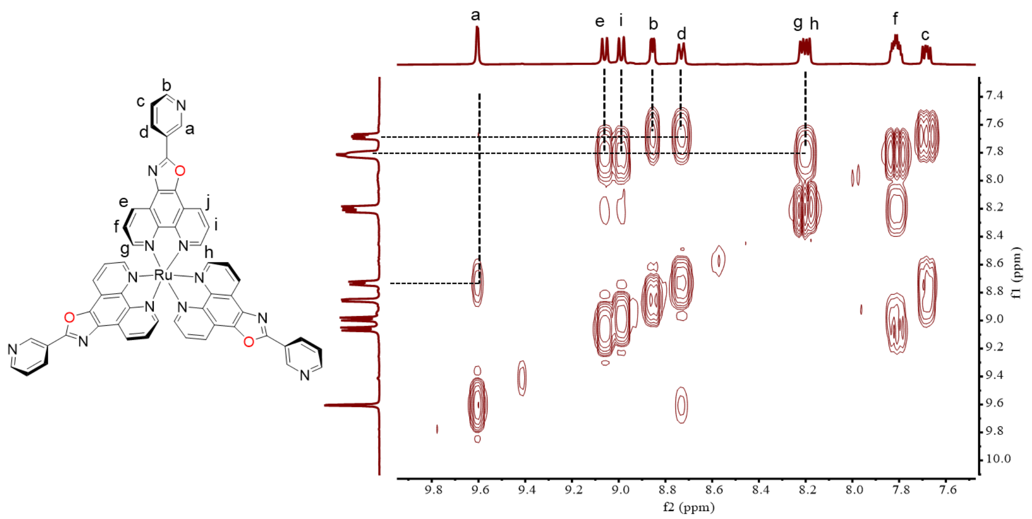

Figure S8. ^{1}H-^{1}H COSY NMR spectrum of $RuL^{oz}{}_3(BF_4)_2$ (400 MHz, CD_3CN, 298 K).

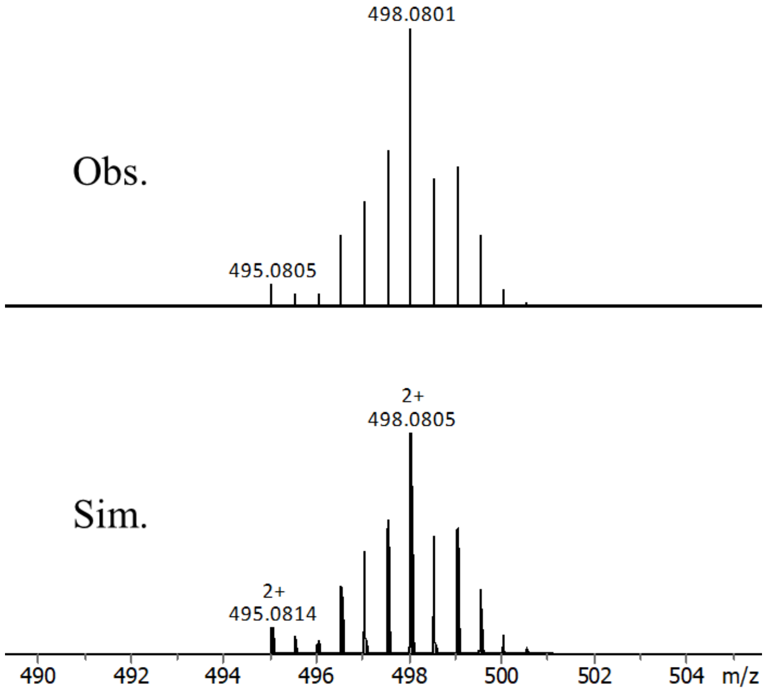

Figure S9. HR-ESI-TOF-MS Spectra of $RuL^{oz}{}_3(BF_4)_2$ in CH_3CN.

3.3 Self-assembly and characterization of MOCs

$Pd(BF_4)_2(CH_3CN)_4$ (106.62 mg, 240 μmol,) was dissolved into 5 mL CH_3CN, and then added to a solution of $RuL^{oz}{}_3(BF_4)_2$ (374.27 mg, 320 μmol, 1 eq.) in CH_3CN (15 mL). The mixture was heated and stirred at 80 °C for 1 h. After the reaction has completed, the mixture was cooled and filtered to remove insoluble impurities. The filtrate was poured into 1000 mL ether under stirring to provide large quantity of precipitates. The precipitates were filtered, washed with ether to give Ru_8Pd_6 as a red solid. Single crystals of the structures suitable for X-ray diffraction analysis were obtained by the diffusion of CH_3OH into 400 μL MOC-88 CH_3CN solution.

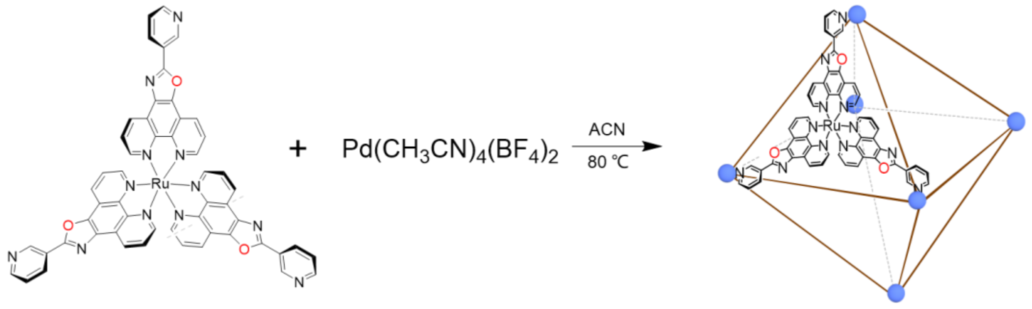

Figure S10. Synthetic route of MOC-88.

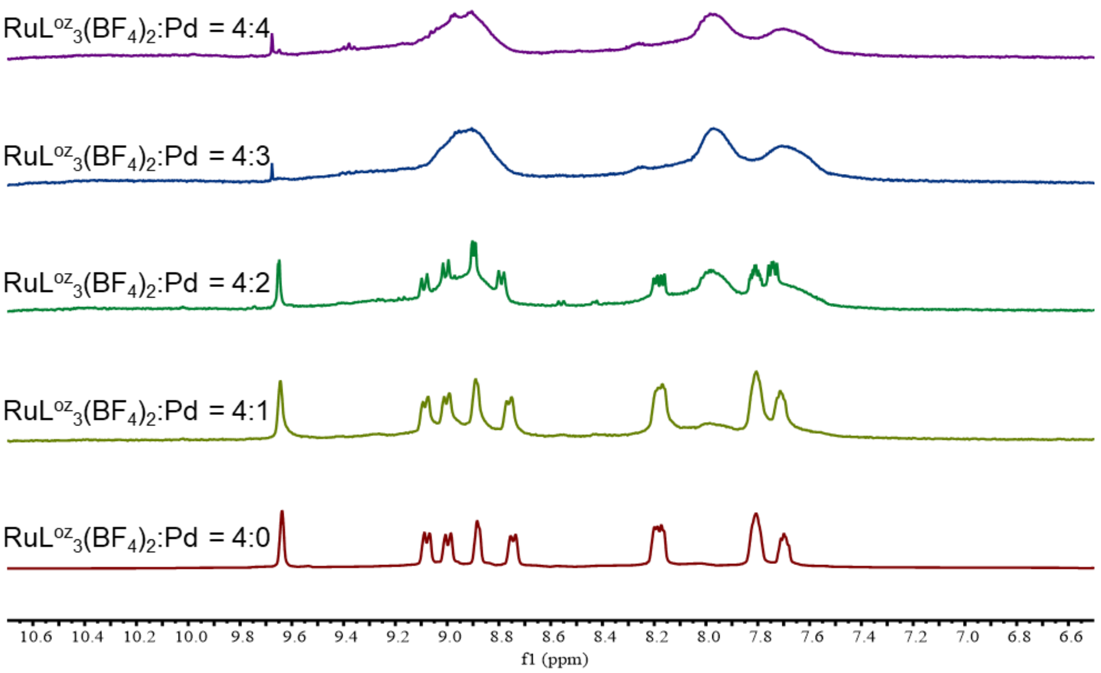

Figure S11. ^{1}H NMR spectrum of MOC-88 (400 MHz, DMSO-d_6, 298 K).

4. X-ray Single Crystal Structural Analysis

Single-crystal X-ray diffraction data for all samples were collected on a Rigaku Oxford SuperNova X-RAY diffractometer system. The structure was solved by direct methods, and refined by full-matrix least-square methods with the SHELXL-2014 program package and OLEX2 program. All hydrogen atoms were located in calculated positions and refined anisotropically. The crystallographic data for all compounds were listed in Table S1. The single crystal data have been deposited in the Cambridge Crystallographic Data Center (CCDC code: 2280671).

Table S1. Crystal data and structure refinement for **MOC-88**.

Identification Code	**MOC-88**
Empirical formula	$C_{432}H_{240}N_{96}O_{24}Pd_6Ru_8$
Formula weight	8606.15
Temperature/K	200.00(10)
Crystal system	tetragonal
Space group	*P*4/n
a/Å	29.8804(2)
b/Å	29.8804(2)
c/Å	42.1847(6)
α/°	90
β/°	90
γ/°	90
Volume/Å^3	37664.1(7)
Z	2
ρ_{calc}g/cm^3	0.759
μ/mm^{-1}	2.709
F(000)	8648.0
Crystal size/mm^3	0.1 × 0.1 × 0.1
Radiation	Cu*K*α(λ = 1.54184)
2Θ range for data collection/°	8.37 to 103.584
Index ranges	$-30 \le h \le 27$, $-29 \le k \le 29$, $-39 \le l \le 42$
Reflections collected	86789
Independent reflections	20403 [*R*int = 0.0393, *R*sigma = 0.0326]
Data/restraints/parameters	20403/6/1258
Goodness-of-fit on F^2	1.086
Final *R* indexes [$I >= 2\sigma (I)$]	*R*1 = 0.0574, w*R*2 = 0.1880
Final *R* indexes [all data]	R1 = 0.0791, wR2 = 0.2037
Largest diff. peak/hole / e Å^{-3}	0.58/-0.31

5.UV-Vis and Fluorescence Spectra of $RuL^{oz}{}_3(BF_4)_2$ and MOC-88

The UV-Vis absorption spectra of $RuL^{oz}{}_3(BF_4)_2$ (8 μM) and MOC-88 (1 μM) were measured using Shimadzu UV-3600 spectrophotometer. The florescence emissions spectra of $RuL^{oz}{}_3(BF_4)_2$ (32 μM) and MOC-88 (4 μM) were obtained using FLS 980 combined fluorescence steady state (λ_{ex} = 458 nm) and lifetime with a laser diode (λ_{ex} = 472 nm). Frequency domain data were analyzed with a sum of two discrete exponential lifetimes (τ_1 and τ_2) that were intensity-weighted (f_1 and f_2), and the results were evaluated using the χ^2 value (optimal χ^2 was about 1). The average lifetime τave was calculated using the equation: $\tau ave= \tau_1 \times f_1 + \tau_2 \times f_2$. The absolute fluorescence quantum yields of $RuL^{oz}{}_3(BF_4)_2$ and MOC-88 were measured on a Hamamatsu Absolute Quantum Yield Spectrometer C9920.

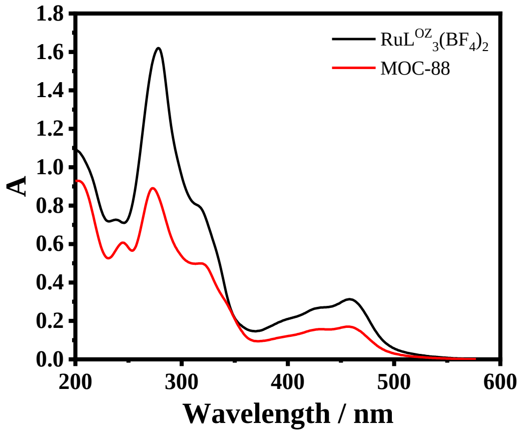

Figure S12. UV-vis absorption spectra of RuL$^{oz}_3(BF_4)_2$ (8 μM) and MOC-88 (1 μM) in PBS solution.

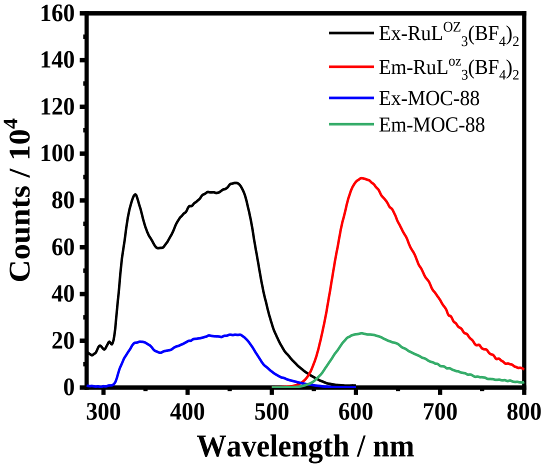

Figure S13. Photoluminescent excitation and emission spectra of RuL$^{oz}_3(BF_4)_2$ (32 μM) and MOC-88 (4 μM) in PBS solution.

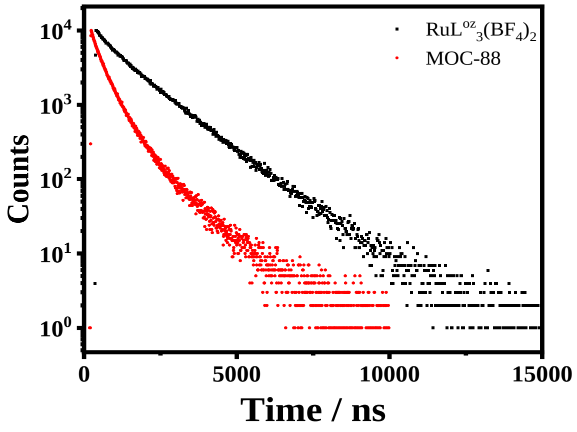

Figure S14. Decay lifetimes spectra of RuL$^{oz}_3(BF_4)_2$ (32 μM) and MOC-88 (4 μM) in PBS solution (λex = 472 nm).

Table S2. Absolute emission quantum yield (QY/%) with different excitation for $RuL^{oz}{}_3(BF_4)_2$ and MOC-88 in DMSO solution.

Excitation	$RuL^{oz}{}_3(BF_4)_2$	MOC-88
400 nm	0.090	0.076
410 nm	0.088	0.083
420 nm	0.088	0.08
430 nm	0.086	0.086
440 nm	0.086	0.087
450 nm	0.087	0.088
460 nm	0.087	0.088
470 nm	0.085	0.087
480 nm	0.085	0.088
490 nm	0.081	0.08
500 nm	0.075	0.074
510 nm	0.072	0.082
520 nm	0.059	0.077
530 nm	0.071	0.054
540 nm	0.001	0.047
550 nm	0.001	0.019

6. Measurement of two-photon absorption (TPA) cross-section

The TPA cross-section of $RuL^{oz}{}_3(BF_4)_2$ and MOC-88 were determined as previously reported. The two-photon excited luminescence spectra were obtained with Astrella/OperA Solo femtosecond-laser with a broad region from 650 nm to 1200 nm by using Rhodamine B in methanol as the reference. Fluorometric quartz cuvettes were used to measure the two-photon luminescence. The luminescence intensity of samples and Rhodamine B were measured under the same excitation wavelength and detection condition. The TPA cross sections were obtained based on the following equation.

$$\delta_s = \delta_r \cdot \frac{\emptyset_r \cdot c_r \cdot I_s \cdot n_s}{\emptyset_s \cdot c_s \cdot I_r \cdot n_r}$$

I is the integrated fluorescence intensity, *c* is the concentration, *n* is the refractive index, *Φ* is the quantum yield, subscript 'r' stands for reference samples, and 's' stands for the samples.

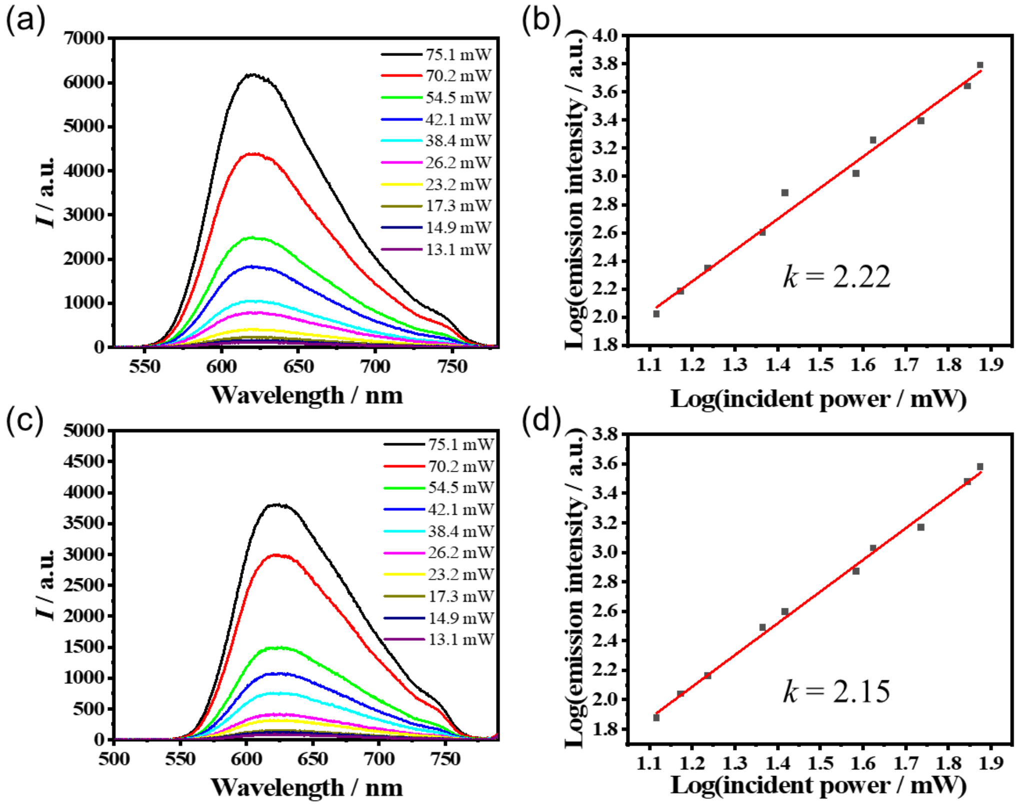

Figure S15. (a) The power-dependent emission spectrum of $RuL^{oz}_3(BF_4)_2$ (3.2 mM) in DMSO solution (λex = 810 nm). (b) the corresponding Log (PL Intensity)-Log (Power) relationship of $RuL^{oz}_3(BF_4)_2$. (c) The power-dependent emission spectrum of MOC-88 (0.4 mM) in DMSO solution (λex = 810 nm). (d) the corresponding Log (PL Intensity)-Log (Power) relationship of MOC-88.

7. pH Stability

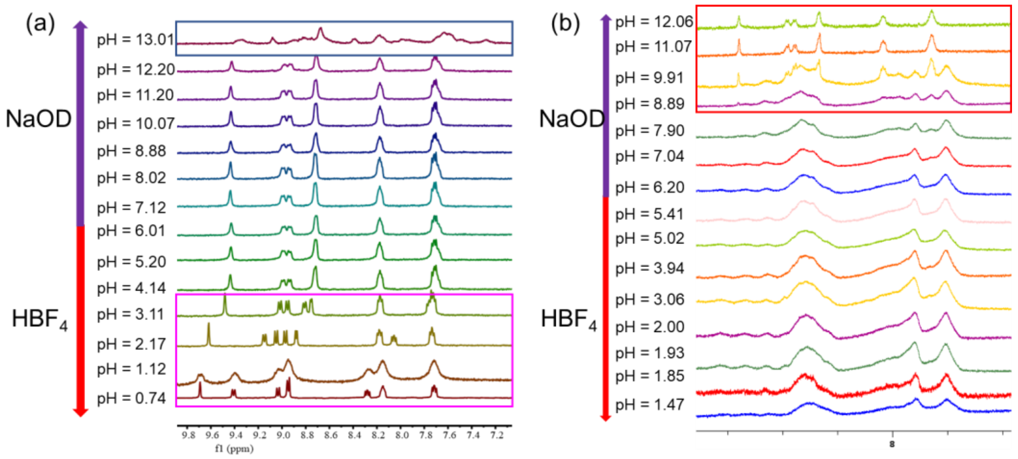

Figure S16. ^1H NMR spectrum of (a) 1.6 mM $RuL^{oz}_3(BF_4)_2$ and (b) 0.2 mM MOC-88 in DMSO-d_6: D_2O = 1: 4 (v/v) media with different pH values.

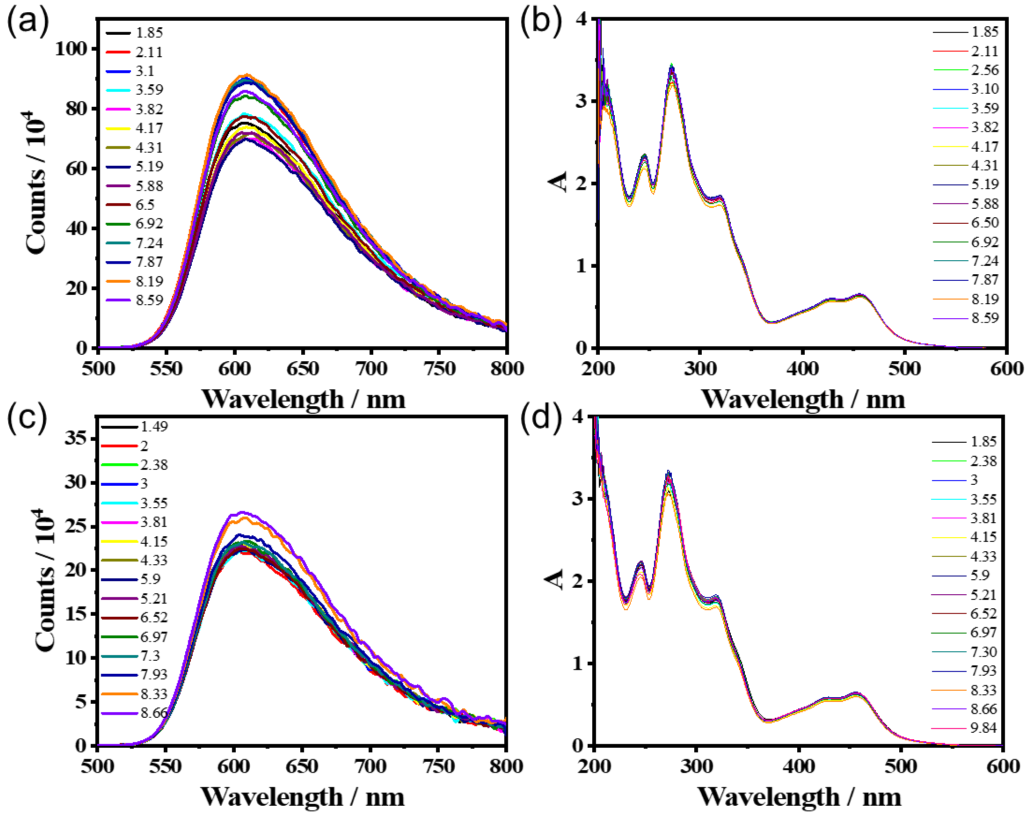

Figure S17. Fluorescence spectrum of (a) $RuL^{oz}_3(BF_4)_2$ (32 μM) and (c) MOC-88 (4 μM). UV-Vis spectrum of (b) $RuL^{oz}_3(BF_4)_2$ (32 μM) and (d) MOC-88 (4 μM) in PBS solutions at different pH values.

8. MOC-88 stability in *vitro*

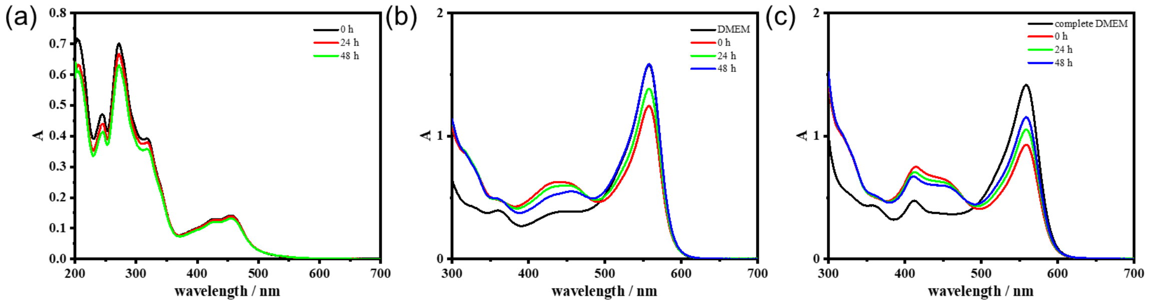

Figure S18. UV-vis absorption spectra of the stability of MOC-88 (1 μM) in (a) PBS, (b) DMEM or (c) complete DMEM solution.

9. ROS detection

9.1 Singlet oxygen ($^1O_2$) detection

9.1.1 ESR Experiment

Sample solutions (10 μL) were put in glass capillary tubes with internal diameters of 0.5 mm and sealed. The tubes were inserted into the ESR cavity and measured at selected times. ESR parameter settings were as follows: modulation amplitude 1 G, scan range 150 G, microwave power 0.08 mW and time constant 30 ms for detection of spin adducts using spin traps TEMP. Tubes containing 100 mM TEMP and 8 mM $RuL^{oz}_3(BF_4)_2$ (or 1 mM MOC-88) in DMF were exposed to 450-460 nm LED (20 mW/cm^2) for 2 min and immediately characterized.

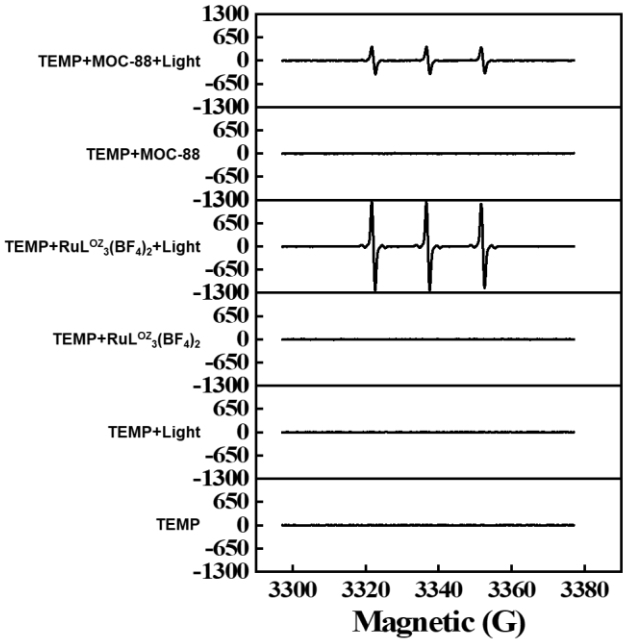

Figure S19. ESR signals obtained upon irradiation (450-460 nm, 20 mW/cm^2) for 2 min including 100 mM TEMP and 8 mM $RuL^{oz}_3(BF_4)_2$ (or 1 mM MOC-88) in DMF.

9.1.2 $^1O_2$ Quantum Yield

The singlet oxygen capture agent (ABDA) was used to measure the singlet oxygen generated by $RuL^{oz}_3(BF_4)_2$, MOC-88. Firstly, the absorbance of $RuL^{oz}_3(BF_4)_2$ and MOC-88 at 460 nm was adjusted to about 0.15 in PBS. Secondly, 100 μM ABDA (the final concentration) was added in the solution to one quartz cuvette. Thirdly, the cuvette was irradiated with 450-460 nm laser (4 mW/cm^2) for different time. And the UV-Vis absorption spectra were recorded immediately. The quantum yields for $^1O_2$ production (Φ_Δ) of $RuL^{oz}_3(BF_4)_2$, MOC-88 under irradiation in aerated solution were calculated using $Ru(bpy)_3Cl_2$ as the standard (Φ_Δ = 0.18 in PBS)

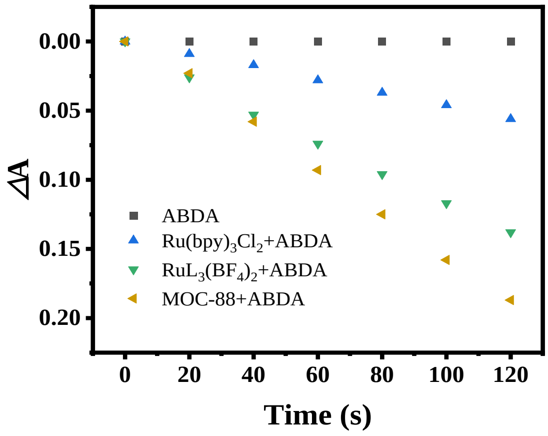

Figure S20. Rate of decay of ABDA sensitized by $RuL^{oz}{}_3(BF_4)_2$, MOC-88, and $Ru(bpy)_3Cl_2$ in pH 7.4 PBS as shown by the decrease in the absorption maxima of ABDA.

9.2 Hydroxyl radical (·OH) detection

9.2.1 ESR Experiment

Sample solutions (10 µL) were put in glass capillary tubes with internal diameters of 0.5 mm and sealed. The tubes were inserted into the ESR cavity and measured at selected times. ESR parameter settings were as follows: modulation amplitude 1 G, scan range 150 G, microwave power 0.08 mW and time constant 30 ms for detection of spin adducts using spin traps BMPO. Tubes containing 250 mM BMPO and 8 mM $RuL^{oz}{}_3(BF_4)_2$ (or 1 mM MOC-88) in DMF were exposed to 450-460 nm LED (20 mW/cm^2) for 2 min and immediately characterized.

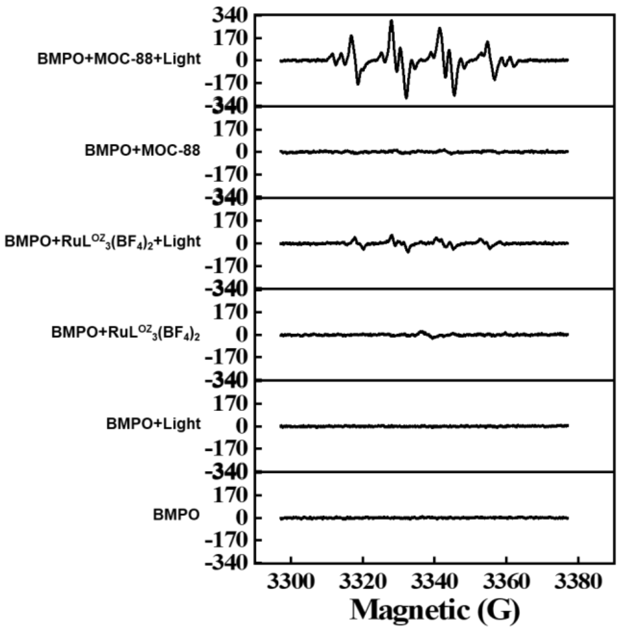

Figure S21. ESR signals obtained upon irradiation (450-460 nm, 20 mW/cm^2) for 2 min including 250 mM BMPO and 8 mM $RuL^{oz}{}_3(BF_4)_2$ (or 1 mM MOC-88) in DMF.

9.2.2 Hydroxyphenyl fluorescein Experiment

Hydroxyphenyl fluorescein (HPF) is used as the hydroxyl radical (·OH) indicator, which can be changed from colorless to green fluorescence in the presence of ·OH. $RuL^{oz}_3(BF_4)_2$ (8 μM), MOC-88 (1 μM) and HPF (10 μM) were prepared in pH 7.4 or 6.0 PBS solution. Then the cuvette was exposed to 450-460 nm laser (4 mW/cm^2) for different time, and the fluorescence spectra were recorded immediately.

Measurement in a nitrogen environment: After mixing all solutions in a dark environment, add nitrogen to the mixed solution for at least 30 minutes, and then immediately seal the colorimetric dish before conducting experimental testing.

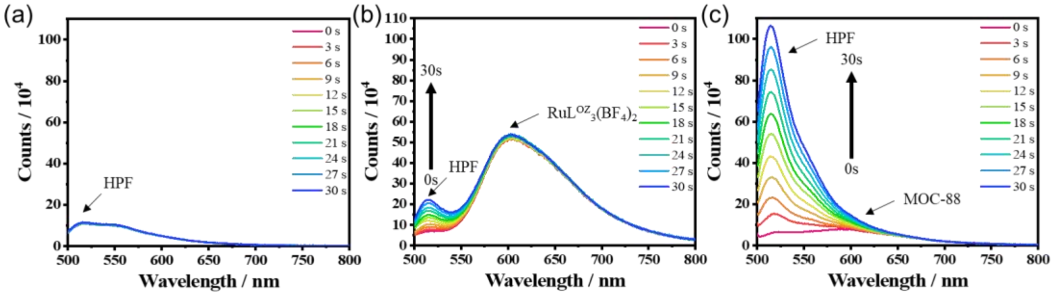

Figure S22. Under normal oxygen, fluorescence spectra of (a) HPF (10 μM), (b) $RuL^{oz}_3(BF_4)_2$ (8 μM) + HPF (10 μM) and (c) MOC-88 (1 μM) + HPF (10 μM) in PBS for ·OH using HPF as fluorescence probe upon irradiation (450-460 nm, 4 mW/cm^2).

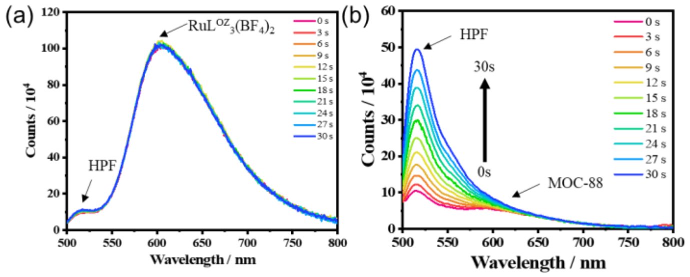

Figure S23. Under nitrogen environment, fluorescence spectra of (a) $RuL^{oz}_3(BF_4)_2$ (8 μM) + HPF (10 μM) and (b) MOC-88 (1 μM) + HPF (10 μM) in PBS for ·OH using HPF as fluorescence probe upon irradiation (450-460 nm, 4 mW/cm^2).

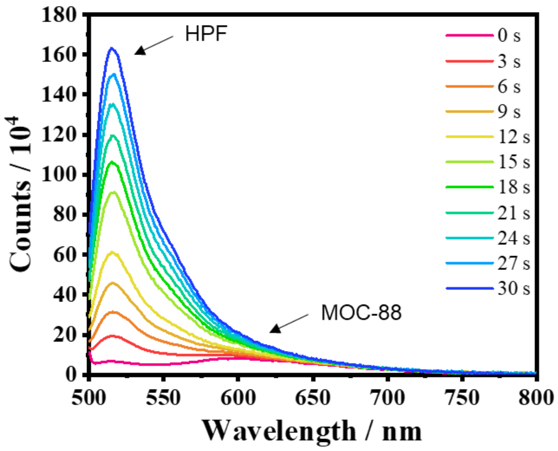

Figure S24. Phosphorescence spectra of MOC-88 (1 μM) generates •OH radical in pH 6.0 PBS under aerobic environments. Using HPF (10 μM) as fluorescence probe and 450-460 nm laser (4 mW/cm^2) for irradiation.

10. Cyclic Voltammetry

The cyclic voltammetry curve was obtained by CHI 760 electrochemical Workstation of Chenhua, China. The test system used a glass carbon electrode as the working electrode, a platinum plate electrode as the counter electrode, a non aqueous Ag/AgCl as the reference electrode. The supporting electrolyte was 0.1 M tetrabutylammonium hexafluorophosphate ($TBAPF_6$). The scanning speed was 100 mV/s.

11.Determination of the lipophilicity

The lipophilicity of MOC-88 was determined by a shake-flask method as previously reported. The lipophilicity was presented as octanol/water partition coefficients Log $P_{o/w}$ valves, which was defined as the logarithmic ratio of the MOC-88 concentration in octanol phase to that in the aqueous phase. Step 1: 50 mL of n-octanol and 50 mL of water was mixed, and the mixture solution was shaken at room temperature for two days to obtain fully saturated octanol phase and water phase. Step 2: 5 mg of MOC-88 was dispersed in 6 mL saturated octanol phase and shaken at 150 rpm for 24 h, then the octanol solution containing MOC-88 was centrifuged to remove the insoluble samples. 5 mL of supernatant in octanol layer was gently taken out and mixed with 5 mL of saturated water phase, followed by another 24 h shaking at room temperature with 150 rpm of speed. Step 3: the water layer and octanol layer were carefully separated for the next analysis. The concentration of samples in the octanol phase and water phase were measured by using UV-Vis spectrophotometer.

12. In *vitro* experiment

12.1 Cell Culture

The cells (Hela, HLF-a, 3T3-L1 and U14) were obtained from Experimental Animal Center of Sun Yat-Sen University, China. Cells were cultured in DMEM medium with 10% FBS, 100 μg/mL streptomycin and 100 U/mL penicillin as supplement. Cells were cultured at 37 °C in a humidified incubator with an atmosphere of 5% CO_2 and 95% air (normoxia) or 1% oxygen, 5% carbon dioxide and 94% nitrogen (hypoxia).

12.2 Cell viability assay and In *vitro* cytotoxicity

The cytotoxicity of the tested compounds against Hela and U14 was measured by the MTT assay as previously described. Cells were seeded in 96-well plates, and incubated for 24 h at normoxia or hypoxia before treated with the tested compounds. The cells were incubated with tested compounds at the indicated concentrations for 24 h in dark, and for the light cytotoxicity they were irradiated with a 450-460 nm (20 mW/cm^2, 10 min, 12 J/cm^2) after 3 hours' incubation. Then, 20 μL MTT (5 mg/mL) was added to each well, and incubated for another 4 h. The medium was removed and DMSO was added (150 μL per well). The absorbance at 570 nm was determined using a microplate reader. The percentage of cell viability was calculated using the equation: (mean OD of treated cells/mean OD of control cells) × 100%.

Table S3. Cytotoxicity (IC_{50}, μM) of tested MOC-88 against normal cell lines for 24 h in the absence and presence of 450-460 nm irradiation (20 mW/cm^2, 10 min) upon treatment with MOC-88 for 3 h in normoxia (mean ± SD, *n* = 3).

Compound	Dark (μM)	Light (μM)	PI[a]
	**HLF-a (Normoxia)**		
$RuL^{oz}{}_3(BF_4)_2$	67.16 ± 1.02	13.10 ± 0.30	5.13
MOC-88[b]	84.51 ± 2.31	9.30 ± 0.26	9.09
	**3T3-L1 (Normoxia)**		
$RuL^{oz}{}_3(BF_4)_2$	120.72 ± 3.05	41.83 ± 5.13	2.89
MOC-88[b]	138.19 ± 3.32	31.18 ± 1.44	4.43

[a]Phototoxicity index (PI) is defined as the ratio of ${}^{dark}IC_{50}/{}^{light}IC_{50}$.
[b]Equivalent concentration of Ru-photocenter.

12.3Intracellular uptake by ICP-MS assay

Hela cells were seeded onto a 12 well plate and were grown in complete DMEM media in the cell incubator with 5% CO_2 atmosphere at 37 °C overnight. After the cells were treated with $RuL^{oz}{}_3(BF_4)_2$ (16 μM) or MOC-88 (2 μM) for different times, cells were washed for three times with sterile PBS. Subsequently, the cells were counted and digested in 400 μL of 60 % HNO_3 at 100 °C for 1 h. The solution was then diluted to a final volume of 10 mL with Milli-Q water. The concentration of Ru was measured using the XSERIES 2 ICP-MS. The standards for Ru calibration were freshly prepared by diluting a ruthenium standard solution with 2 % HNO_3 in Milli-Q water.

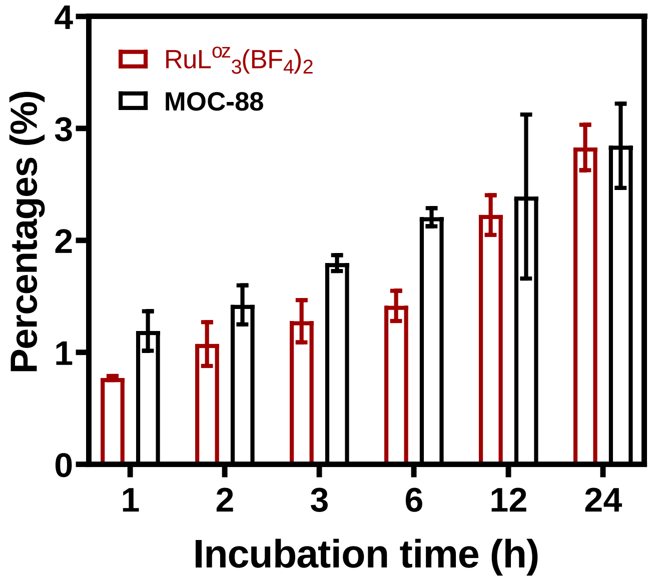

Figure S25. Cellular uptake of $RuL^{oz}_3(BF_4)_2$ (16 μM) or MOC-88 (2 μM) under different times measured by ICP-MS (mean ± SD, n = 3).

12.4 Intracellular uptake and localization measured by Confocal microscopy

Hela cells were seeded into 35 mm confocal dishes at a density of about 2 × 10^5 cells per well and incubated overnight at 37 °C in a 5% CO_2 incubator. For time-dependent uptake, concentration-dependent uptake and energy-dependent uptake, cells were incubated with MOC-88 (2 μM) for different time, with 3 h for different concentration of MOC-88 or with MOC-88 (4 μM, 3h) for different temperature. For colocalization studies, cells were incubated with MOC-88 (2 μM) for 3 h, and then stained with MTG (150 nM) or LTDR (150 nM) or BODIPY 493/503 (5 μM) or ER-Tracker Blue-White DPX (1 μM) or MDC (150 nM) for another 15 min. Cells were then washed for three time with PBS, and fresh medium was replenished before visualized by confocal microscopy. MOC-88: λex = 488 nm, λem = 625 ± 25 nm; LTDR: λex = 633 nm, λem = 678 ± 20 nm; ER-Tracker Blue-White DPX: λex=405 nm, λem =450±20 nm; MTG: λex=405 nm, λem =523±20 nm; BODIPY 493/503: λex=488 nm, λem =513±20 nm; MDC: λex=405 nm, λem =523±20 nm.

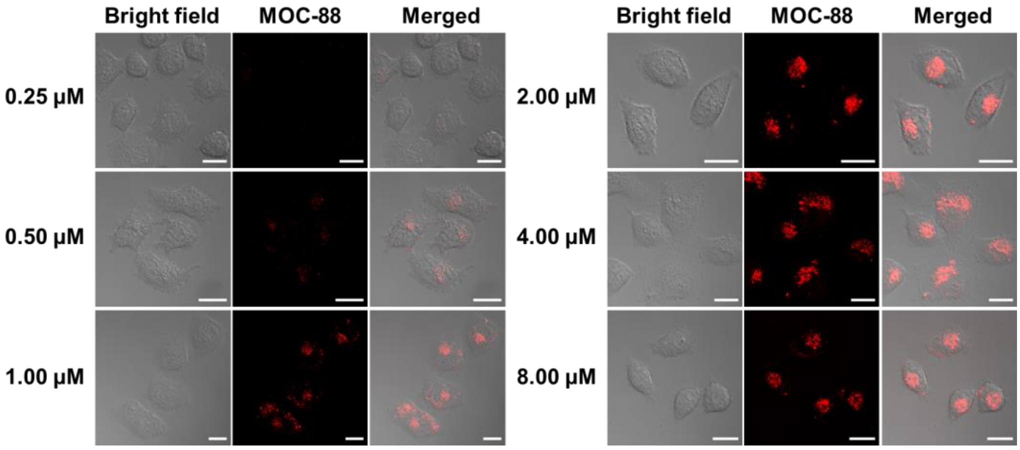

Figure S26. CLSM images of Hela cells collected at different concentration after MOC-88 (3 h) treatment. Scale bar: 20 μm.

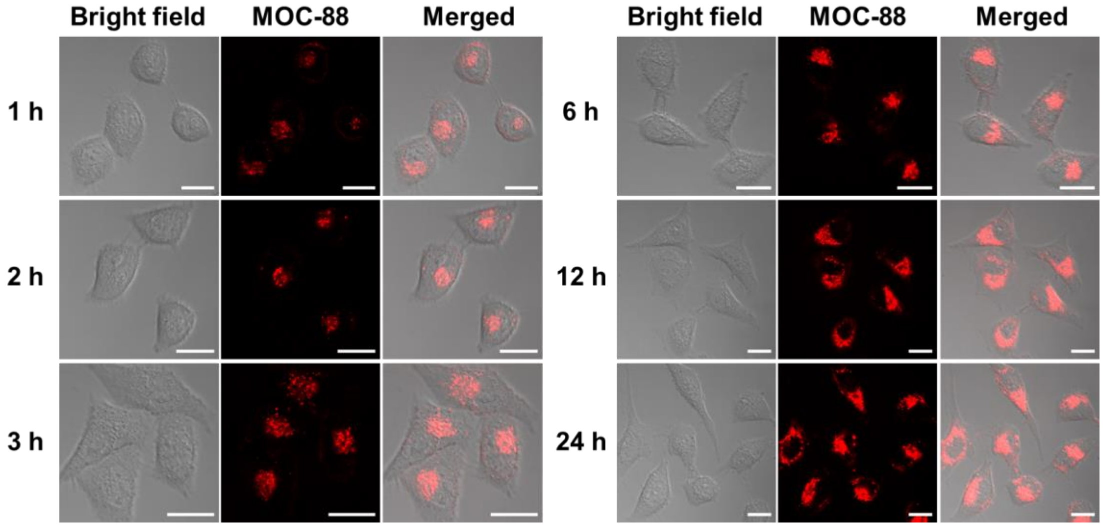

Figure S27. CLSM images of Hela cells collected at different time after MOC-88 (2 μM) treatment. Scale bar: 20 μm.

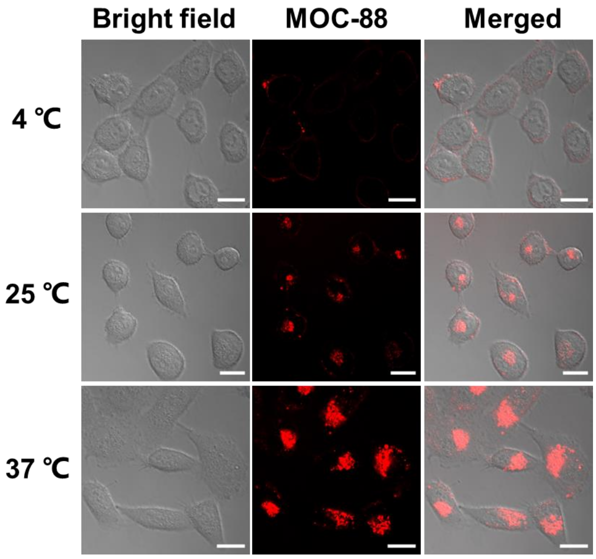

Figure S28. CLSM images of Hela cells collected at different temperature after MOC-88 (4 μM, 3 h) treatment. Scale bar: 20 μm.

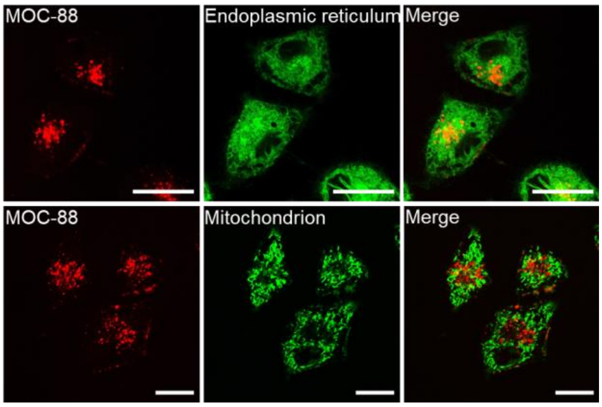

Figure S29. Co-localization of MOC-88 (2 μM, 3 h) and ER-Tracker Blue-White DPX (1 μM, 15 min) or MTG (150 nM, 15 min) in Hela cells. Scale bar: 20 μm.

12.5 Determination of Intracellular ROS levels in Hela cells

The intracellular ROS was determined by the DCFH-DA assay. Briefly, Hela cells were seeded into 35 mm confocal dishes at a density of about 2 × 10^5 cells per well and incubated overnight at 37 °C in a 5% CO_2 incubator. Upon treatment with different conentrations of MOC-88 for 3 h, these cells were irradiated with/without a 450-460 nm laser (20 mW/cm^2, 10 min). Then the cells were carefully rinsed twice with PBS and incubated with 10 μM DCFH-DA in FBS-free DMEM for 15 min in the dark. The cells were further rinsed twice with PBS and determined with confocal microscopy. DCF: λex = 488 nm, λem = 520 ± 20 nm.

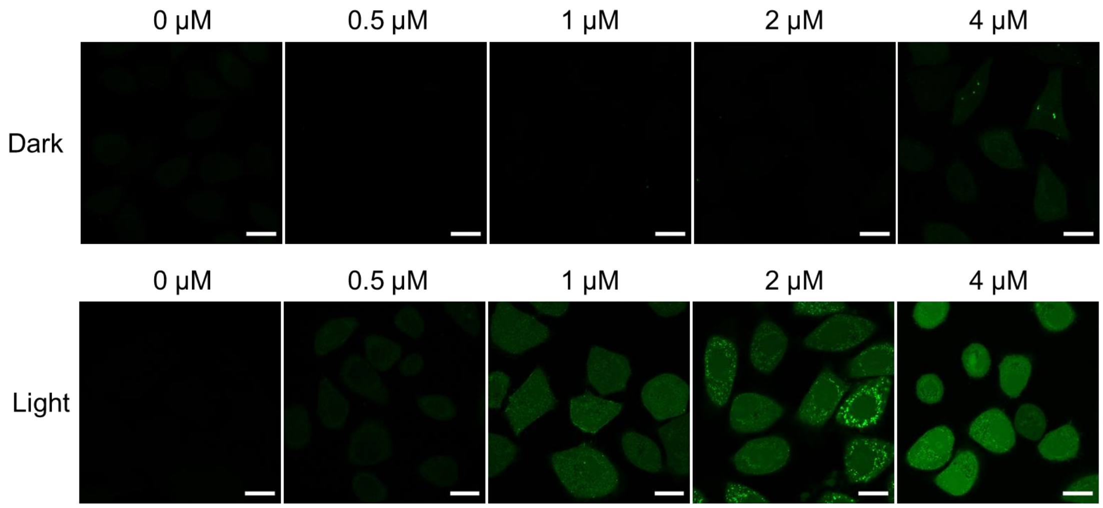

Figure S30. Intracellular ROS production of Hela cells after MOC-88 (3 h) with or without light (450-460 nm, 20 mW/cm^2) treatment by CLSM. Scale bar: 20 μm.

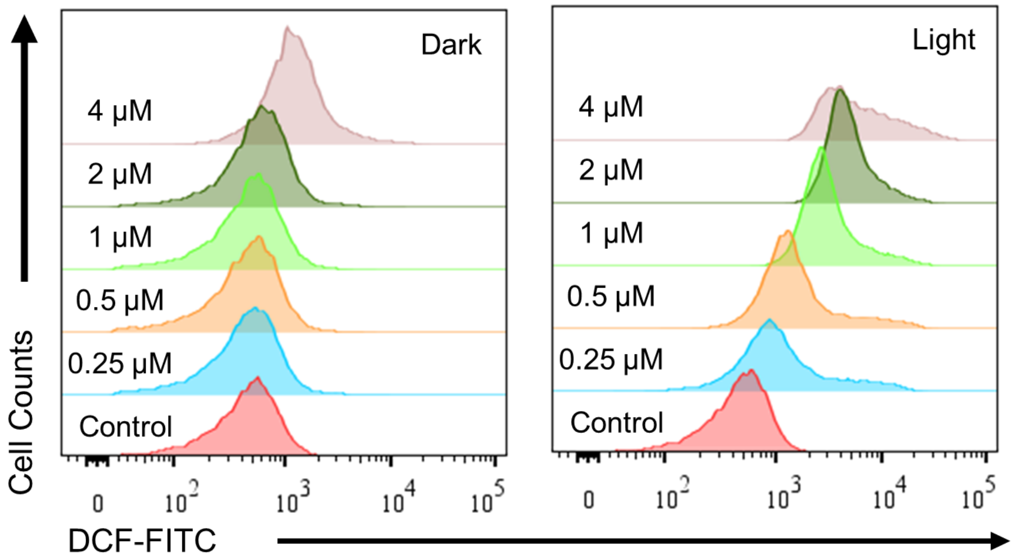

Figure S31. Intracellular ROS production of Hela cells after MOC-88 (3 h) with or without light (450-460 nm, 20 mW/cm^2) treatment by flow cytometry.

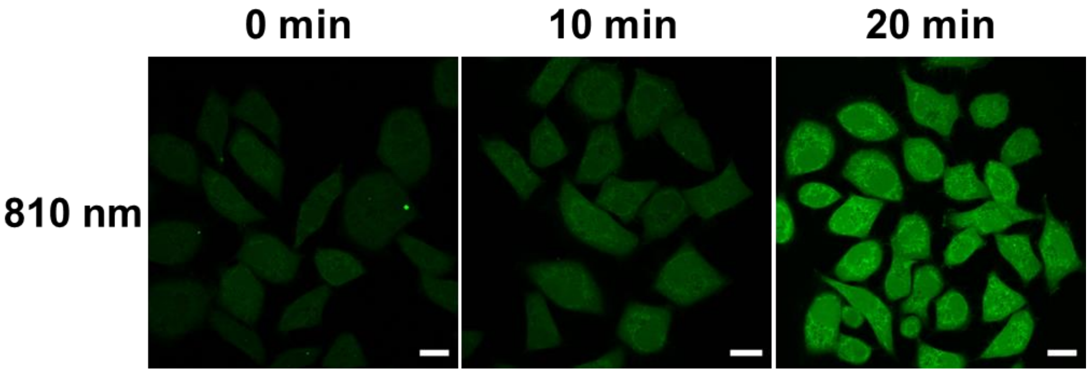

Figure S32. Intracellular ROS production of Hela cells after MOC-88 (4.0 μM) with 810 nm laser treatment with different time by CLSM. Scale bar: 20 μm.

12.6 Characterization of specific ROS in Hela cells

Hela cells were seeded into 35 mm confocal dishes at a density of about 2×10^5 cells per well and incubated overnight at 37 °C in a 5% CO_2 incubator. Upon treatment with of MOC-88 (1.0 μM) for 3 h, these cells were irradiated with/without a 450-460 nm laser (20 mW/cm^2, 10 min). Then the cells were carefully rinsed twice with PBS and incubated with SOSG (10 μM, $^1O_2$), DHE (10 μM, $O_2^{\cdot-}$), HPF (10 μM, ·OH) and BODIPY 581/591 C11 (2 μM, Lipid ROS) in FBS-free DMEM for 15 min and ROSGreen^{TM} H_2O_2 Probe (5 μM, H_2O_2) in HBSS with 20 mM Hepes for 15 min in the dark. SOSG, HPF and ROSGreen^{TM} H_2O_2 Probe: λex = 488 nm, λem = 520 ± 20 nm. DHE: λex = 514 nm, λem = 610 ± 20 nm. BODIPY 581/591 C11 (Red): λex = 561 nm, λem = 591 ± 20 nm. BODIPY 581/591 C11 (Ox): λex = 488 nm, λem = 520 ± 20 nm.

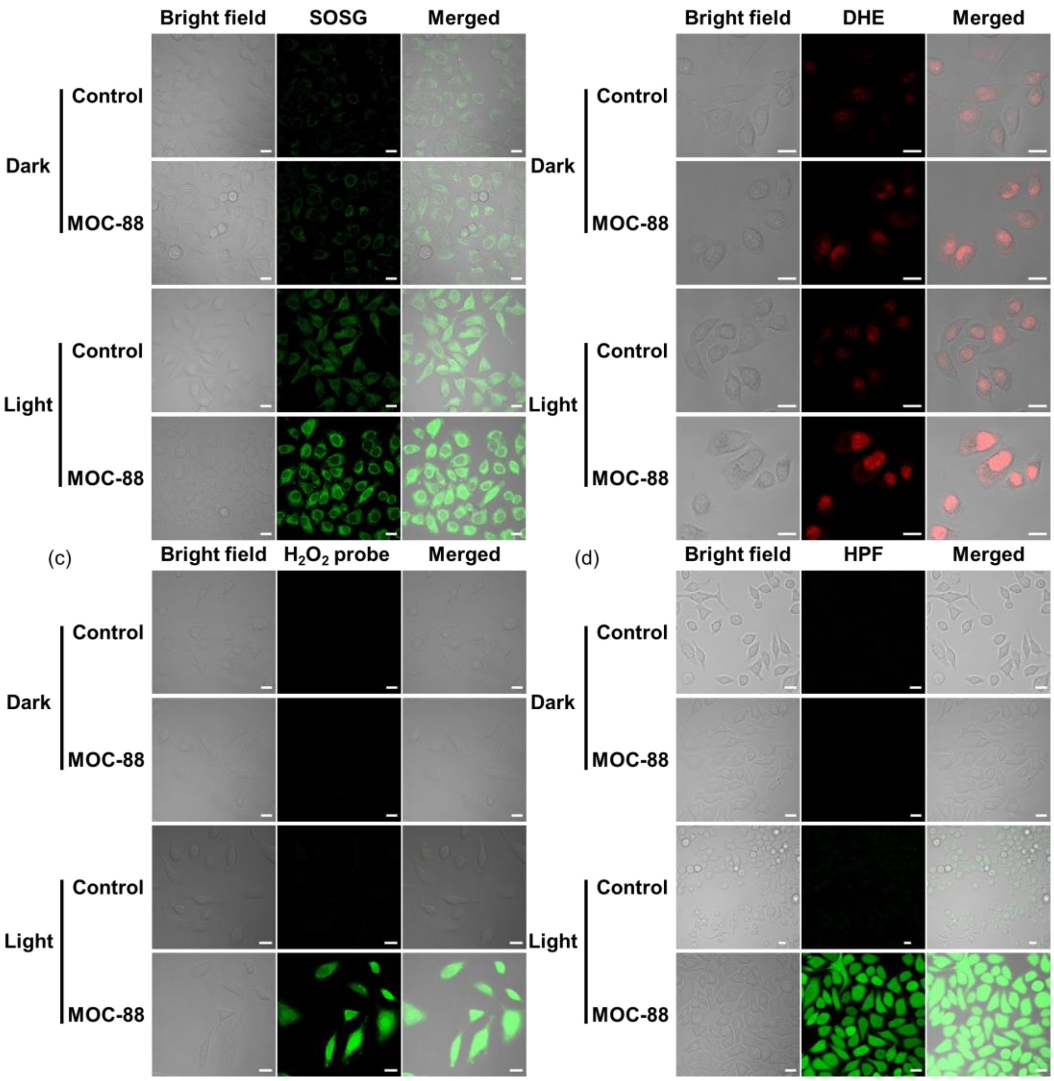

Figure S33. Intracellular SOSG ($^1O_2$), DHE ($O_2^{\cdot-}$), ROSGreen™ H_2O_2 Probe (H_2O_2), HPF (·OH) production of Hela cells after MOC-88 (1.0 μM) with or without light (450-460 nm, 20 mW/cm^2) treatment by CLSM. SOSG, HPF and ROSGreen™ H_2O_2 Probe: λex = 488 nm, λem = 520 ± 20 nm. DHE: λex = 514 nm, λem = 610 ± 20 nm. Scale bar: 20 μm.

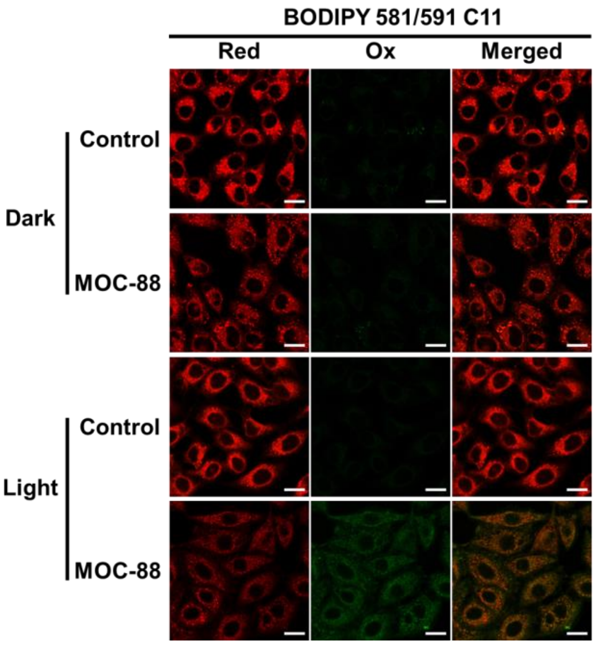

Figure S34. Intracellular Lipid ROS production of Hela cells after MOC-88 (1.0 μM, 3h) with or without light (450-460 nm, 20 mW/cm^2) treatment by CLSM. Scale bar: 20 μm.

12.7 Real-time monitoring of subcellular organelle changes by PDT treatment

Hela cells were seeded into 35 mm confocal dishes at a density of about 2 × 10^5 cells per well and incubated overnight at 37 °C in a 5% CO_2 incubator. Upon treatment with of MOC-88 (1.0 μM) for 3 h, then the cells were carefully rinsed twice with PBS and incubated with Hoechest 33342, MTG, LTDR and Dil in FBS-free DMEM for 15 min and irradiated with light (450-460 nm, 20 mW/cm^2). The cells were immediately visualized after PDT treatment by confocal microscopy.

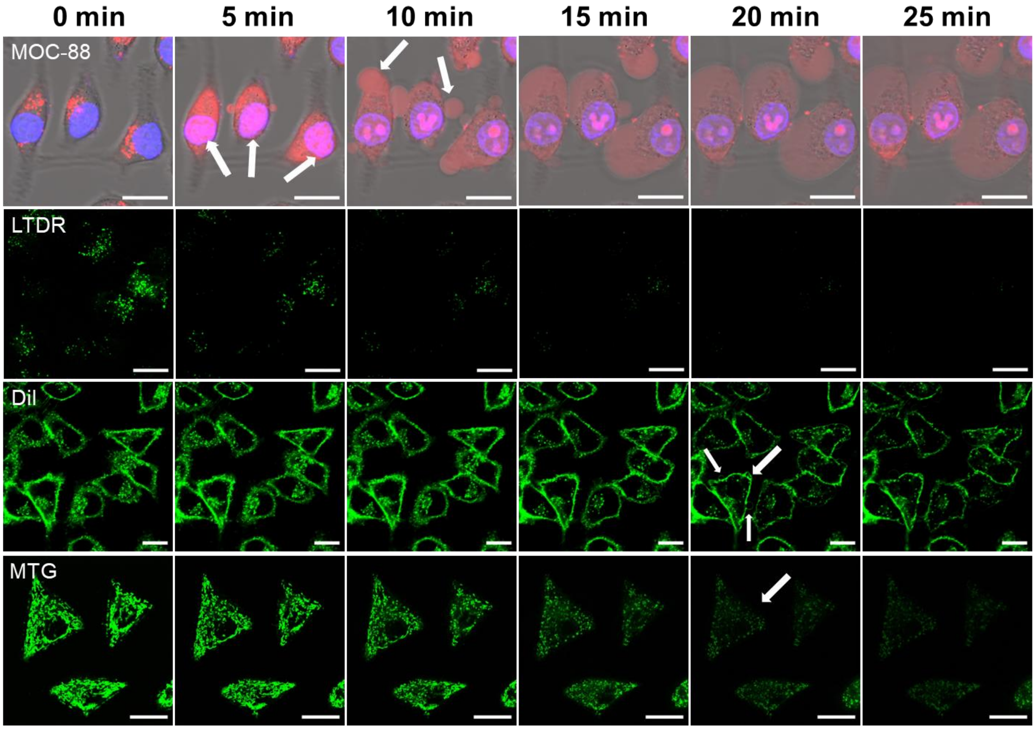

Figure S35. Real-time monitoring of subcellular organelle changes by PDT treatment. Scale bar: 20 μm.

12.8 Detection of cathepsin B release

Cathepsin B activity was detected using the fluorogenic susbtrate Magic Red MR-$(RR)_2$ (Immunochemistry Tech, Bloomington, USA) according to the manufacturer's instructions. Briefly, Hela cells seeded into dishes were exposed to MOC-88 (1.0 μM) at the indicated concentrations for 3 h and then irradiated with a 450-460 nm LED light array (20 mW/cm^2) for 10 min. The media was removed and the cells were washed twice with PBS and then incubated with cathepsin B substrate at 37 °C for 1 h. The media was removed and the cells were washed twice with PBS and visualized by confocal microscopy. Magic Red MR-$(RR)_2$: λex = 543 nm, λem = 630 ± 20 nm.

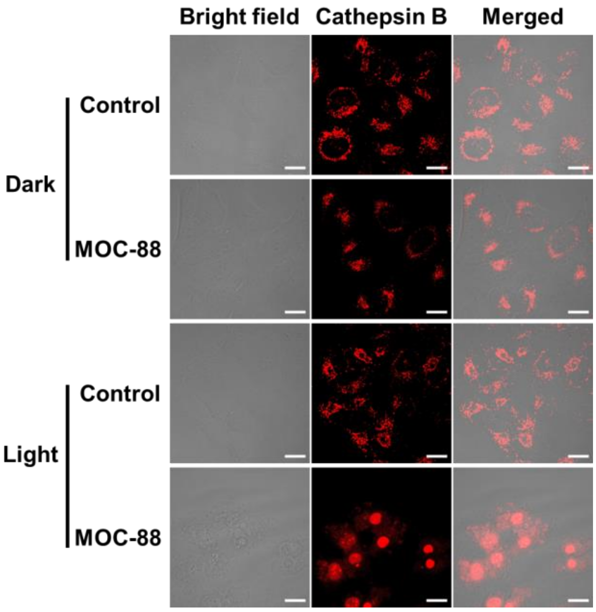

Figure S36. Cathepsin B release of Hela cells after MOC-88 (1.0 μM, 3h) with or without light (450-460 nm, 20 mW/cm^2) treatment by CLSM. Scale bar: 20 μm.

12.9 Detection of ROS content in mitochondria and mitochondrial dsDNA leakage

Hela cells were seeded into 35 mm confocal dishes at a density of about 2 × 10^5 cells per well and incubated overnight at 37 °C in a 5% CO_2 incubator. Upon treatment with of MOC-88 (1.0 μM) for 3 h, these cells were irradiated with/without a 450-460 nm laser (20 mW/cm^2, 10 min). Then the cells were carefully rinsed twice with PBS and incubated with Mito Tracker Red CM-H_2XRos (500 nM, MTO) in FBS-free DMEM for 15 min. Mito Tracker Red CM-H_2XRos: λex = 561 nm, λem = 600 ± 20 nm.

Hela cells were seeded into 35 mm confocal dishes at a density of about 2 × 10^5 cells per well and incubated overnight at 37 °C in a 5% CO_2 incubator. Upon treatment with MOC-88 (1.0 μM) and vehicle control at the indicated concertration for 3 h, then the cells were carefully rinsed twice with PBS and incubated with Mito Tracker Orange. These cells were irradiated with or without a 450-460 nm laser (20 mW/cm^2, 10 min). After 0.5 h's incubation, the cells were washed for three times with sterile PBS and then fixed using 4 % PFA fix solution for 15min. The cells were then permeabilized with PBS solution of 0.1% triton X-100 for 30 min and then blocked with immunostaining blocking buffer for 30 min, and then incubated with monoclonal to anti-dsDNA solution overnight at 4 °C. The cells were then washed with PBS and incubated with secondary

antibodies with Alexa Fluor® 647 next day. Alexa Fluor® 647 antibodies: λex = 633 nm, λem = 665 ± 20 nm.

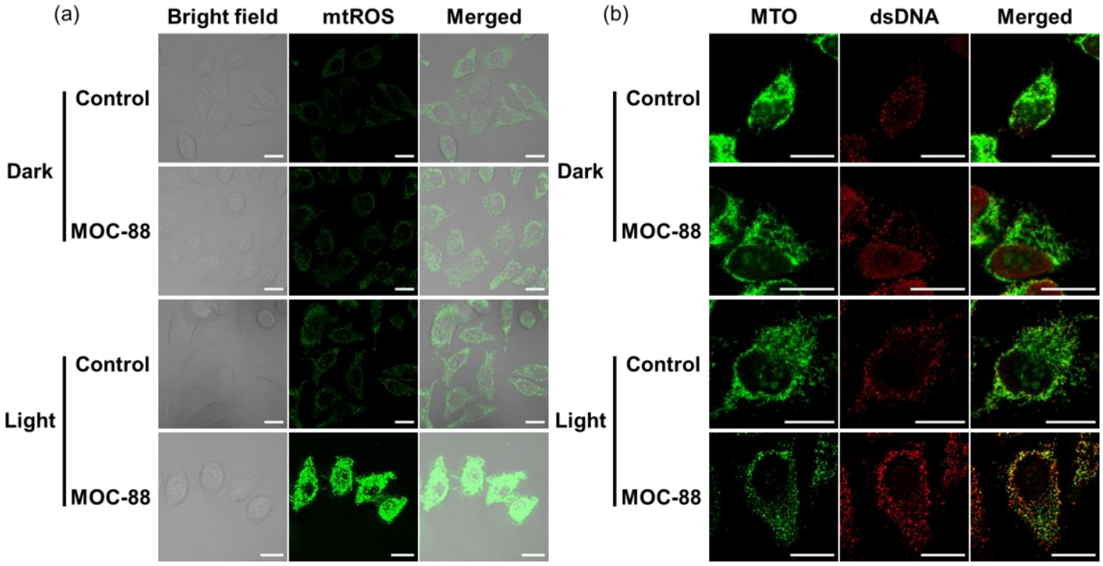

Figure S37. (a) Intracellular mtROS production and (b) dsDNA leakage of Hela cells after MOC-88 (1.0 μM, 3h) with or without light (450-460 nm, 20 mW/cm^2) treatment by CLSM. Scale bar: 20 μm.

12.10 Intracellular lipid peroxidation detection in Hela cells

According to the manufacturer's directions, The intracellular lipid peroxidation levels were measured by Lipid Peroxidation MDA Assay Kit, Hela cells were seeded onto 10 cm dishes and were grown in complete DMEM in a 5% CO_2 incubator with atmosphere at 37 °C overnight. The cells were incubated with MOC-88 at the indicated concentrations for 3 h in dark, and then irradiated with a 450-460 nm laser (20 mW/cm^2, 10 min). After 0.5 hours' incubation, the cells were washed for three times with sterile PBS. Then the cells were collected, lysed by cell lysis buffer and centrifuged at 14,000 × g at 4 °C for 15 min. The supernatants were reacted with thiobarbituric acid (TBA) at 100 °C for 15 min, and the reaction products which were expressed as μmol/mg protein, were measured at 532 nm using a microplate spectrophotometer. The experiment was repeated three times.

12.11 Measurement of NADP$^+$/NADPH and GSSG/GSH Ratios

Hela cells were seeded onto 10 cm dishes and were grown in complete DMEM in a 5% CO_2 incubator with atmosphere at 37 °C overnight. The cells were incubated with MOC-88 at the indicated concentrations for 3 h in dark, and then irradiated with a 450-460 nm laser (20 mW/cm^2, 10 min). After 0.5 hours' incubation, the cells were washed for three times with sterile PBS. The NADP$^+$/NADPH and GSSG/GSH ratios were measured using the NADP$^+$/NADPH Assay Kit with WST-8 (Beyotime, China) and the GSH and GSSG Assay Kit (Beyotime, China) following the manual protocols, respectively.

12.12 Mitochondrial Membrane Potential Assays

Mitochondrial membrane potential was measured by JC-1 assay. Briefly, Hela cells were seeded in 6-well plates for 24 h. The cells were treated with MOC-88 with different concentration for 3 h, and then irradiated with or without a 450-460 nm laser (20 mW/cm^2, 10 min). then the JC-1 was added and then incubated for 20 min at 37 °C. Then cells were washed for 2~3 times with

ice-cold assay buffer. λex = 488 nm, λem = 535 ± 20 nm (JC-1 monomers) and λem = 570 ± 20 nm (JC-1 aggregates). 10^4 cells were analyzed for each sample and the data were analyzed using FlowJo 7.6.1 software.

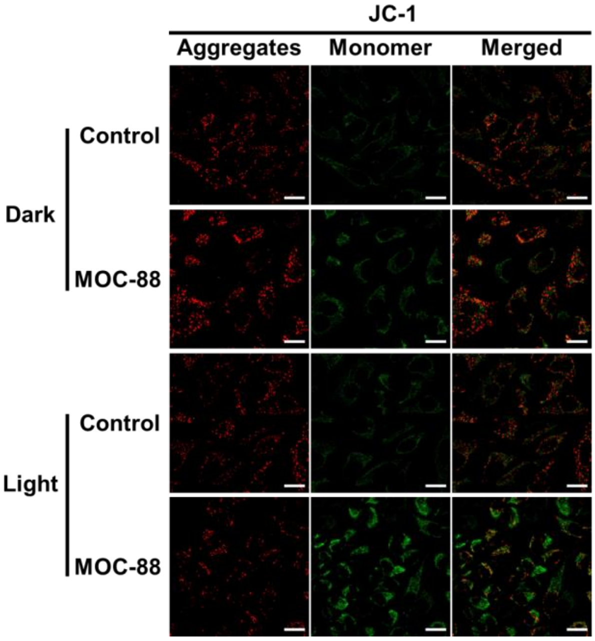

Figure S38. Effects of MOC-88 on MMP measured by JC-1 staining of Hela cells after MOC-88 (1.0 μM, 3h) with or without light (450-460 nm, 20 mW/cm^2) treatment by CLSM. Scale bar: 20 μm.

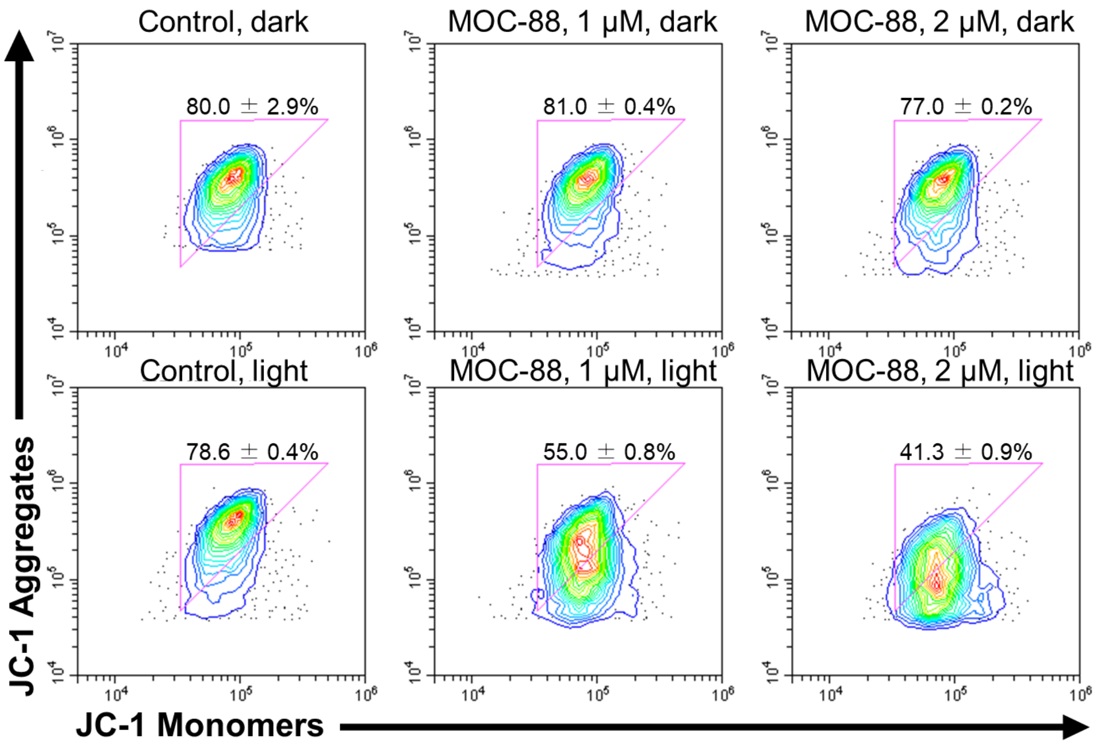

Figure S39. Effects of MOC-88 on MMP measured by JC-1 staining of Hela cells after MOC-88 with or without light (450-460 nm, 20 mW/cm^2) treatment by flow cytometry (mean ± SD, n = 3).

12.13 p65 and p-IRF3 enters the nucleus

Hela cells were seeded into 35 mm confocal dishes at a density of about 2 × 10^5 cells per well and incubated overnight at 37 °C in a 5% CO_2 incubator. Upon treatment with MOC-88 (1.0 μM) and vehicle control at the indicated concertration for 3 h. These cells were irradiated with or without a 450-460 nm laser (20 mW/cm^2, 10 min). After 0.5 h's incubation, the cells were washed for three times with sterile PBS and then fixed using 4 % PFA fix solution for 15min. The cells were then permeabilized with PBS solution of 0.1% triton X-100 for 30 min and then blocked with immunostaining blocking buffer for 30 min, and then incubated with monoclonal to anti-p65 or anti-IRF3 solution overnight at 4 °C. The cells were then washed with PBS and incubated with secondary antibodies with Alexa Fluor® 488 next day. Alexa Fluor® 488 antibodies: λex = 488 nm, λem = 510 ± 20 nm

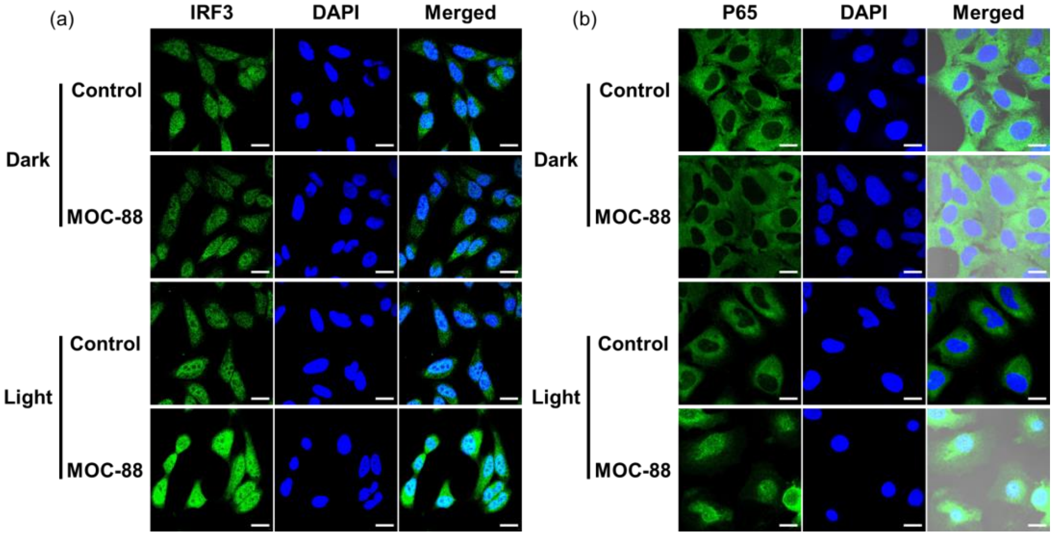

Figure S40. (a) P65 and (b) IRF3 enters the nucleus of Hela cells after MOC-88 (1.0 μM, 3h) with or without light (450-460 nm, 20 mW/cm^2) treatment by CLSM. Scale bar: 20 μm.

12.14 Transmission Electron Microscope

Hela cells were seeded in 10 cm culture dishes for 24 h, and then treated with MOC-88 (1.0 μM) for 3 h. The cells were trypsinized, washed with PBS and fixed with glutaric dialdehyde. The samples were stained with Osmium tetroxide before imaging. All the images were obtained by the Eversmart Jazz program (Scitex).

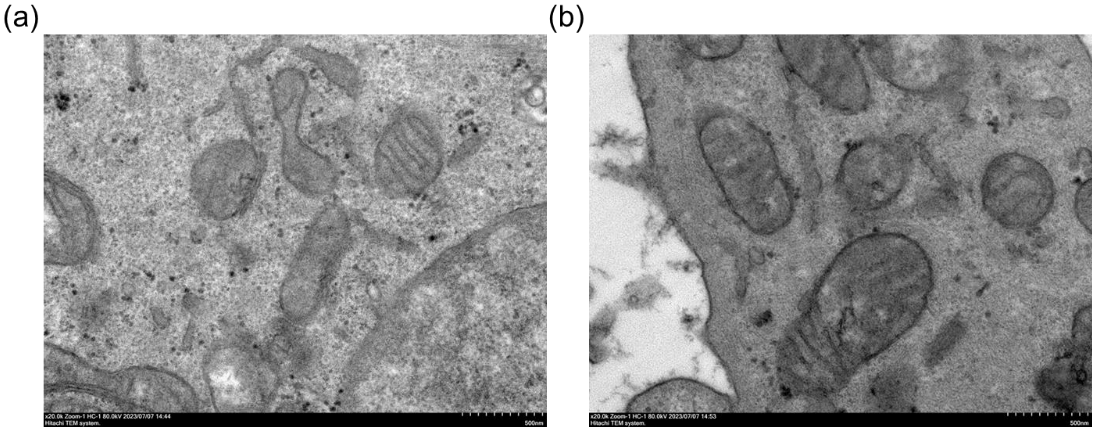

Figure S41. Representative TEM images of Hela cells. Incubation with MOC-88 (1.0 μM, 3h) (a) dark and (b) exposure to irradiation.

12.15 Extracellular ATP, Release of LDH and Cytokine measurement

Hela cells were seeded onto 6 cm dishes and were grown in complete DMEM in the cell incubator with 5% CO_2 atmosphere at 37 °C for 24 h. The cells were incubated with MOC-88 at the indicated concentrations for 3 h in dark, and then irradiated with or without a 450-460 nm laser (20 mW/cm^2, 10 min). After 0.5 hour's incubation, the extracellular ATP concentration was measured by luciferase luminescence assay using an ATP assay kit, LDH was measured using LDH Cytotoxicity Assay Kit (Beyotime, China) and The concentrations of Human IL-1β and IL-18 in various sample supernatants were determined using Human IL-1β ELISA kit and Human IL-18 ELISA kit.

12.16 Immunogenic Cell Death

Hela cells were seeded into 35 mm confocal dishes and incubated overnight at 37 °C in a 5% CO_2 incubator. Upon treatment with MOC-88 (1.0 μM) and vehicle control at the indicated concertration for 3 h, they were irradiated with or without a 450-460 nm laser (20 mW/cm^2, 10 min). After 0.5 h's incubation, the cells were washed for three times with sterile PBS and then fixed using 4 % PFA fix solution for 15min. The cells were then permeabilized with PBS solution of 0.1% triton X-100 for 15 min and then blocked with immunostaining blocking buffer for 30 min, and then incubated with Alexa Fluor® 488 Rabbit monoclonal to HMGB1 or Alexa Fluor® 647 Rabbit monoclonal to Calreticulin (CRT) solution overnight at 4 °C. They were then incubated with DAPI for 15 min after being washed with PBS and analyzed by confocal microscopy. Alexa Fluor® 488 Rabbit monoclonal to HMGB1: λex = 488 nm, λem = 520 ± 20 nm; Alexa Fluor® 647 Rabbit monoclonal to Calreticulin (CRT): λex = 633 nm, λem = 665 ± 20 nm.

Hela cells were seeded in 6-well plates and incubated for 24 h, and the cells were then treated with MOC-88 for 3 h. The cells were collected, washed with PBS, and fixed with 4% paraformaldehyde for 10 min at room temperature. For intracellular HMGB1 analysis, the cells were permeated with 0.4% Triton X-100 for 10 min at room temperature before incubation with HMGB1 primary antibody for 12 h at 4 °C. For membranous CRT amslysis, the cells (without permeabilization) were directly incubated with CRT primary antibody for 12 h at 4 °C. All the cells then were analyzed on a flow cytometer. λex= 488 nm (HMGB1) and 633 nm (CRT); λem= 525 ± 30 nm (HMGB1, FITC channel, green) and 680 ± 30 nm (CRT, PE channel, red).

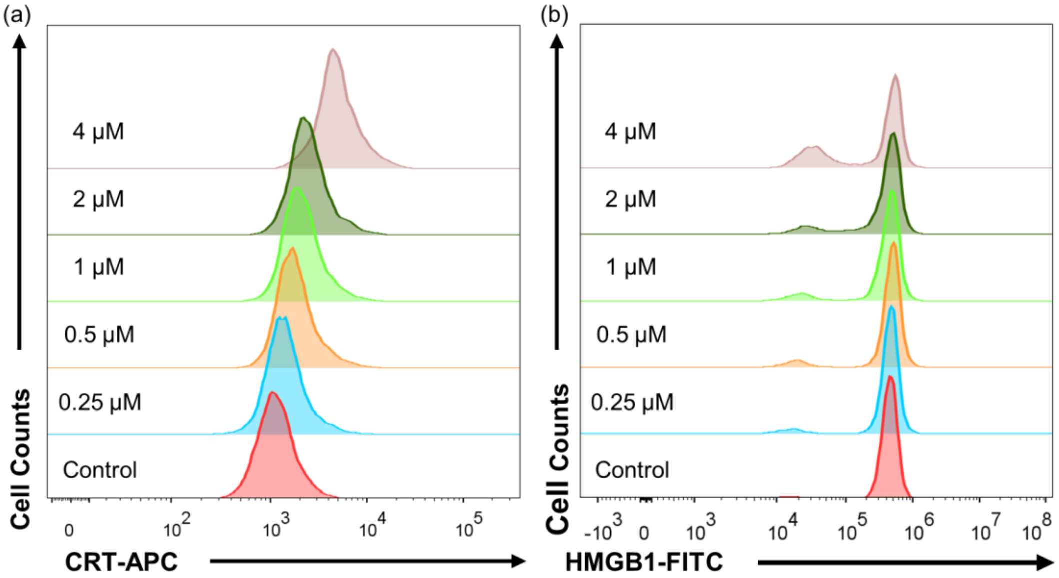

Figure S42. Flow cytometric analysis of the expression of CRT on cell membrane and HMGB1 in Hela cells after MOC-88 (1.0 μM, 3h) with light (450-460 nm, 20 mW/cm^2) treatment.

12.17 Western Blotting

Hela cells were seeded in 6-well plates for 24 h, then treated with the MOC-88 for 3 h. After then, cells were trypsinized and washed with ice-cold PBS twice. The harvested cells were lysed by RIPA buffer with inhibitors of proteases (Roche Diagnostics GmbH, Mannheim, Germany) and inhibitor of phosphatases sodium orthovanadate (Sigma Aldrich, Missouri, USA) as supplement. The protein concentration was quantified using the BCA Quantitation Kit. Equal amounts of cellular lysate were separated on SDS polyacrylamide gel electrophoresis and then transferred to polyvinylidene difluoride membranes. The membranes were incubated in 5% (w/v) skim milk in TBST buffer [100 mM NaCl, 10 mM TrisHCl (pH 7.6) and 0.1% (v/v) Tween-20] at room temperature for 1 h, and further incubated in the presence of the primary antibody overnight at 4˚C. The membranes were then washed with TBST and incubated with secondary antibodies next day. The protein expression levels were detected using Clarity Max Western ECL Subetrate (BIO-RAD) and imaged using by means of a Tanon 5200 Imager.

Table S4 Calculate the grayscale values of each imprint relative to the internal reference

	Dark		Light		
Concentration (μM)	0	0.5	0	0.25	0.5
cGAS/β-actin	1.00	0.85	1.08	1.10	1.46
p-STING/STING	1.00	1.22	1.33	2.71	2.90
p-TBK1/TBK1	1.00	2.21	1.24	11.53	12.97
p-IRF3/IRF3	1.00	0.86	1.06	1.46	1.60
p-IκBα/IκBα	1.00	0.69	0.73	1.96	2.54
p-p65/p65	1.00	0.77	0.81	1.13	1.25
AIM2/Vinculin	1.00	0.52	0.98	1.79	3.08
NLRP3/Vinculin	1.00	1.56	1.91	2.11	2.55
ASC/β-actin	1.00	1.39	1.96	2.42	3.21
C-CASP1/CASP1	1.00	1.18	1.22	1.36	1.72

GSDMD-N/GSDMD	1.00	1.09	0.91	1.55	1.48
GSDME-N/GSDMD	1.00	1.24	1.25	1.37	1.45
p-RIPK1/RIPK1	1.00	0.89	0.77	8.12	21.86
p-MLKL/MLKL	1.00	1.15	1.18	1.32	2.52
C-CASP3/CASP3	1.00	0.81	1.00	0.91	1.10
C-CASP7/CASP7	1.00	0.87	0.84	1.51	1.20
C-CASP8/CASP8	1.00	1.45	1.29	1.77	2.23
C-CASP9/CASP9	1.00	1.10	1.24	1.36	1.30
GPX4/β-actin	1.00	0.76	0.90	0.76	0.57

13. In *vivo* Antitumor Assay

All animal operations were in accord with guidelines of Institute of Zoology, Guangdong Academy of Sciences. The accreditation number is GIZ20230327. Pathogen-free female BALB/c mice, 4-5 weeks of age, were purchased and bred in the Institute of Zoology, Guangdong Academy of Sciences. The BALB/c female mice were randomly divided into 6 groups (control + dark, $RuL^{oz}_3(BF_4)_2$ + dark, MOC-88 + dark, control + light, $RuL^{oz}_3(BF_4)_2$ + light and MOC-88 + light), and each group contains 5 mice. U14 cells were subcutaneously inoculated into the BALB/c mice. When the primary tumors reach the size of 70 mm^3, $RuL^{oz}_3(BF_4)_2$ (4.24 mg/kg) and MOC-88 (5 mg/kg) were intratumorally injected. After 24 h, the laser groups were exposed to an 810nm two-photon laser for 10 min. The mice were routinely monitored for tumor volumes and body weight. The tumor volume (V) was calculated as Volume (V) = $width^2 \times length / 2$. The mice were sacrificed on day 14. The major organs (including heart, liver, spleen, lung and kidney) of each group were further studied by hematoxylin and eosin (H&E) staining assay.

To systematically investigate the in *vivo* anti-tumor immunity, a series of immune cells were analyzed by flow cytometry after staining with antibodies. The tumors and tumor draining lymph nodes were obtained from the mices (n = 3 mice in each group) and were made into a single cell suspension with 1 mg/mL collagenase IV and 0.2 mg/mL DNase I for 1 h at 37 °C. After being filered through a 40 μm nylon cell strainer, the blood samples were removed by red blood cell lysis buffer. The cells were washed with PBS and stained with FITC anti-CD3 (1:400), PE anti-CD4 (1:200), APC anti-CD8a (1:50), FITC anti-CD11b (1:50), PerCP/Cyanine 5.5 anti-F4/80 (1:100), Pacific Blue anti-CD86 (1:100), PE anti-CD86 (1:100), or APC anti-CD80 (1:100) antibodies (obtained from BioLegend) according to the manufacturer's protocols. The cytotoxic T lymphocytes (CTLs, $CD3^+CD4^-CD8^+$), and helper T cells (Ths, $CD3^+CD4^+CD8^-$), tumour-associated M1 macrophages ($F4/80^+CD11b^+CD86^+$) were gated on the flow cytometry.

Serum samples were isolated from mice after above treatments and stored at liquid nitrogen for analysis. And the secretion of pro-inflammatory cytokines (IL-12p70, IL-1β, IL-2, IL-6, IFN-γ and TNF-α), anti-inflammatory cytokine (IL-10) and chemokines (KC/GRO) was measured by MSD according to the manufacturer's protocols.

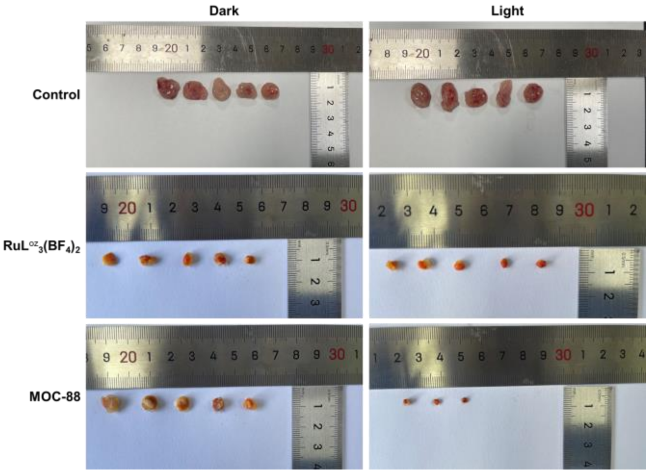

Figure S43. The photographs of the tumor collected at the end of therapeutic period.

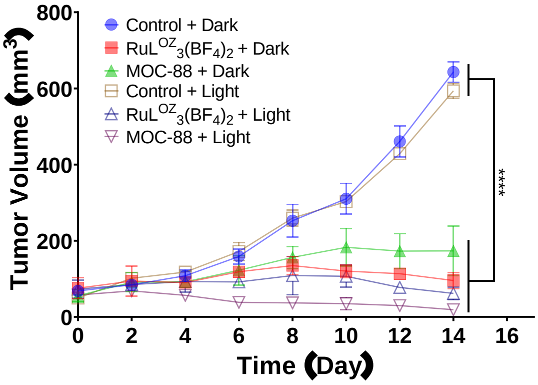

Figure S44. Volume changes of tumors throughout the follow-up period (mean ± SD, *n* = 3). Statistical *P*-value: **P*<0.05, ***P*<0.01, ****P*<0.001, *****P*<0.0001.

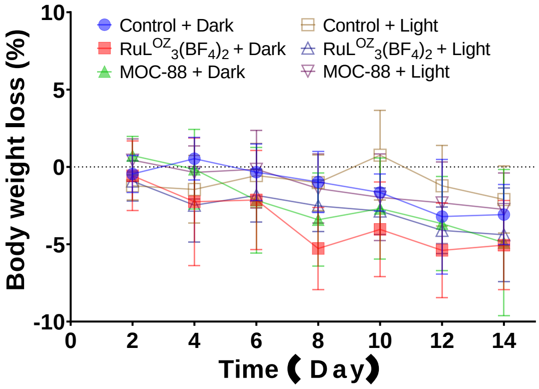

Figure S45. The body weight loss of the mice throughout the follow-up period (mean ± SD, *n* = 3). Statistical *P*-value: **P*<0.05, ***P*<0.01, ****P*<0.001, *****P*<0.0001.

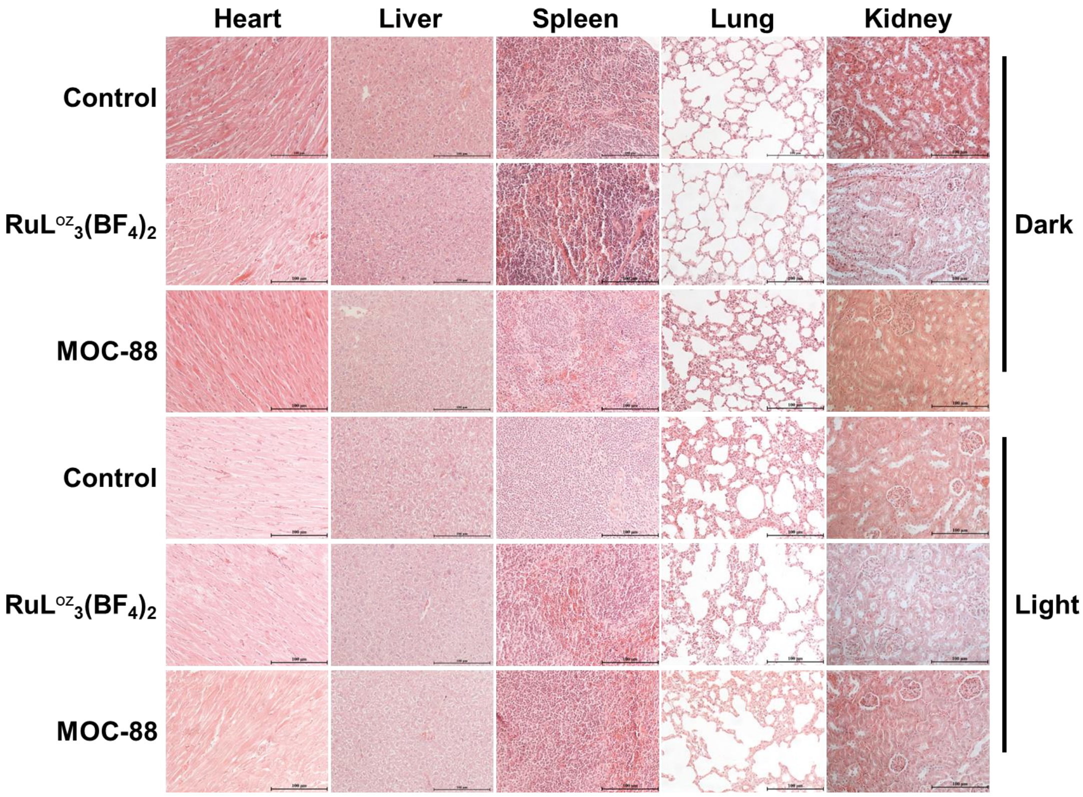

Figure S46. Histological examination of the main organs of the mice after treatment using hematoxylin-eosin (H&E) staining.

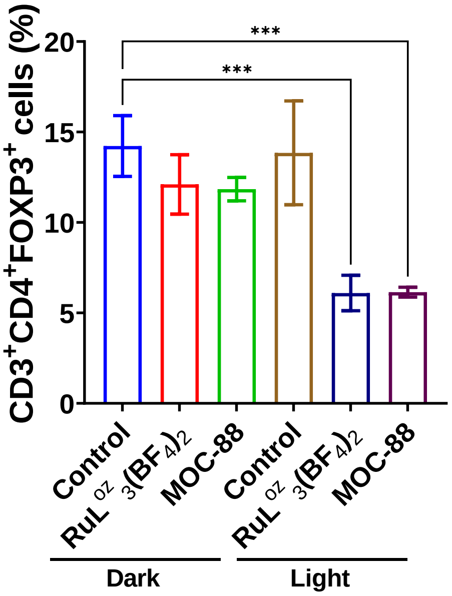

Figure S47. Populations of CD3$^+$CD4$^+$FOXP3$^+$ T cells (Tregs) after treatment in *vivo* (mean ± SD, n = 3) by flow cytometry analysis. Statistical P-value: *P<0.05, **P<0.01, ***P<0.001, ****P<0.0001, by one-way ANOVA.

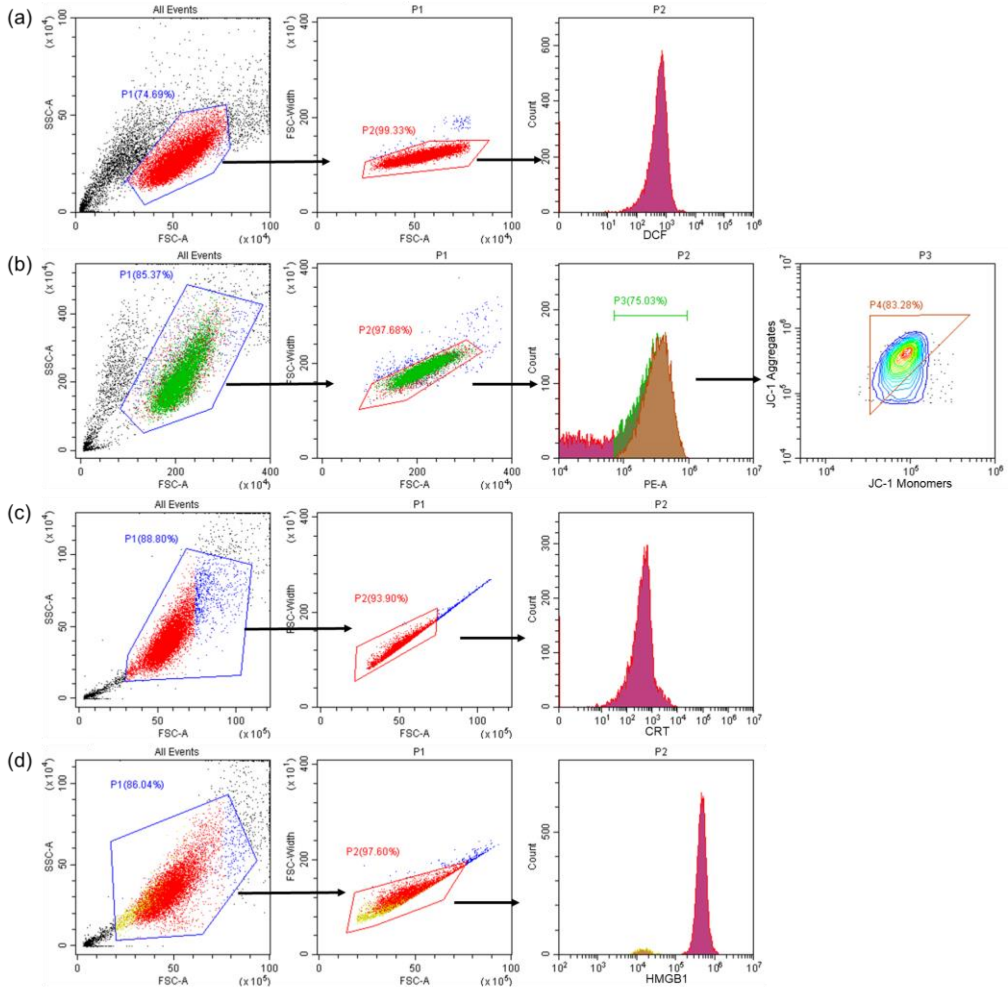

Figure S48. (a) Gating strategy to determine DCF positive rate presented in Fig. S31. (b) Gating strategy to determine JC-1 positive rate presented in Fig. S39. Gating strategy to determine (c) CRT and (d) HMGB1 positive rate presented in Fig. S42.

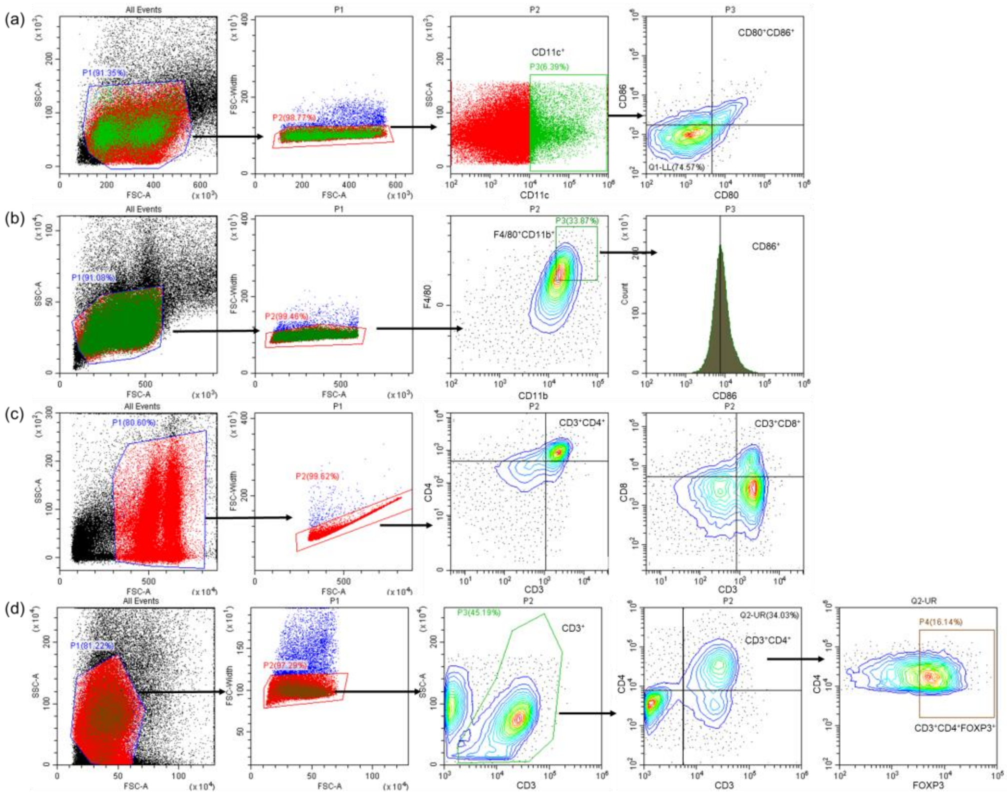

Figure S49. (a) Gating strategy to identify mature DC (CD11b$^+$CD80$^+$CD86$^+$) presented on Fig. 7f. (b) Gating strategy to identify tumour-associated M1 macrophages (F4/80$^+$CD11b$^+$CD86$^+$) presented on Fig. 7g. (c) Gating strategy to identify helper T cells (CD3$^+$CD4$^+$, Ths) and cytotoxic T cells (CD3$^+$CD8$^+$, CTLs) presented on Fig. 7h and 7i. (d) Gating strategy to identify regulatory T cells (CD3$^+$CD4$^+$FOXP3$^+$, Tregs) presented on Fig. S47.

14.Statistical analysis

All quantitative data were presented as the mean ± standard deviation (SD). GraphPad Prism for Windows (GraphPad Software, San Diego, California, USA) was used for all statistical analyses. One-way ANOVA was used to determine statistically significant differences and Dunnett's multiple comparison test was used to determine the significance of the differences. Multiple measurements of quantitative normally distributed data were used for Two-way Repeated-Measures ANOVA, and $P < 0.05$ was considered statistically significant (Statistical *P*-value: *P<0.05, **P<0.01, ***P<0.001, ****P<0.0001).
